# Supplementary figures and images for: TEAD1 is crucial for developmental myelination, Remak bundles, and functional regeneration of peripheral nerves (part 1 of 2)
Source: eLife. 2024 Mar 8;13:e87394. doi: 10.7554/eLife.87394 (PMC10959528; doi:10.7554/eLife.87394)

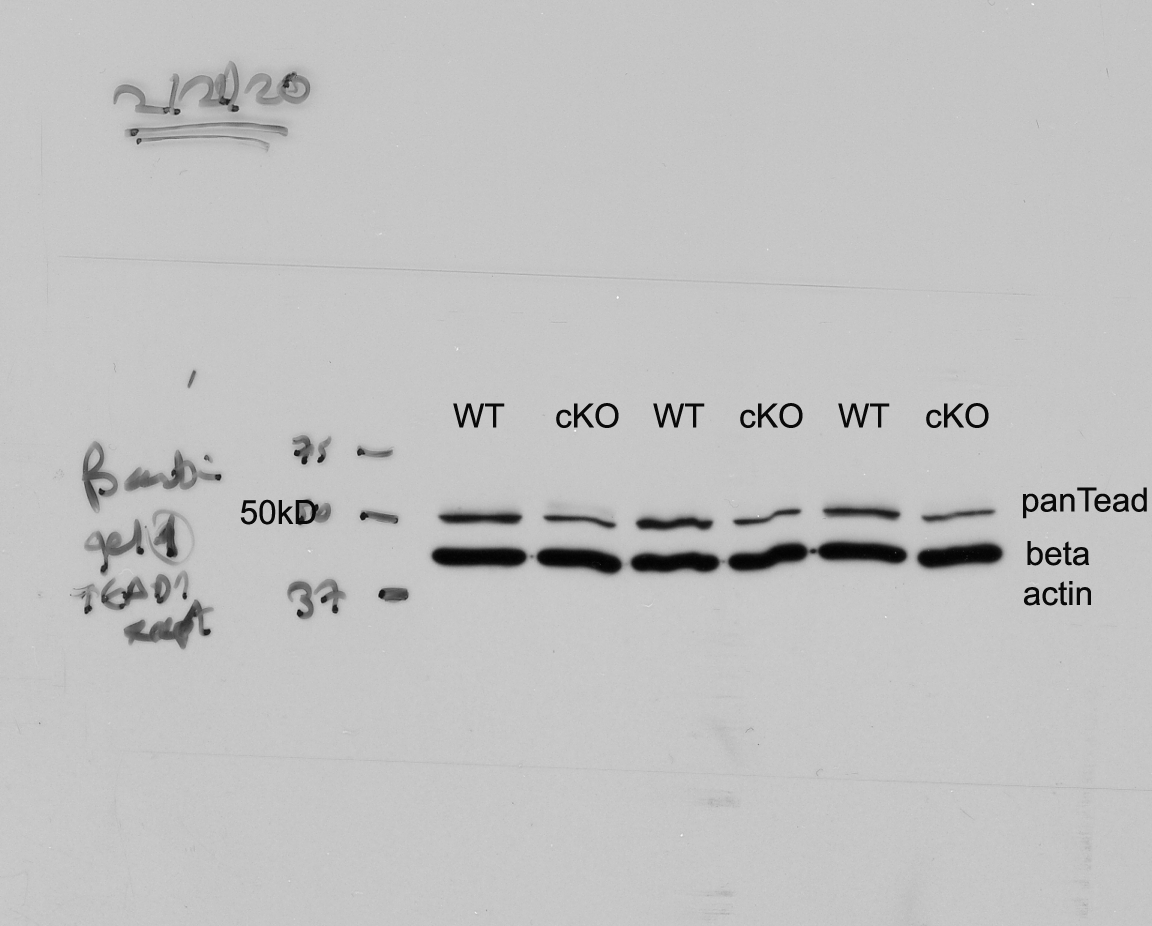

Supplement: Figure 1—source data 1. [file elife-87394-fig1-data1.zip › Fig 1 source data 1/Fig 1B blots and prizm files for graphs/actin previously blotted for panTEAD/uncropped 1 labeled reprobed after panTead.tif]

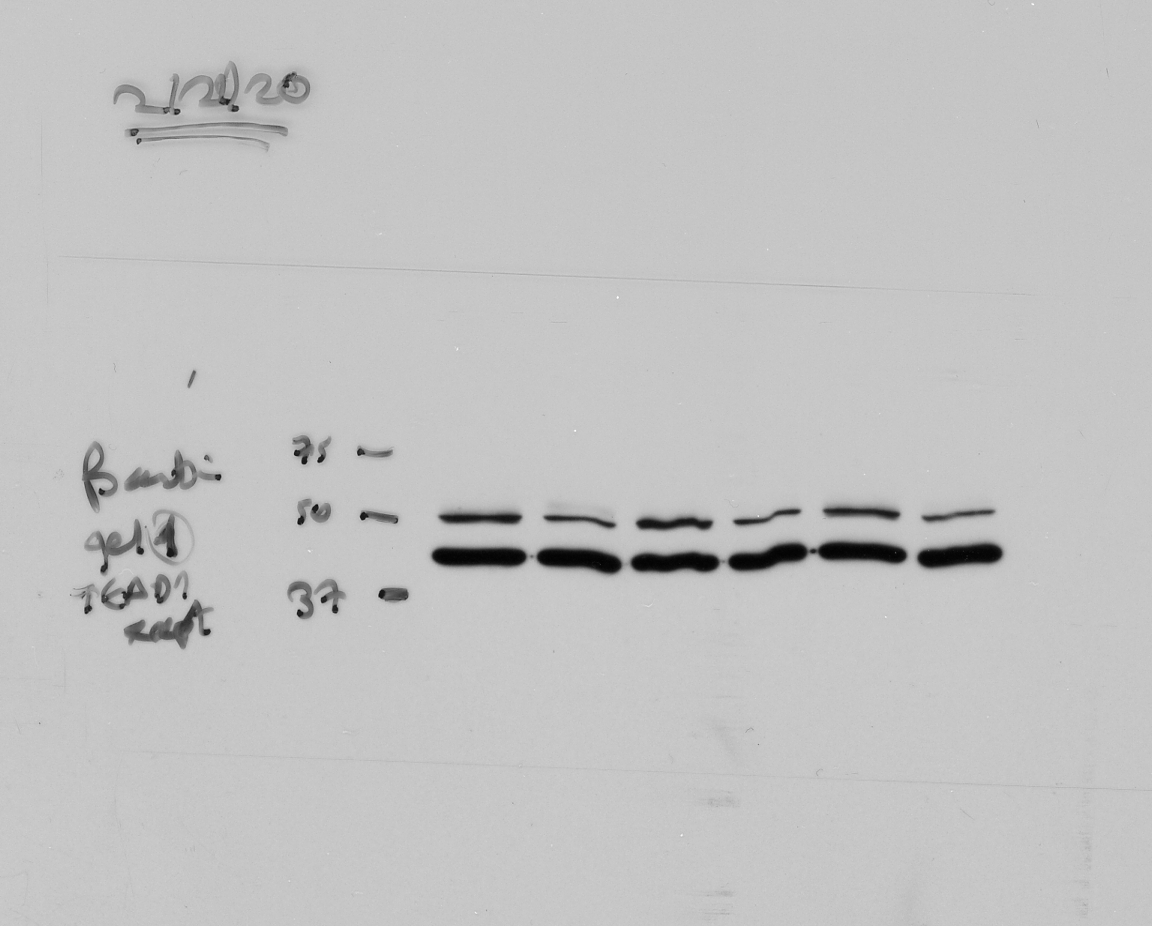

Supplement: Figure 1—source data 1. [file elife-87394-fig1-data1.zip › Fig 1 source data 1/Fig 1B blots and prizm files for graphs/actin previously blotted for panTEAD/uncropped 1.tif]

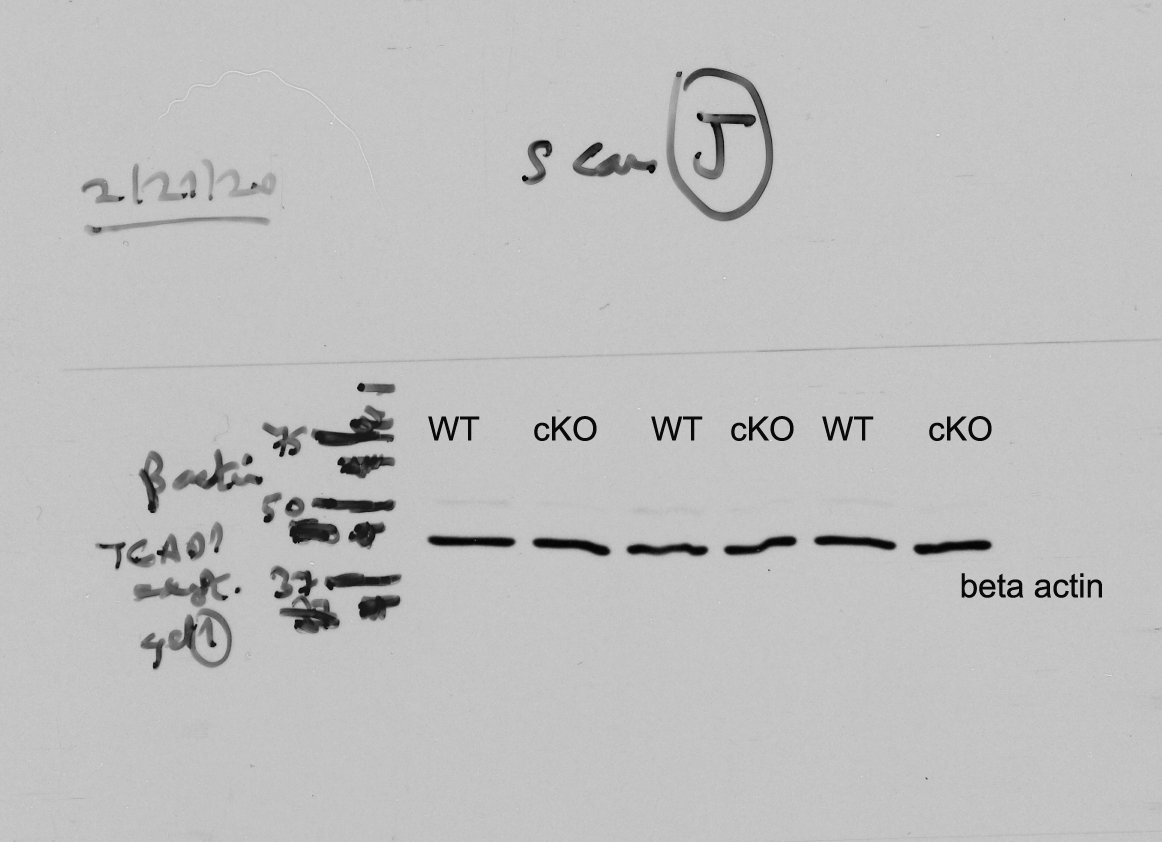

Supplement: Figure 1—source data 1. [file elife-87394-fig1-data1.zip › Fig 1 source data 1/Fig 1B blots and prizm files for graphs/actin previously blotted for panTEAD/uncropped 2 labeled.tif]

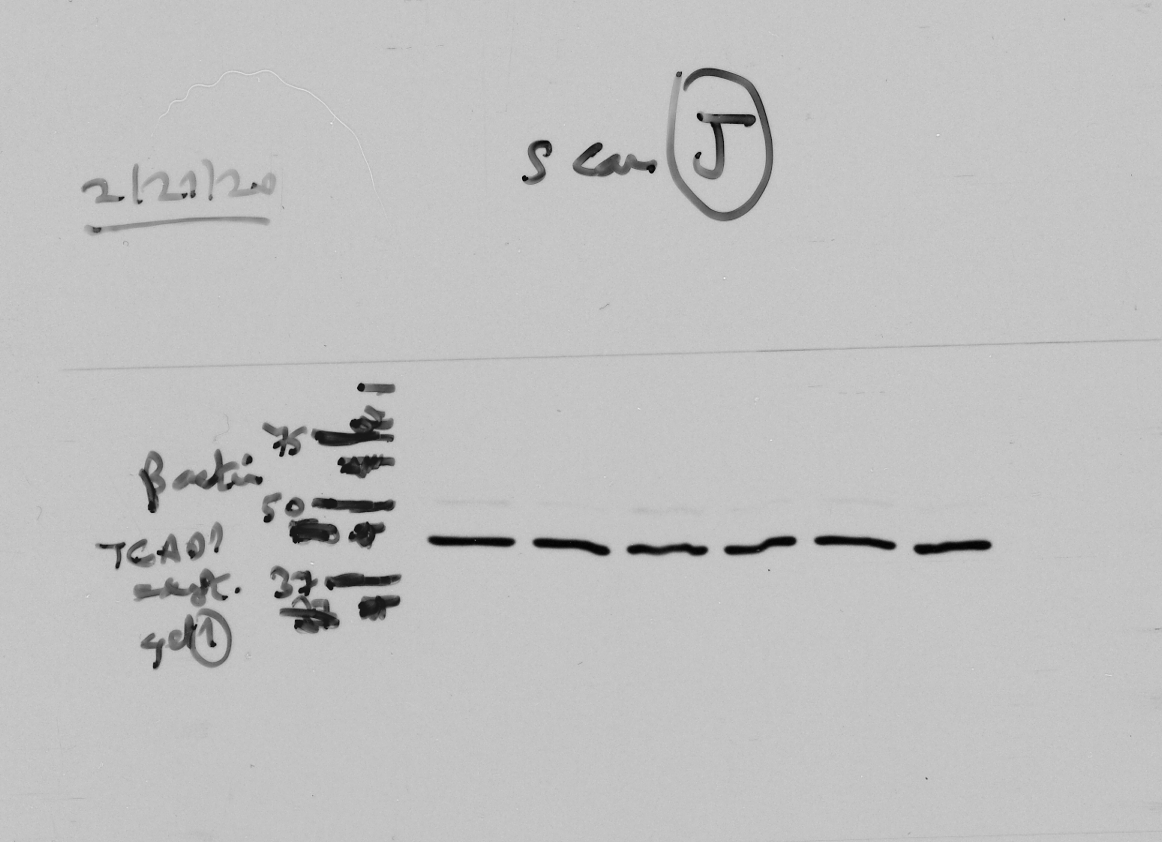

Supplement: Figure 1—source data 1. [file elife-87394-fig1-data1.zip › Fig 1 source data 1/Fig 1B blots and prizm files for graphs/actin previously blotted for panTEAD/uncropped 2.tif]

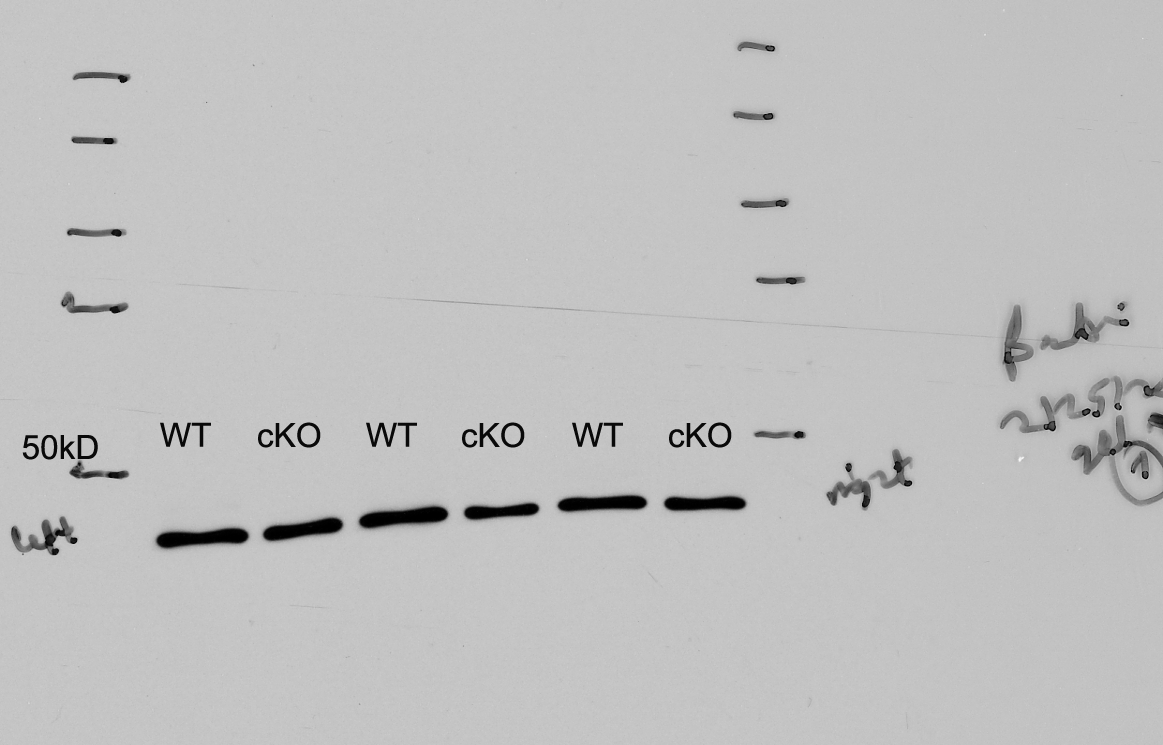

Supplement: Figure 1—source data 1. [file elife-87394-fig1-data1.zip › Fig 1 source data 1/Fig 1B blots and prizm files for graphs/Beta actin for TEADs/uncropped 1 labeled.tif]

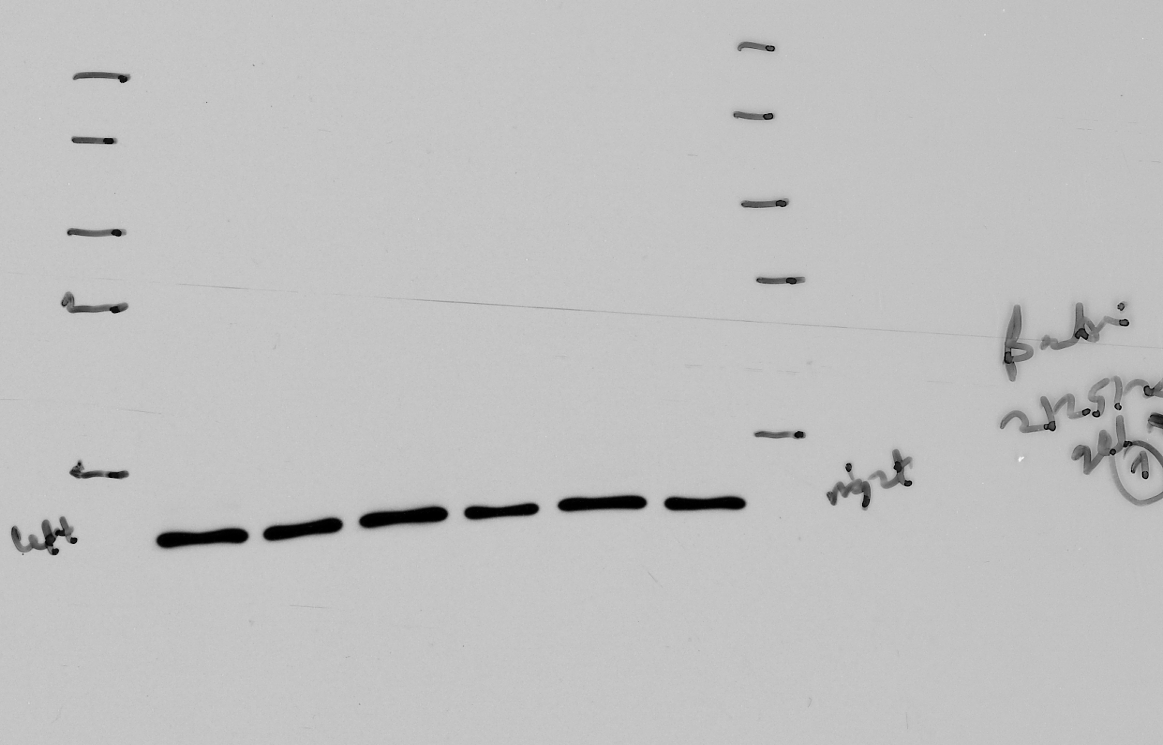

Supplement: Figure 1—source data 1. [file elife-87394-fig1-data1.zip › Fig 1 source data 1/Fig 1B blots and prizm files for graphs/Beta actin for TEADs/uncropped 1.tif]

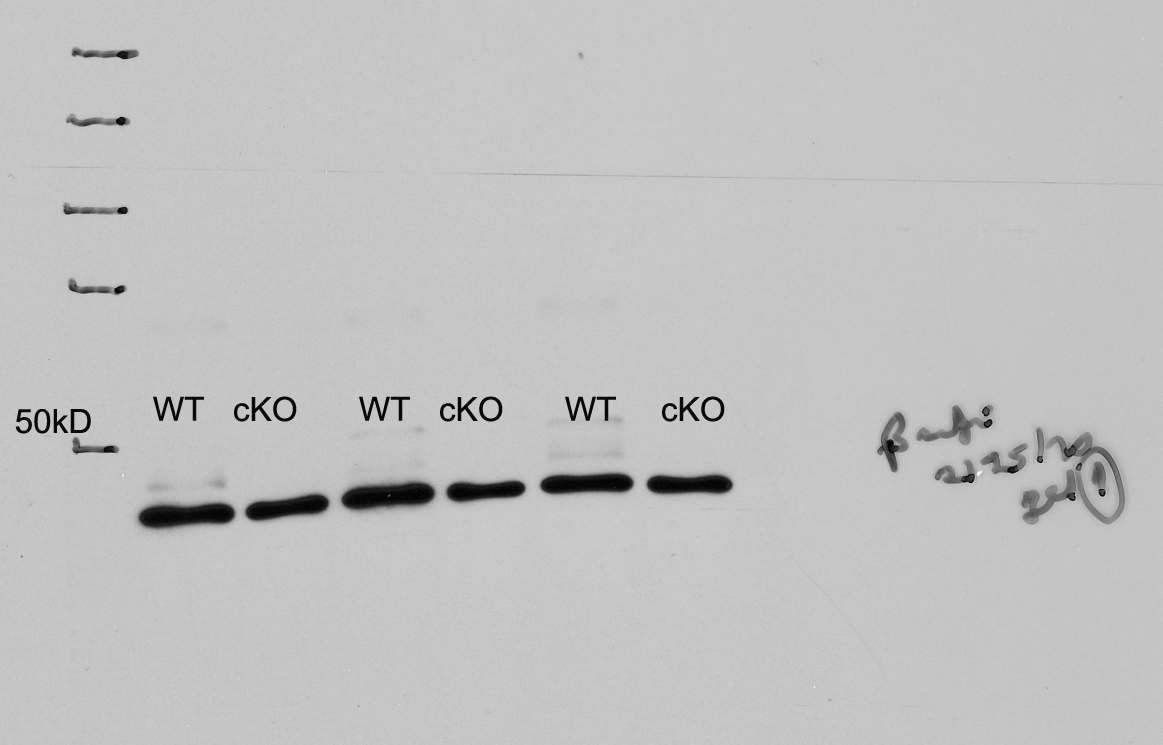

Supplement: Figure 1—source data 1. [file elife-87394-fig1-data1.zip › Fig 1 source data 1/Fig 1B blots and prizm files for graphs/Beta actin for TEADs/uncropped 2 labeled.tif]

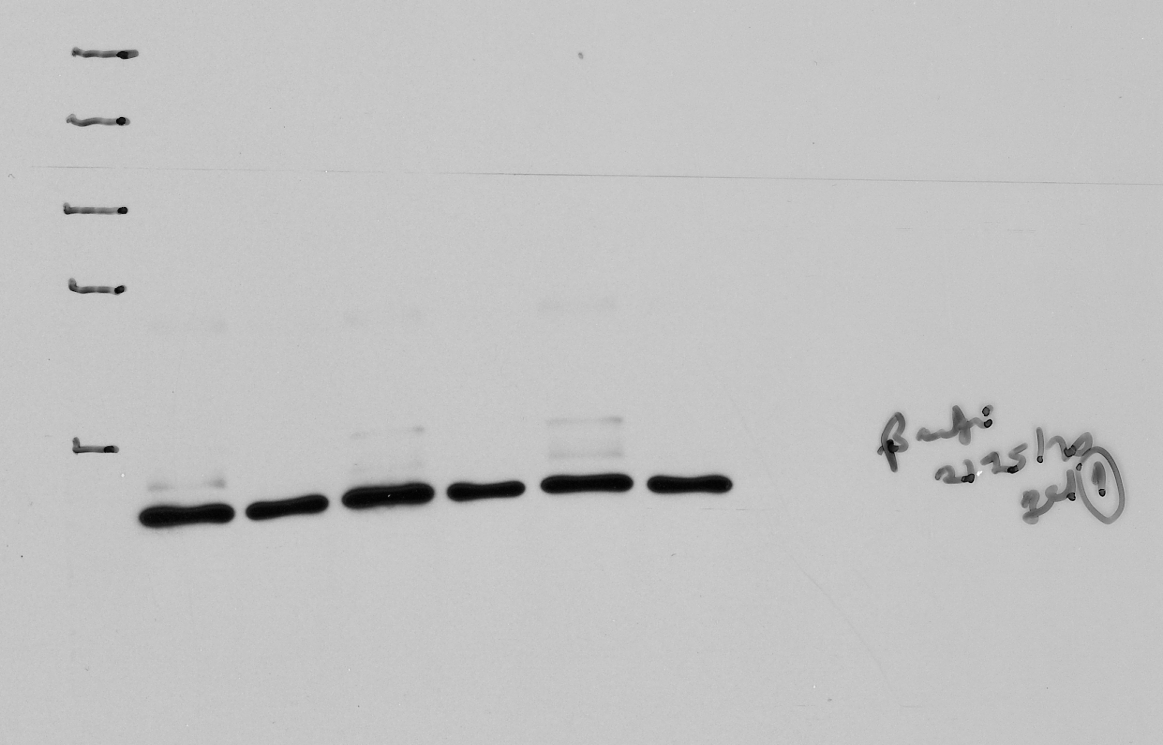

Supplement: Figure 1—source data 1. [file elife-87394-fig1-data1.zip › Fig 1 source data 1/Fig 1B blots and prizm files for graphs/Beta actin for TEADs/uncropped 2.tif]

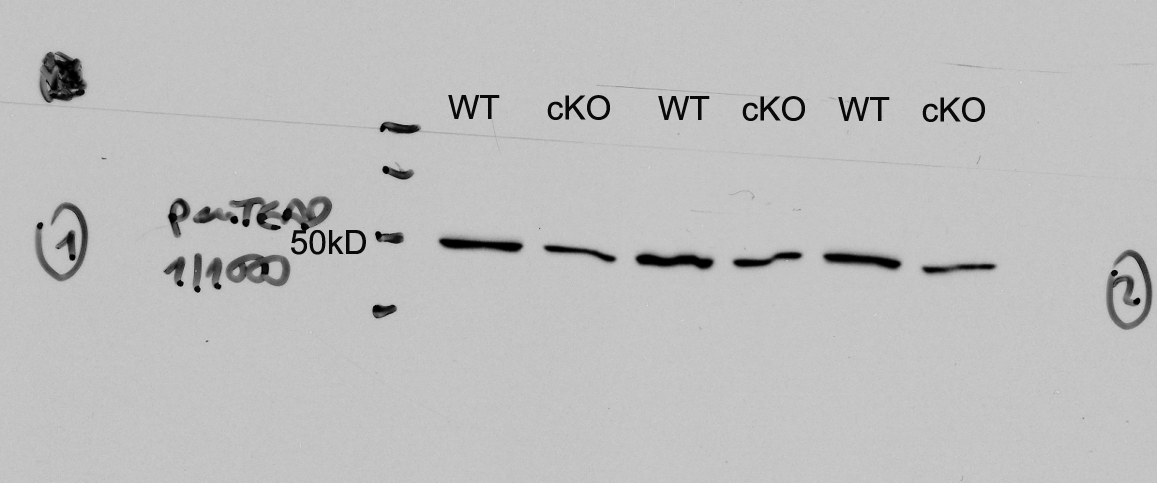

Supplement: Figure 1—source data 1. [file elife-87394-fig1-data1.zip › Fig 1 source data 1/Fig 1B blots and prizm files for graphs/panTEAD/uncropped 1 labeled.tif]

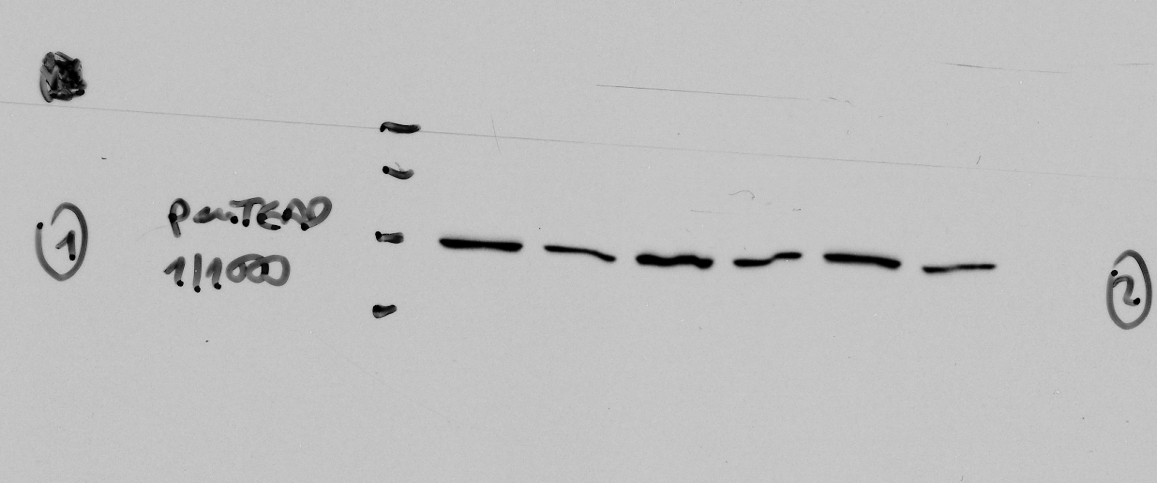

Supplement: Figure 1—source data 1. [file elife-87394-fig1-data1.zip › Fig 1 source data 1/Fig 1B blots and prizm files for graphs/panTEAD/uncropped 1.tif]

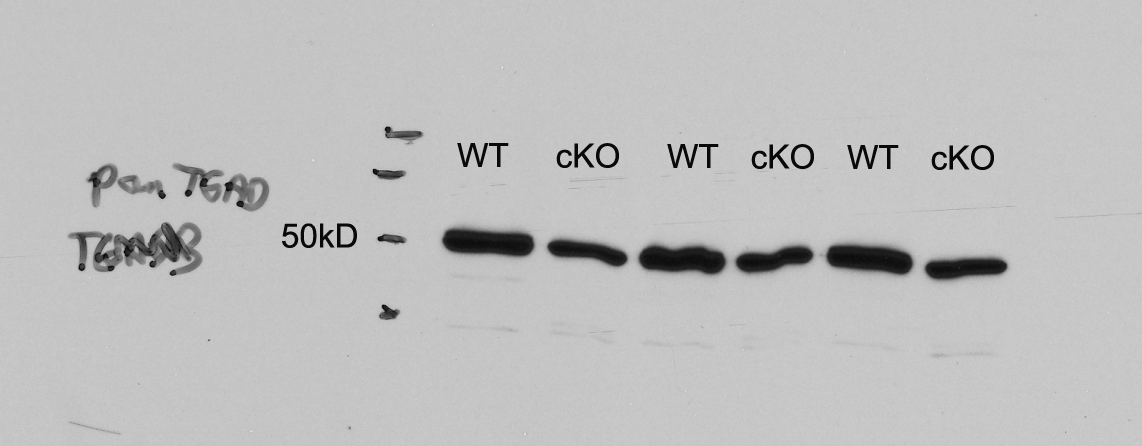

Supplement: Figure 1—source data 1. [file elife-87394-fig1-data1.zip › Fig 1 source data 1/Fig 1B blots and prizm files for graphs/panTEAD/uncropped 2 labeled.tif]

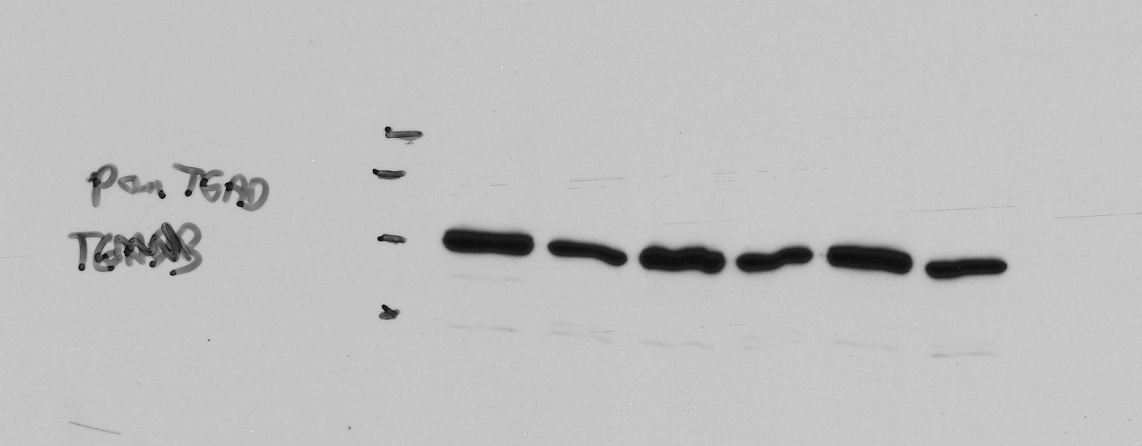

Supplement: Figure 1—source data 1. [file elife-87394-fig1-data1.zip › Fig 1 source data 1/Fig 1B blots and prizm files for graphs/panTEAD/uncropped 2.tif]

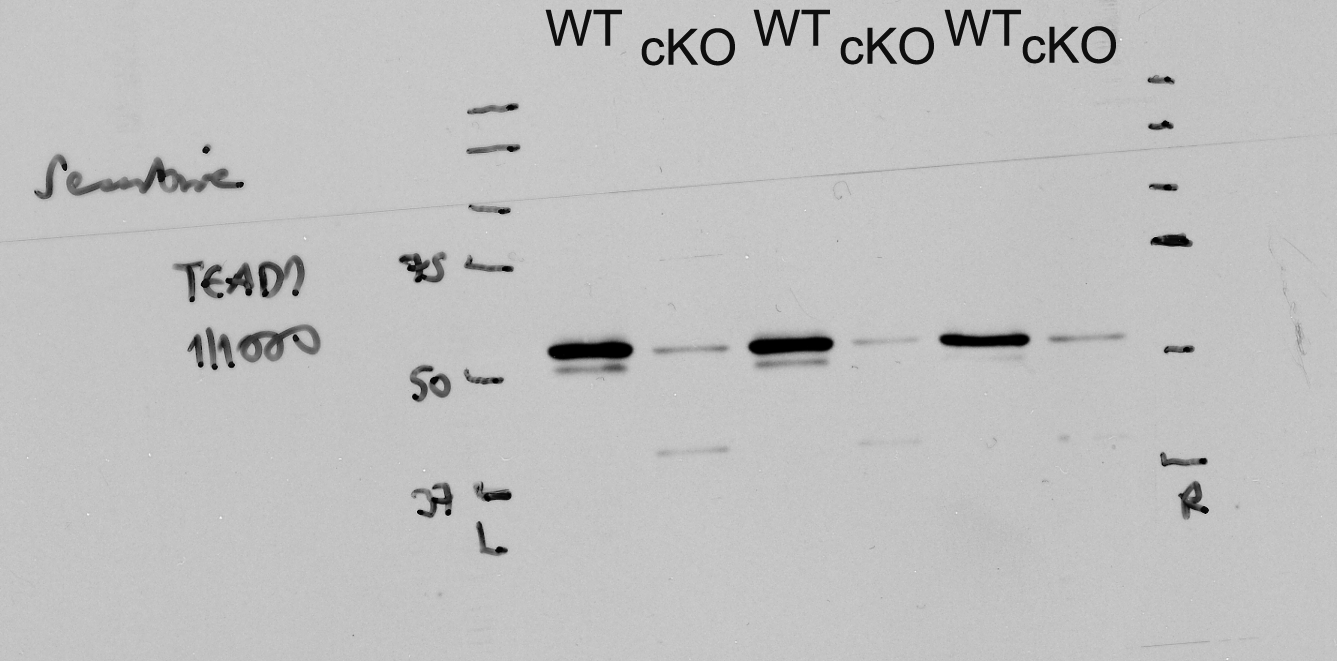

Supplement: Figure 1—source data 1. [file elife-87394-fig1-data1.zip › Fig 1 source data 1/Fig 1B blots and prizm files for graphs/TEAD1/uncropped labeled.tif]

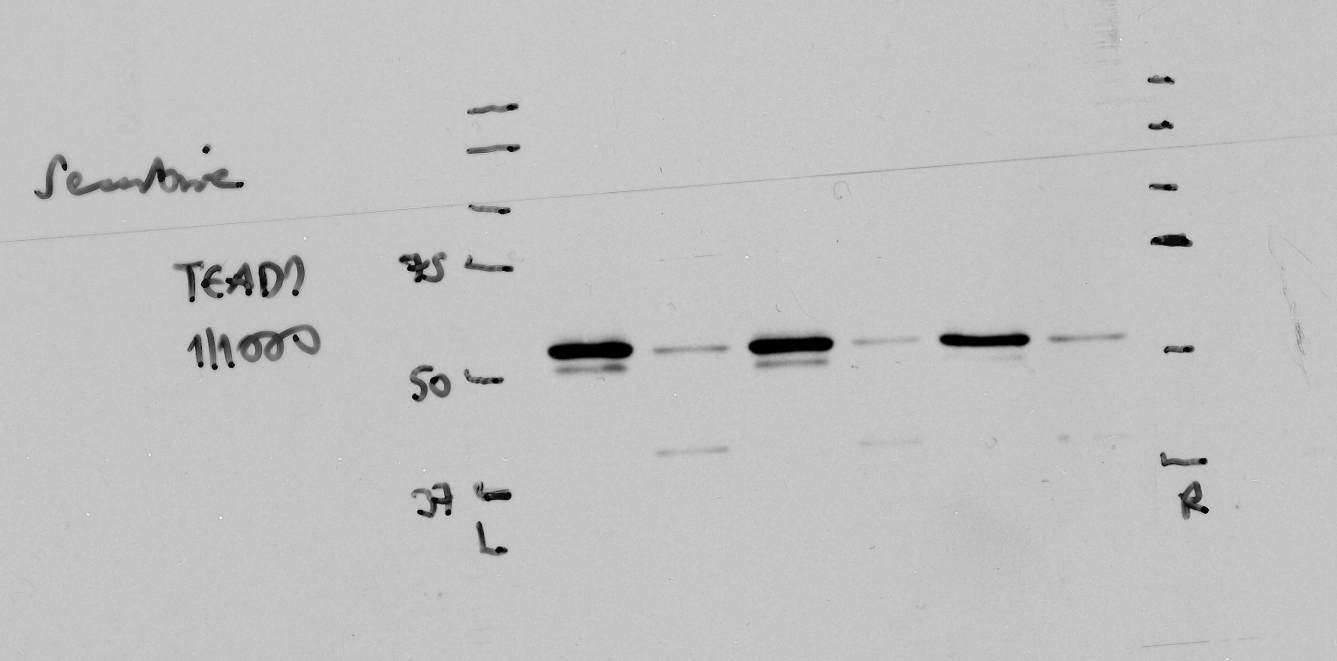

Supplement: Figure 1—source data 1. [file elife-87394-fig1-data1.zip › Fig 1 source data 1/Fig 1B blots and prizm files for graphs/TEAD1/uncropped.tif]

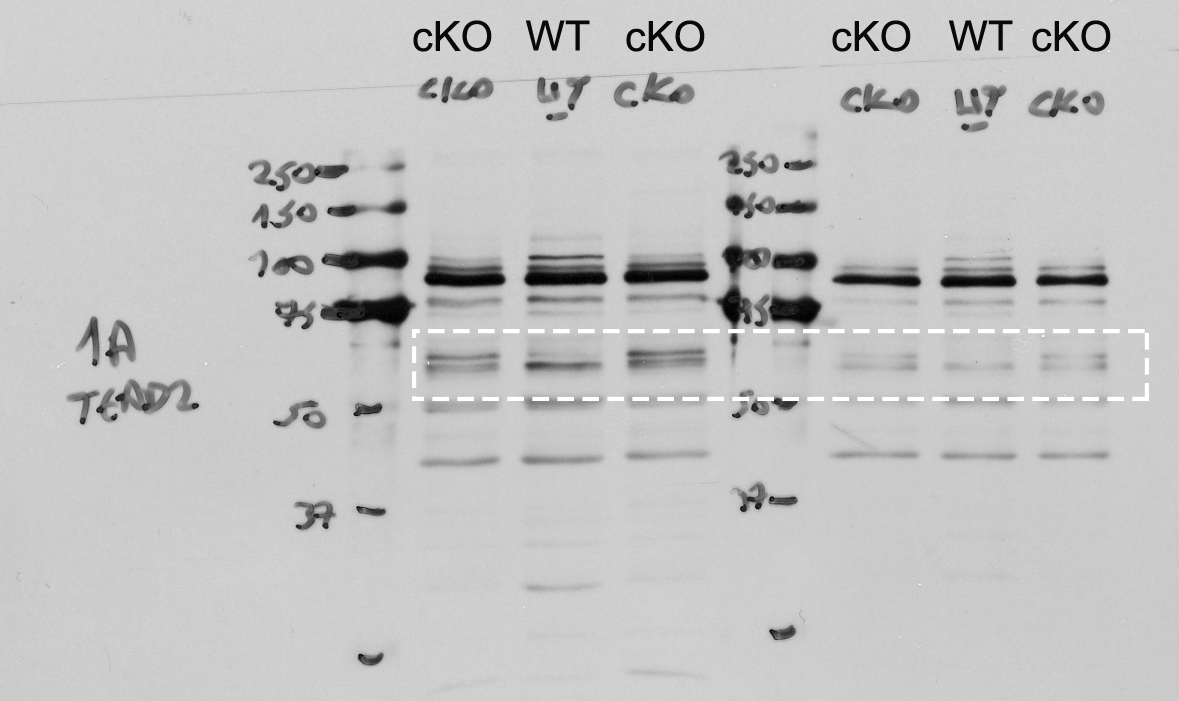

Supplement: Figure 1—source data 1. [file elife-87394-fig1-data1.zip › Fig 1 source data 1/Fig 1B blots and prizm files for graphs/TEAD2/uncropped 1 labeled.tif]

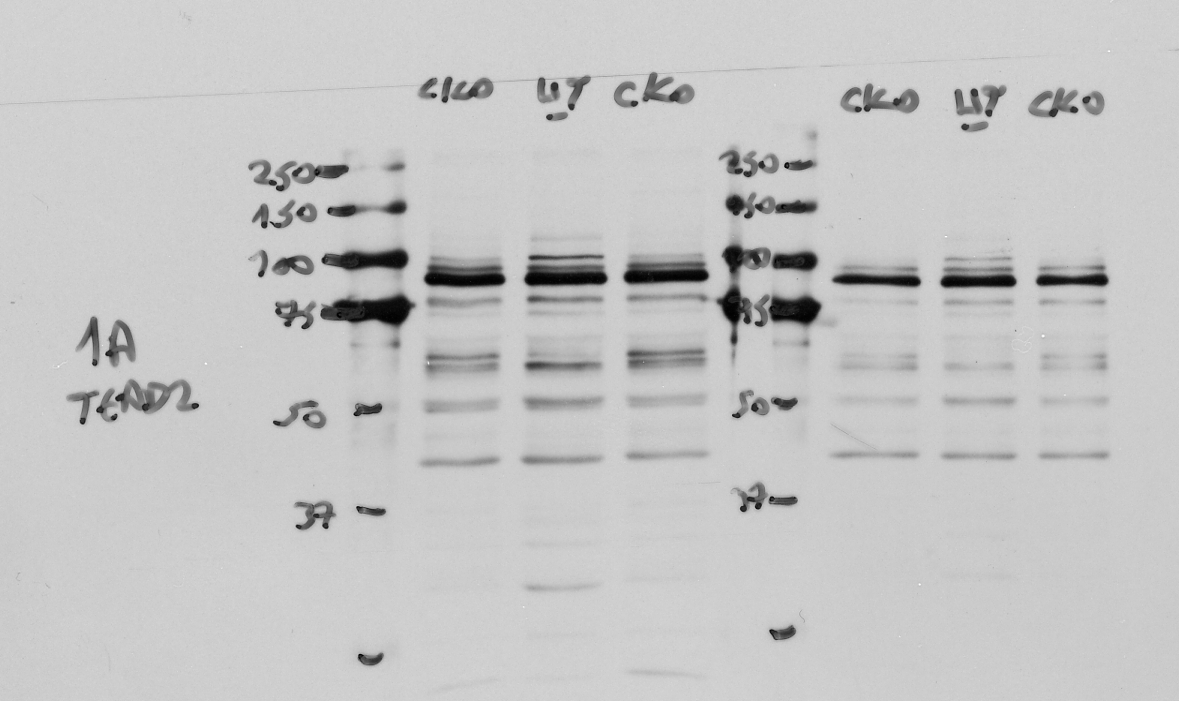

Supplement: Figure 1—source data 1. [file elife-87394-fig1-data1.zip › Fig 1 source data 1/Fig 1B blots and prizm files for graphs/TEAD2/uncropped 1.tif]

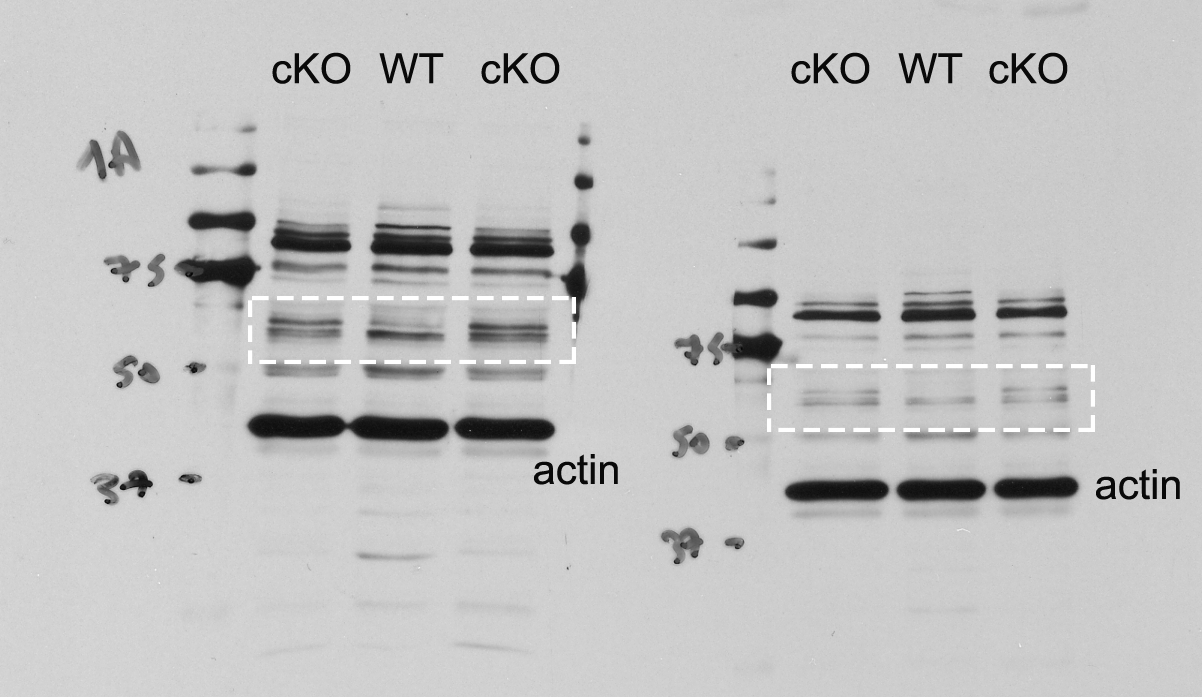

Supplement: Figure 1—source data 1. [file elife-87394-fig1-data1.zip › Fig 1 source data 1/Fig 1B blots and prizm files for graphs/TEAD2/uncropped 2 labeled.tif]

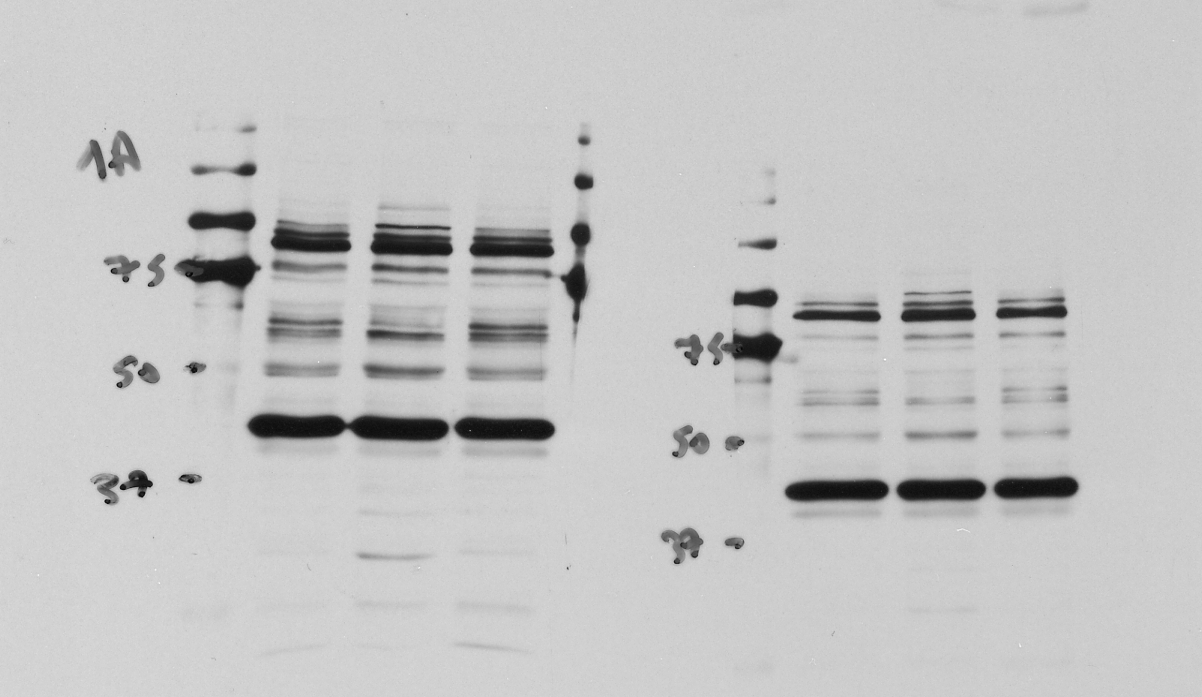

Supplement: Figure 1—source data 1. [file elife-87394-fig1-data1.zip › Fig 1 source data 1/Fig 1B blots and prizm files for graphs/TEAD2/uncropped 2.tif]

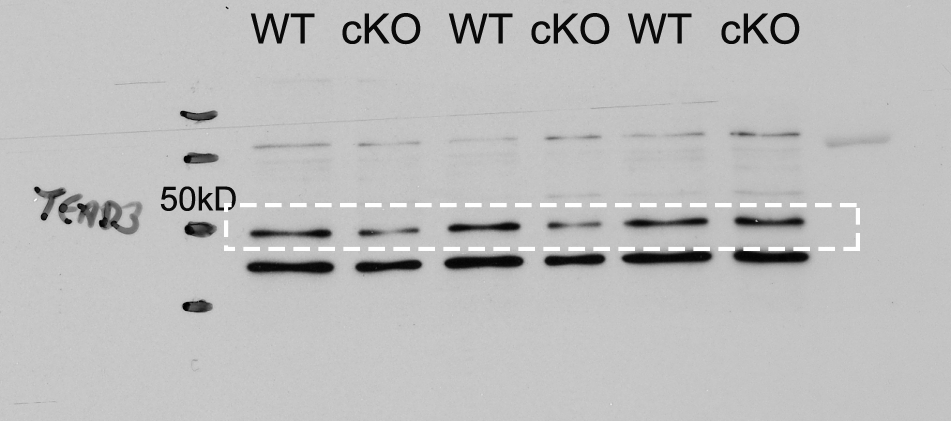

Supplement: Figure 1—source data 1. [file elife-87394-fig1-data1.zip › Fig 1 source data 1/Fig 1B blots and prizm files for graphs/TEAD3/uncropped labeled.tif]

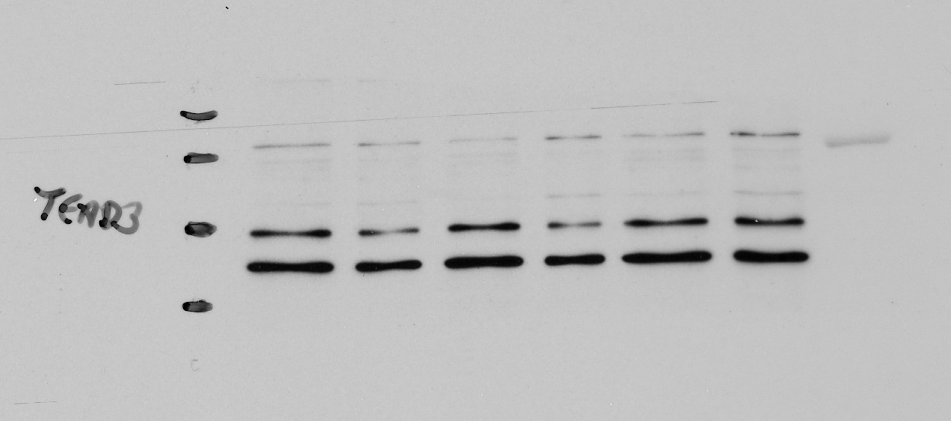

Supplement: Figure 1—source data 1. [file elife-87394-fig1-data1.zip › Fig 1 source data 1/Fig 1B blots and prizm files for graphs/TEAD3/uncropped.tif]

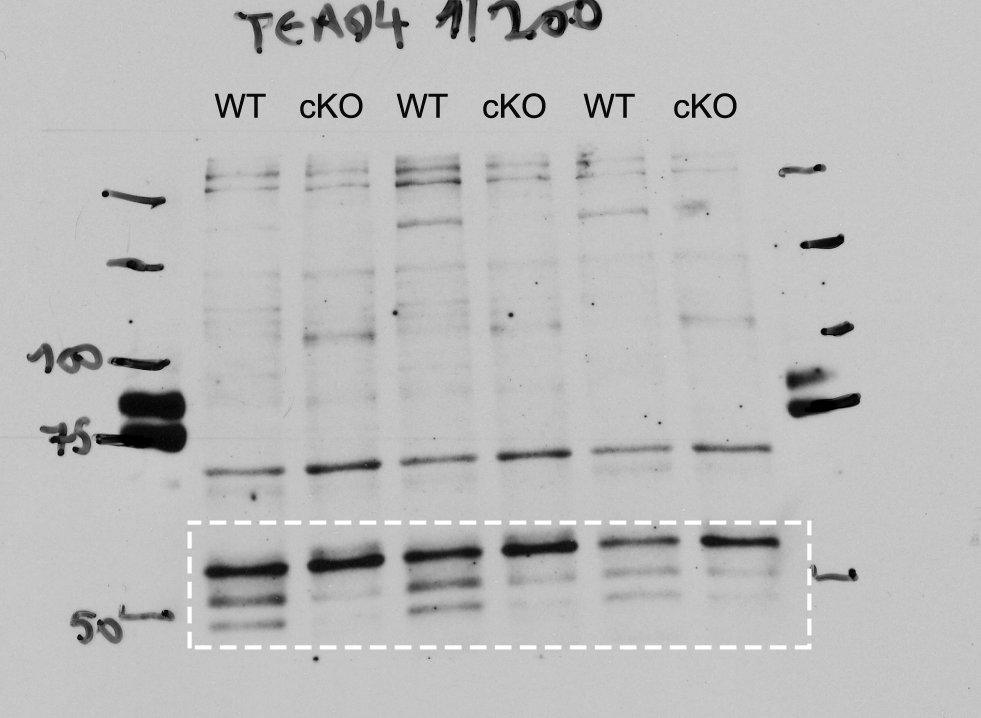

Supplement: Figure 1—source data 1. [file elife-87394-fig1-data1.zip › Fig 1 source data 1/Fig 1B blots and prizm files for graphs/TEAD4/uncropped labeled.tif]

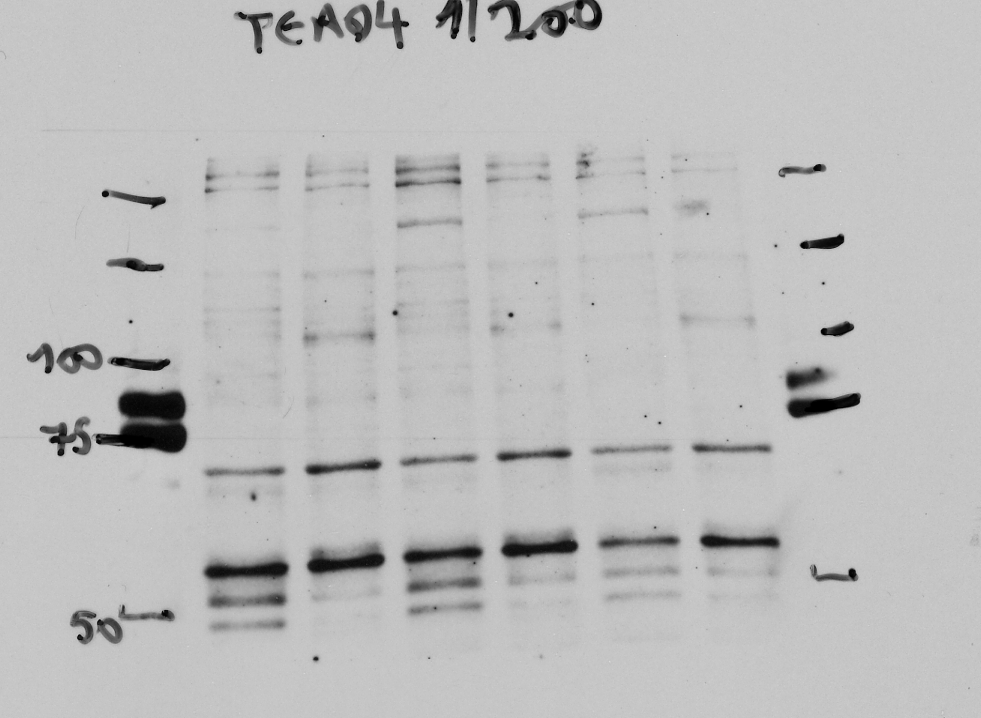

Supplement: Figure 1—source data 1. [file elife-87394-fig1-data1.zip › Fig 1 source data 1/Fig 1B blots and prizm files for graphs/TEAD4/uncropped.tif]

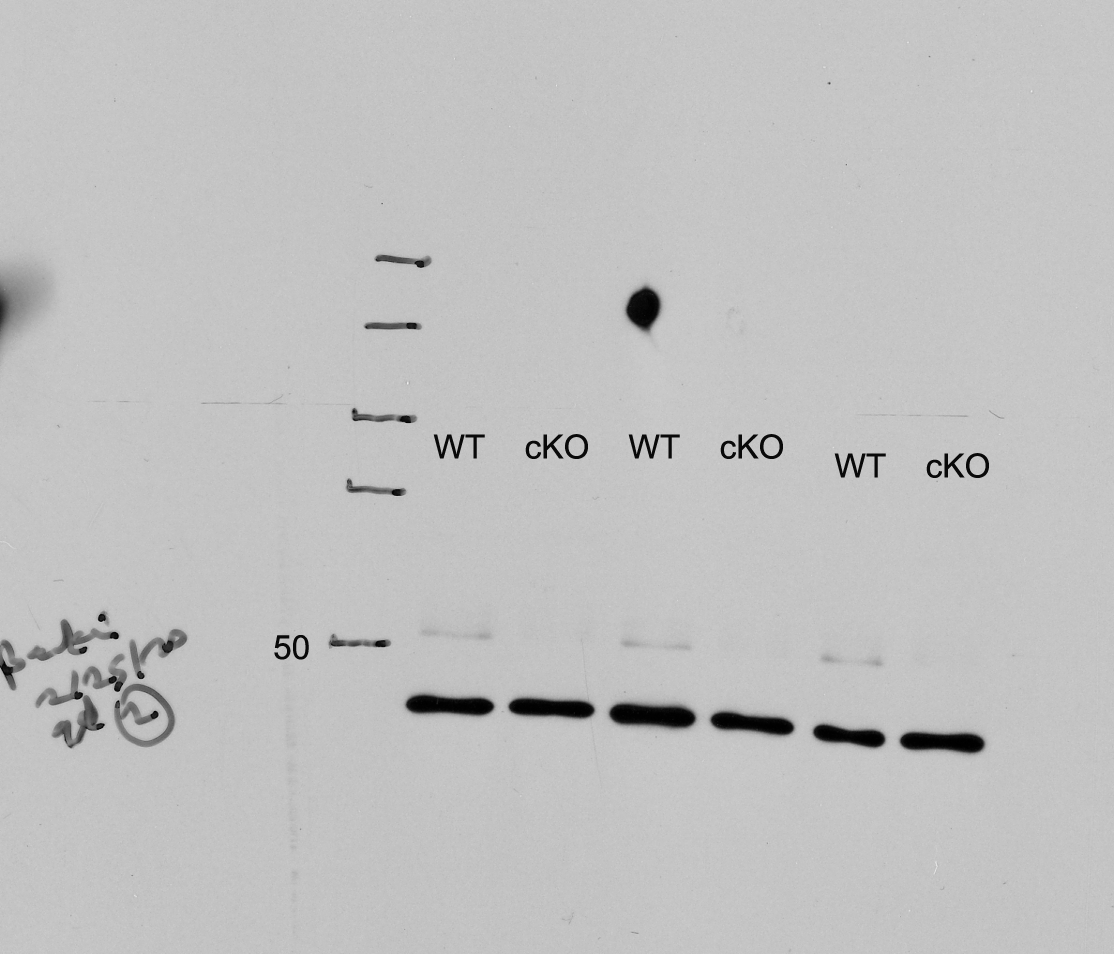

Supplement: Figure 1—source data 1. [file elife-87394-fig1-data1.zip › Fig 1 source data 1/Fig 1C blots and prism files/Beta actin/uncropped 1 labeled.tif]

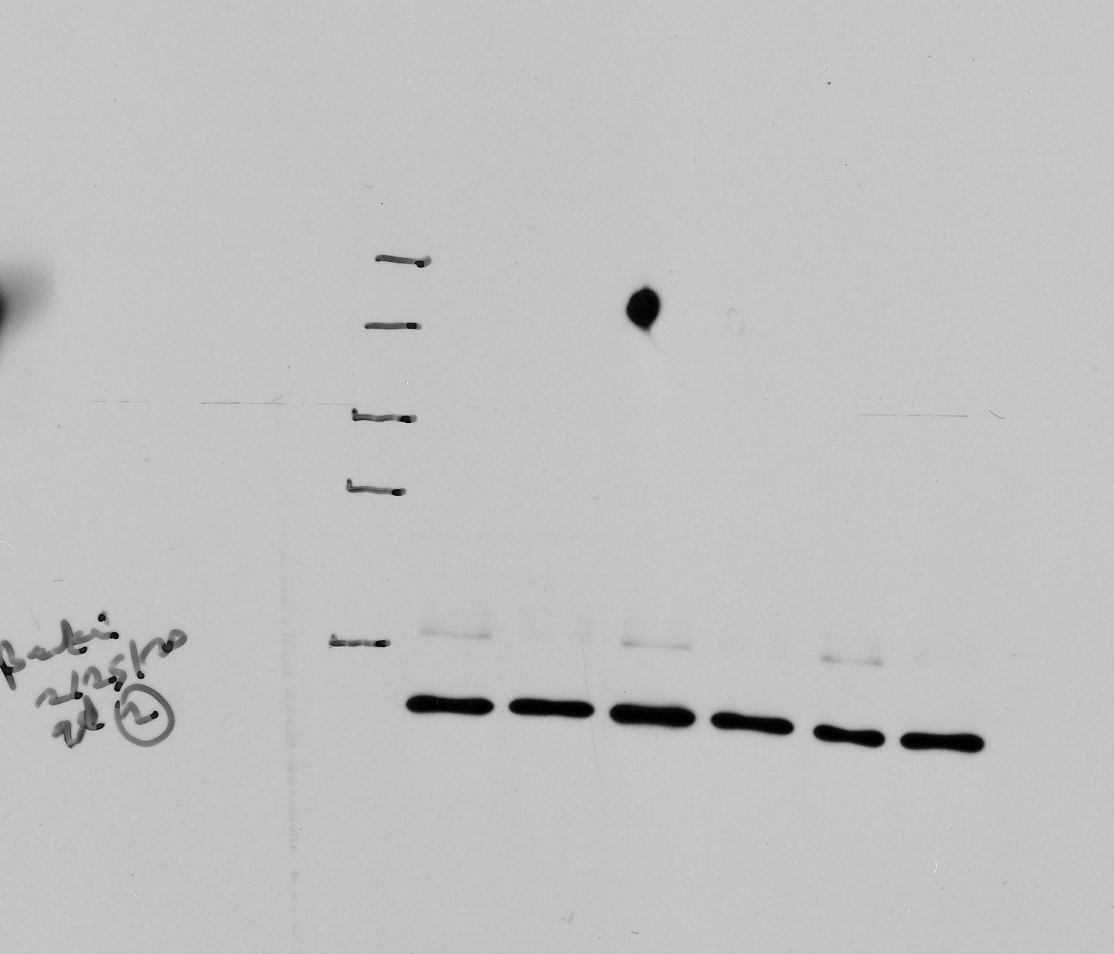

Supplement: Figure 1—source data 1. [file elife-87394-fig1-data1.zip › Fig 1 source data 1/Fig 1C blots and prism files/Beta actin/uncropped 1.tif]

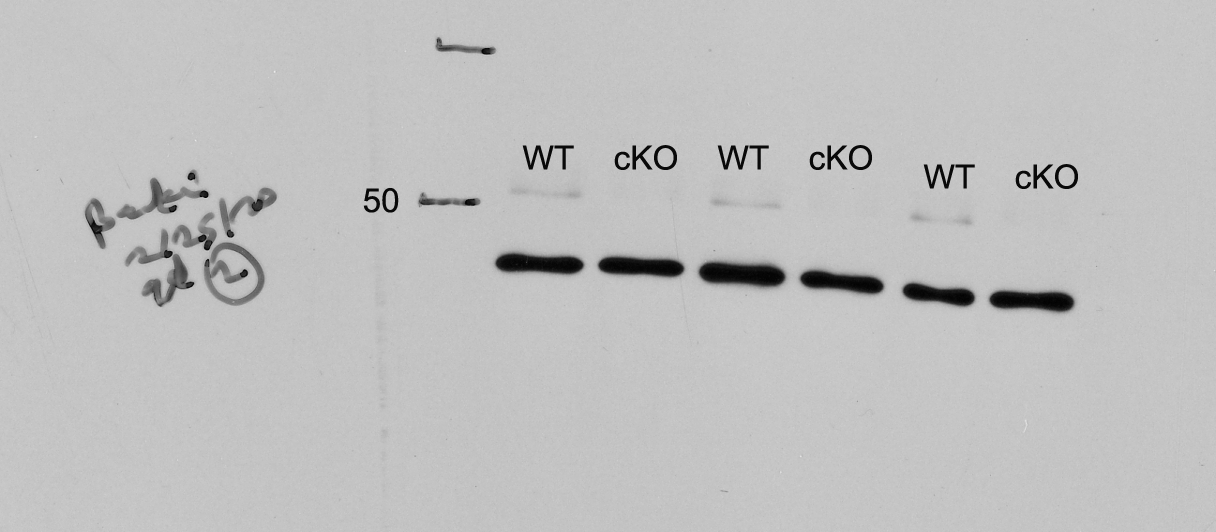

Supplement: Figure 1—source data 1. [file elife-87394-fig1-data1.zip › Fig 1 source data 1/Fig 1C blots and prism files/Beta actin/uncropped 2 labeled.tif]

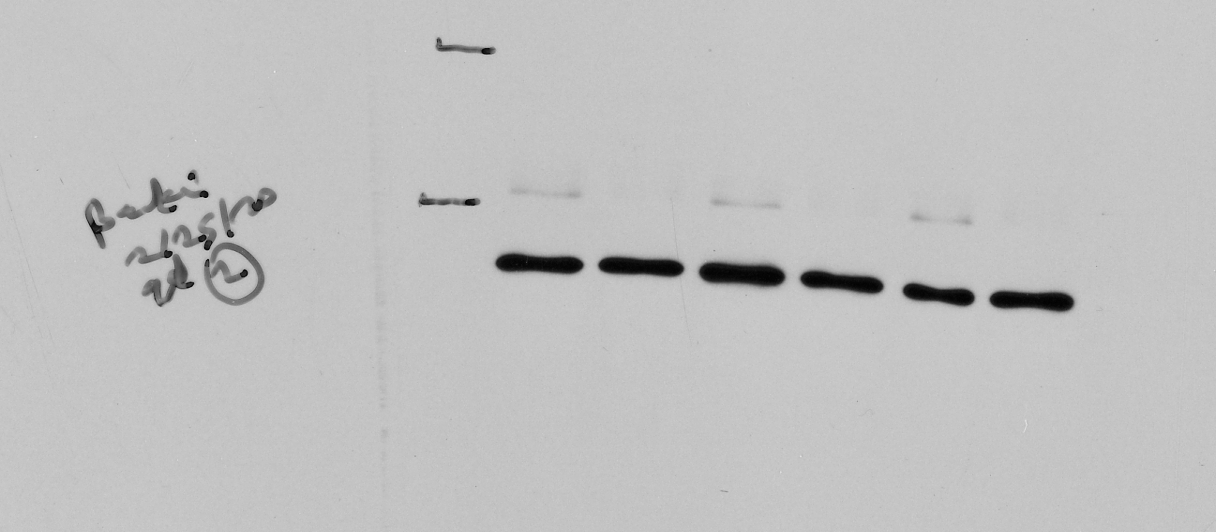

Supplement: Figure 1—source data 1. [file elife-87394-fig1-data1.zip › Fig 1 source data 1/Fig 1C blots and prism files/Beta actin/uncropped 2.tif]

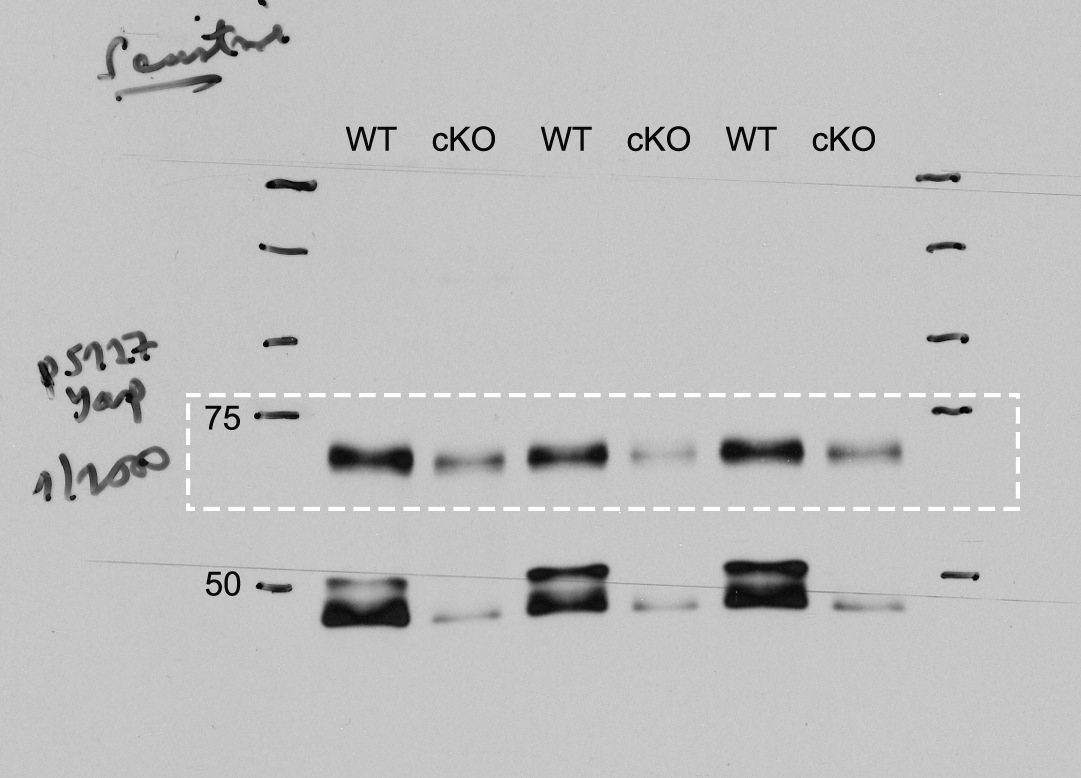

Supplement: Figure 1—source data 1. [file elife-87394-fig1-data1.zip › Fig 1 source data 1/Fig 1C blots and prism files/pS127-YAP/uncropped labeled.tif]

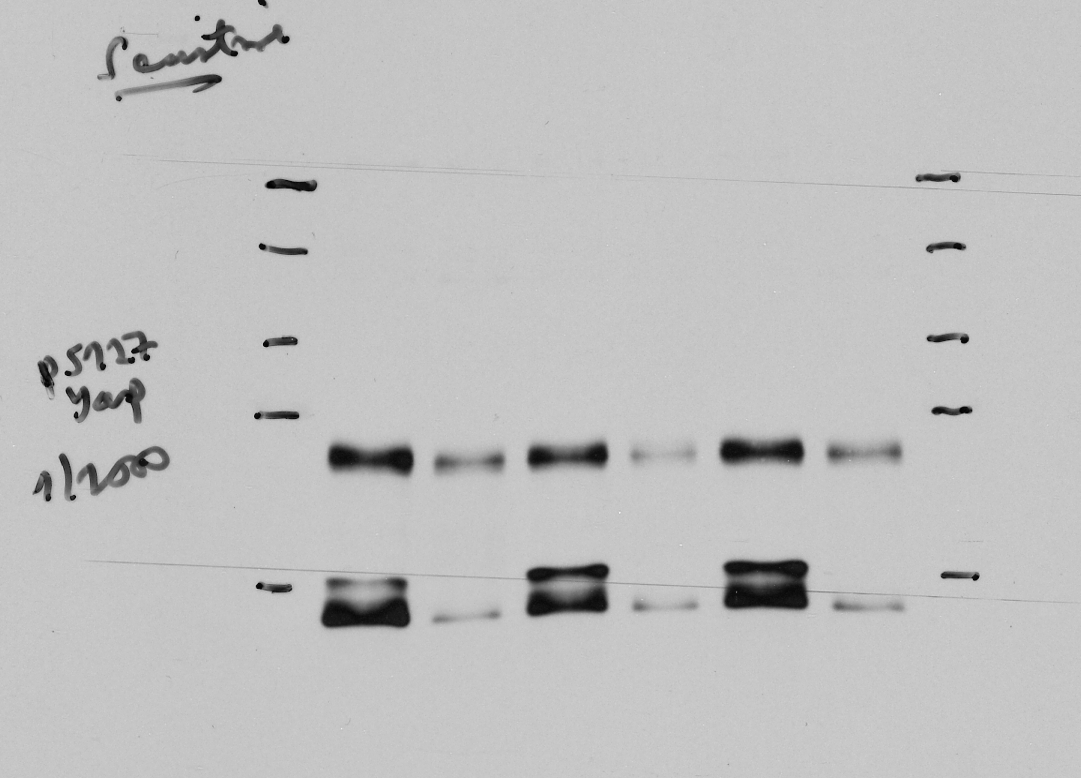

Supplement: Figure 1—source data 1. [file elife-87394-fig1-data1.zip › Fig 1 source data 1/Fig 1C blots and prism files/pS127-YAP/uncropped.tif]

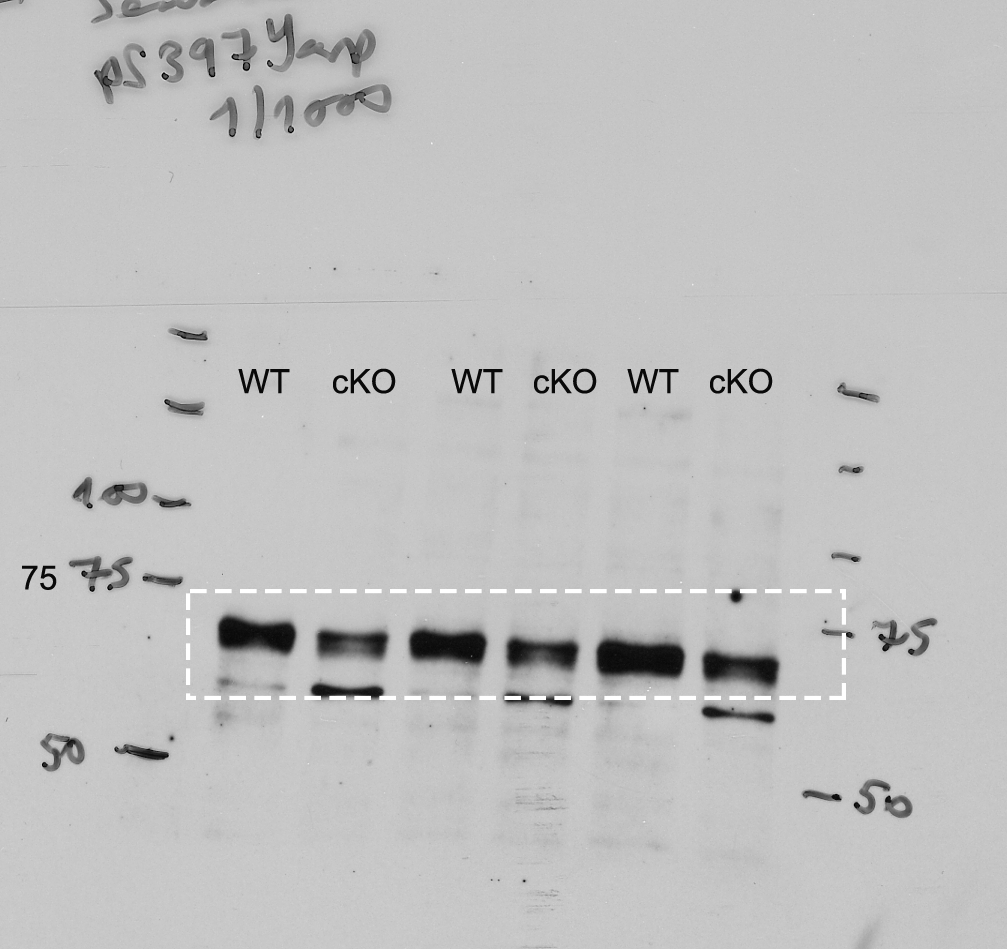

Supplement: Figure 1—source data 1. [file elife-87394-fig1-data1.zip › Fig 1 source data 1/Fig 1C blots and prism files/pS397-YAP/uncropped 1 labeled.tif]

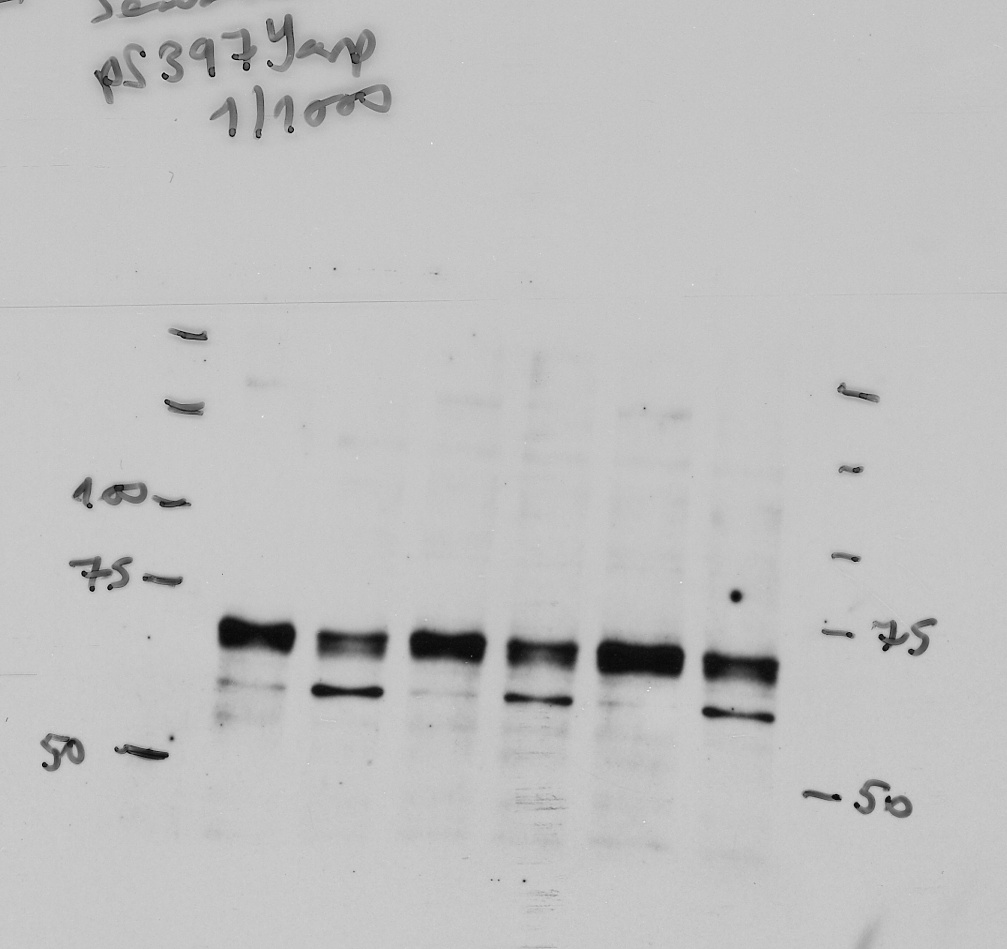

Supplement: Figure 1—source data 1. [file elife-87394-fig1-data1.zip › Fig 1 source data 1/Fig 1C blots and prism files/pS397-YAP/uncropped 1.tif]

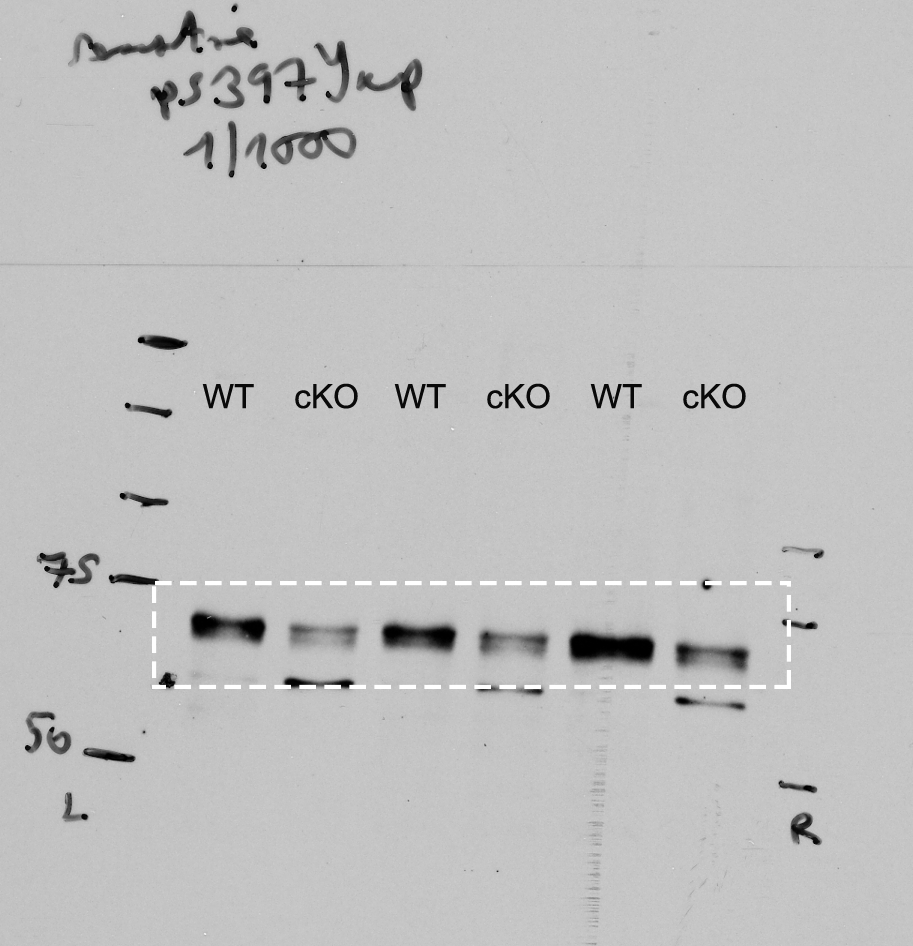

Supplement: Figure 1—source data 1. [file elife-87394-fig1-data1.zip › Fig 1 source data 1/Fig 1C blots and prism files/pS397-YAP/uncropped 2 labeled.tif]

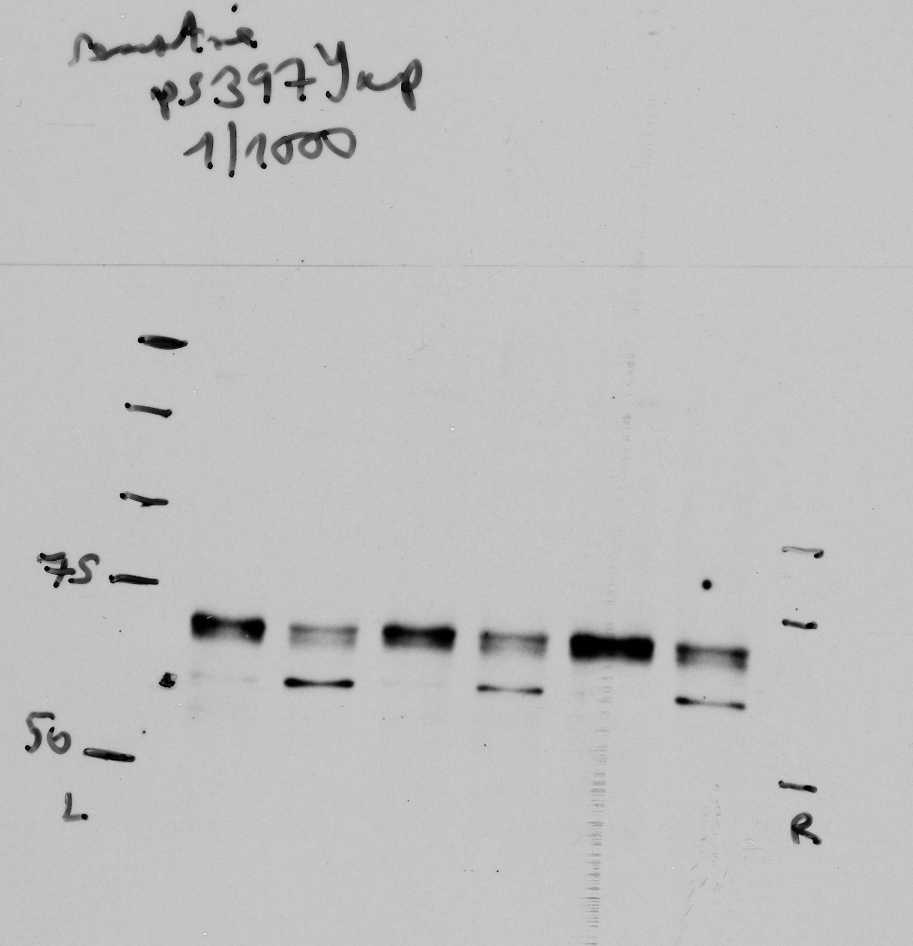

Supplement: Figure 1—source data 1. [file elife-87394-fig1-data1.zip › Fig 1 source data 1/Fig 1C blots and prism files/pS397-YAP/uncropped 2.tif]

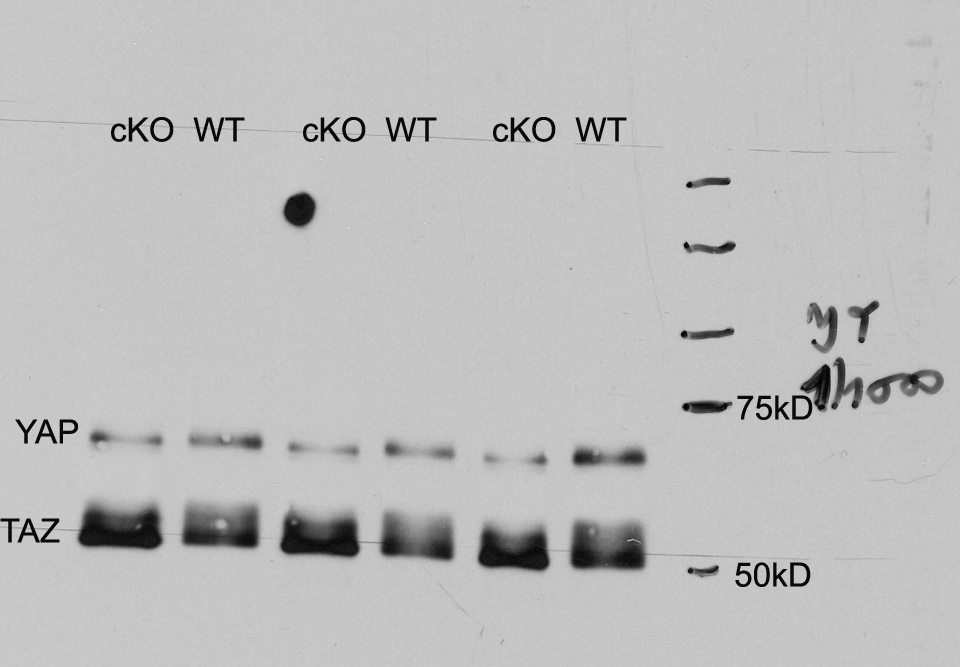

Supplement: Figure 1—source data 1. [file elife-87394-fig1-data1.zip › Fig 1 source data 1/Fig 1C blots and prism files/Yap and Taz/uncropped 1 labeled.tif]

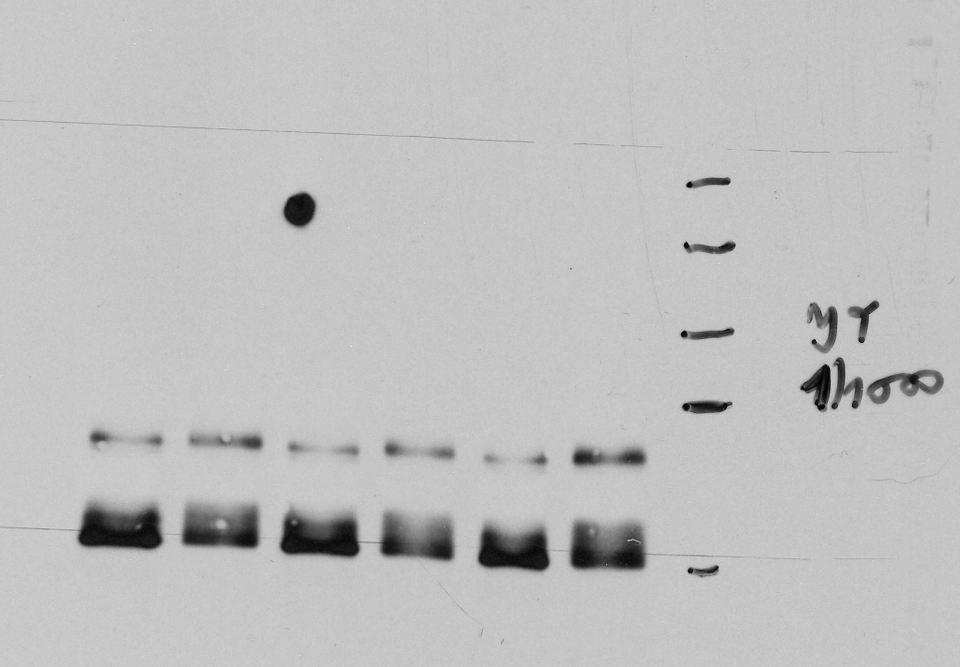

Supplement: Figure 1—source data 1. [file elife-87394-fig1-data1.zip › Fig 1 source data 1/Fig 1C blots and prism files/Yap and Taz/uncropped 1.tif]

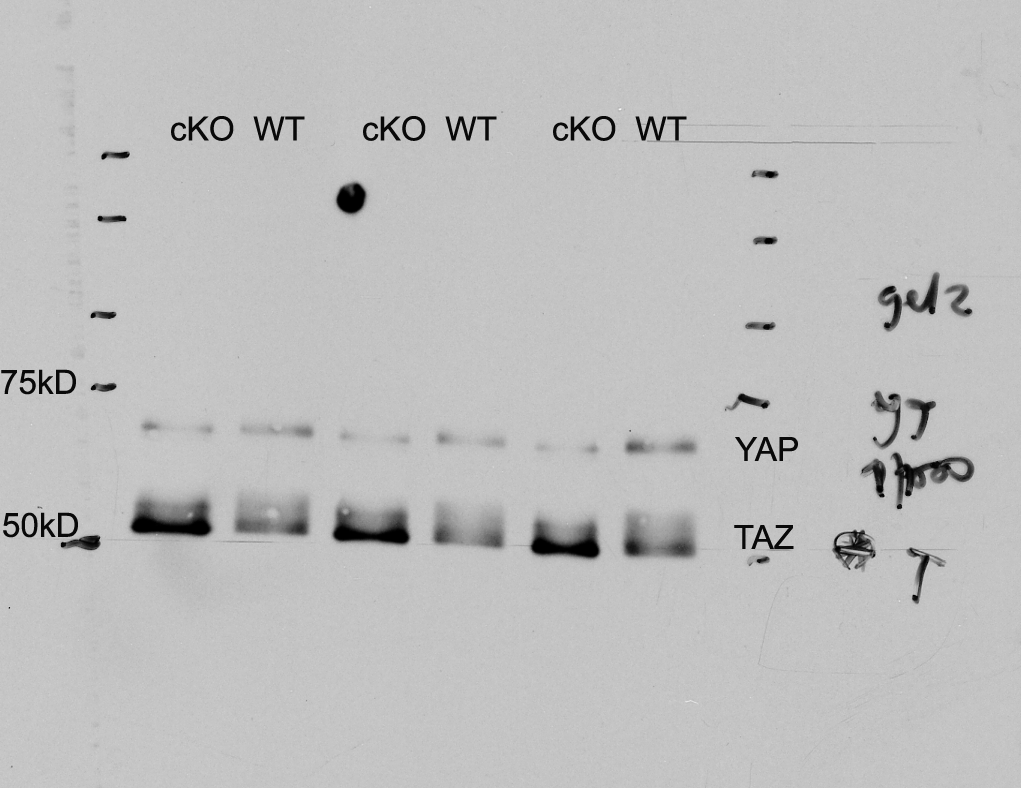

Supplement: Figure 1—source data 1. [file elife-87394-fig1-data1.zip › Fig 1 source data 1/Fig 1C blots and prism files/Yap and Taz/uncropped 2 labeled.tif]

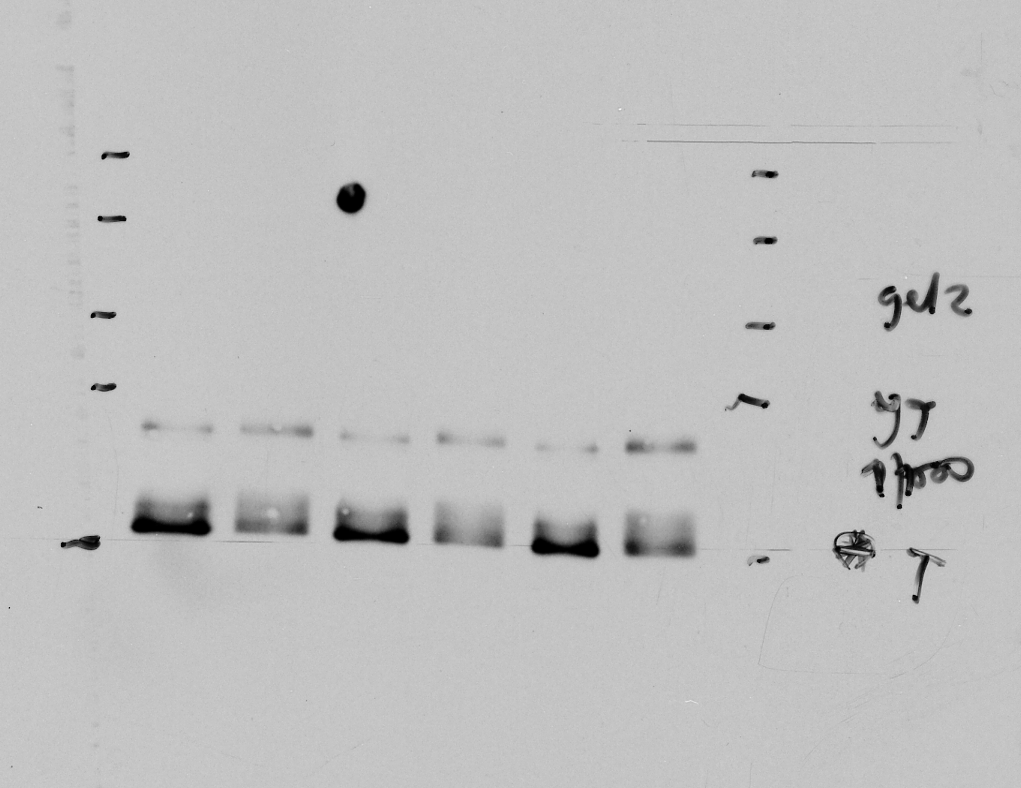

Supplement: Figure 1—source data 1. [file elife-87394-fig1-data1.zip › Fig 1 source data 1/Fig 1C blots and prism files/Yap and Taz/uncropped 2.tif]

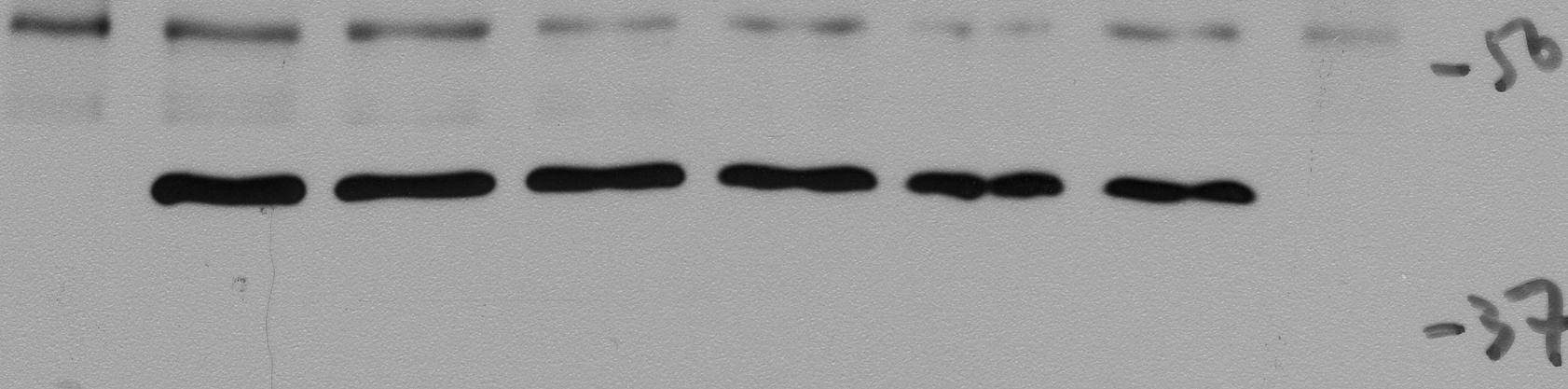

Supplement: Figure 1—figure supplement 1—source data 1. [file elife-87394-fig1-figsupp1-data1.zip › Fig 1 source data 2/uncropped b actin.tif]

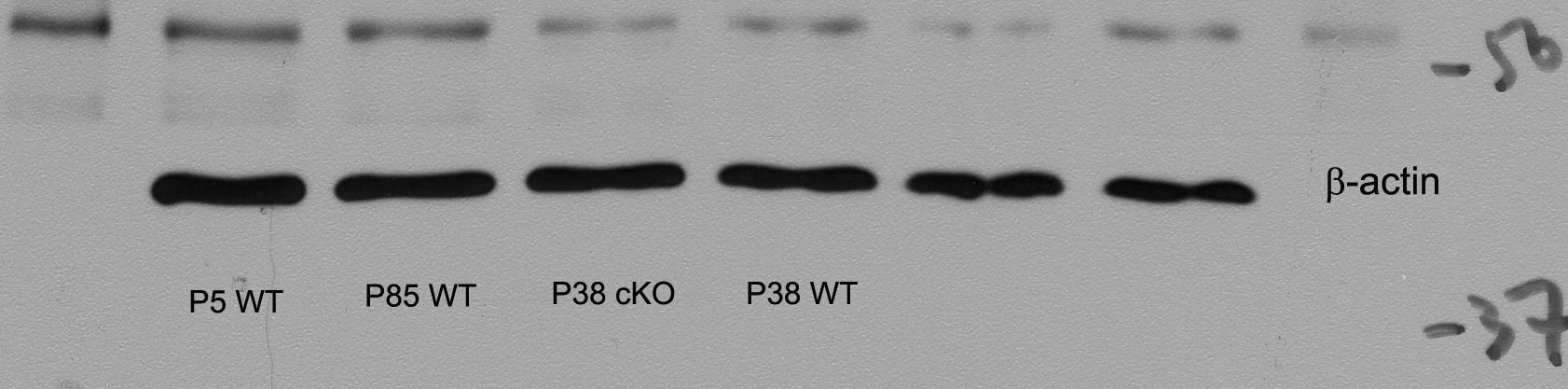

Supplement: Figure 1—figure supplement 1—source data 1. [file elife-87394-fig1-figsupp1-data1.zip › Fig 1 source data 2/uncropped labeled b actin.tif]

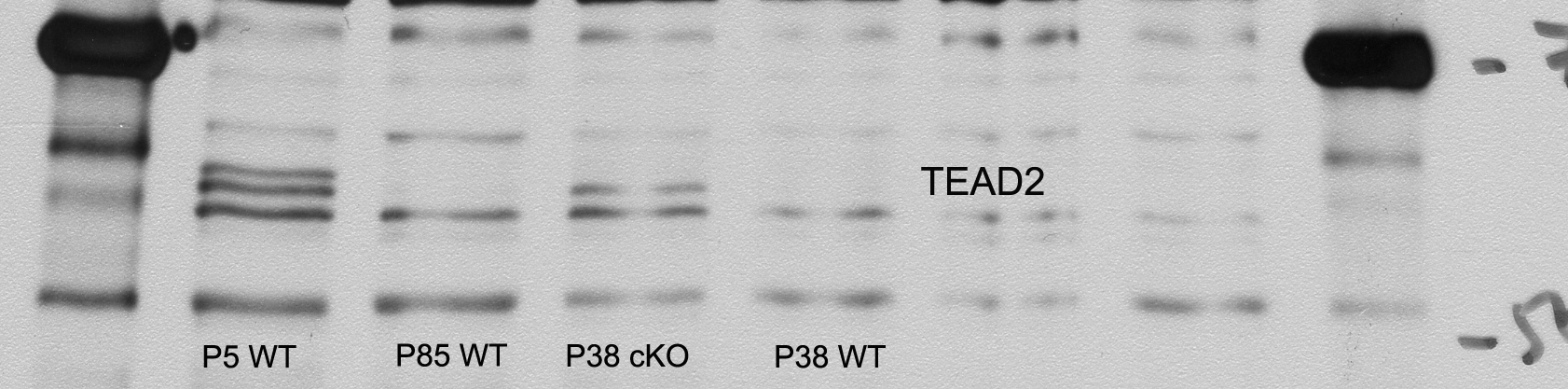

Supplement: Figure 1—figure supplement 1—source data 1. [file elife-87394-fig1-figsupp1-data1.zip › Fig 1 source data 2/uncropped labeled TEAD2.tif]

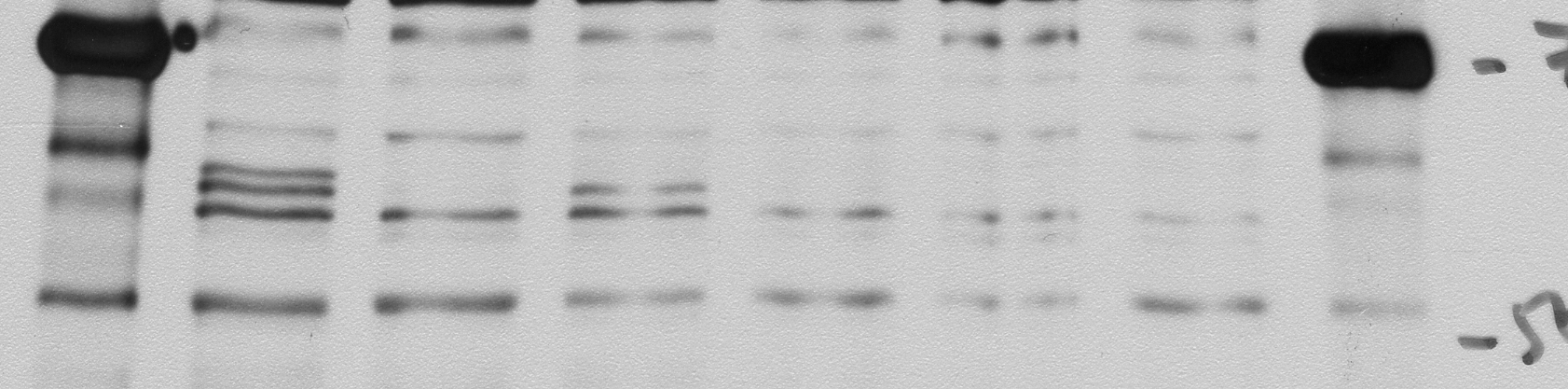

Supplement: Figure 1—figure supplement 1—source data 1. [file elife-87394-fig1-figsupp1-data1.zip › Fig 1 source data 2/uncropped TEAD2.tif]

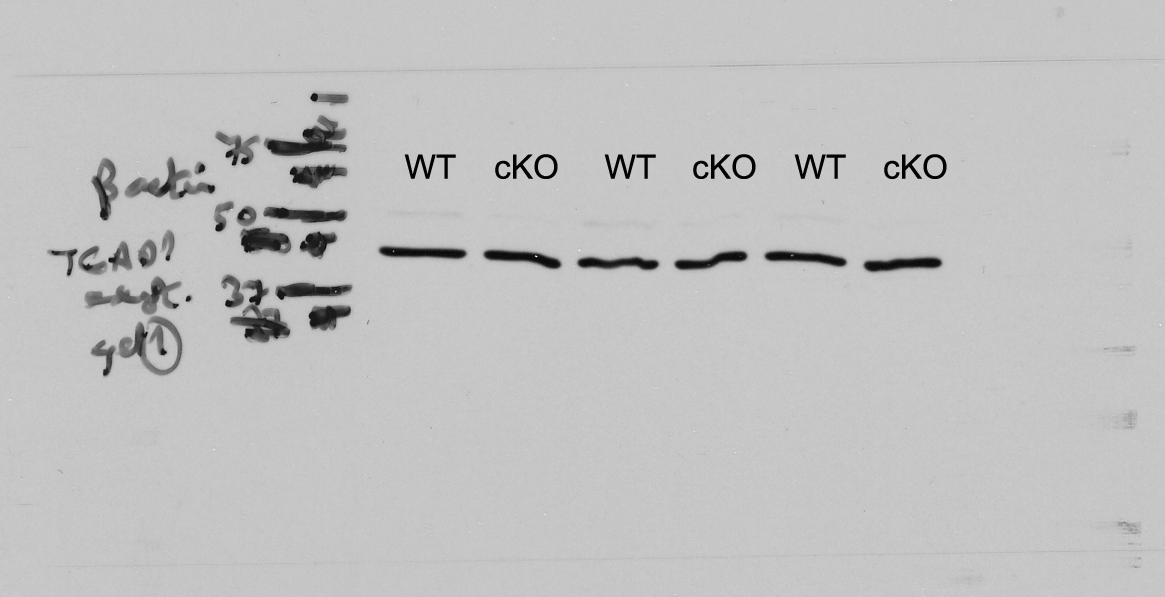

Supplement: Figure 4—source data 1. [file elife-87394-fig4-data1.zip › Fig 4 souce data 1/Fig 4A blots and prism files for graphs/actin for MBP/uncropped labeled.tif]

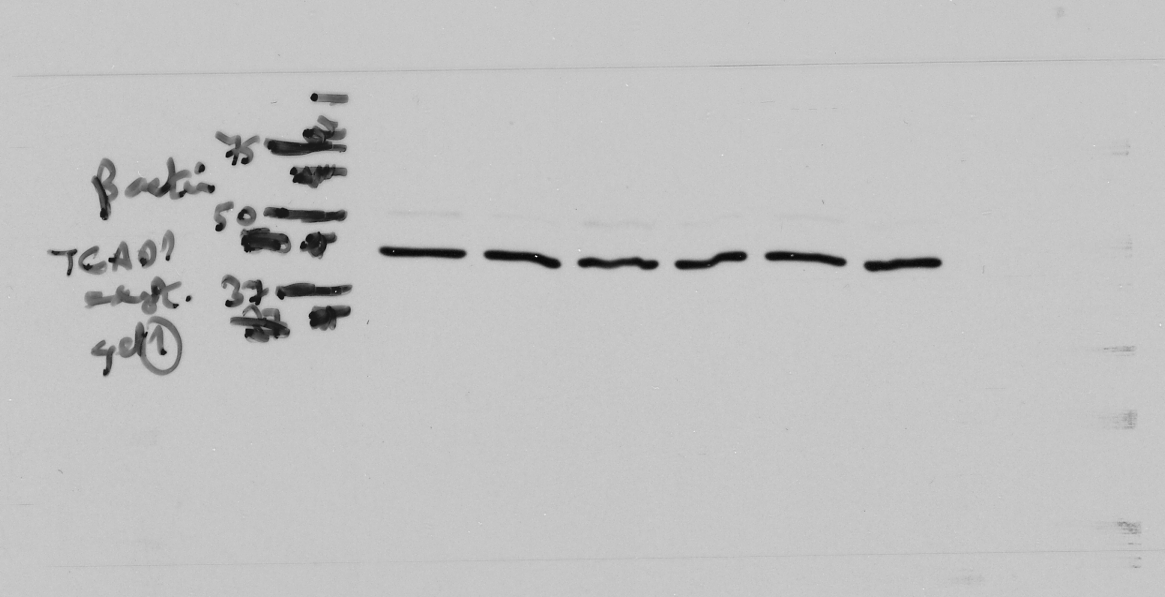

Supplement: Figure 4—source data 1. [file elife-87394-fig4-data1.zip › Fig 4 souce data 1/Fig 4A blots and prism files for graphs/actin for MBP/uncropped.tif]

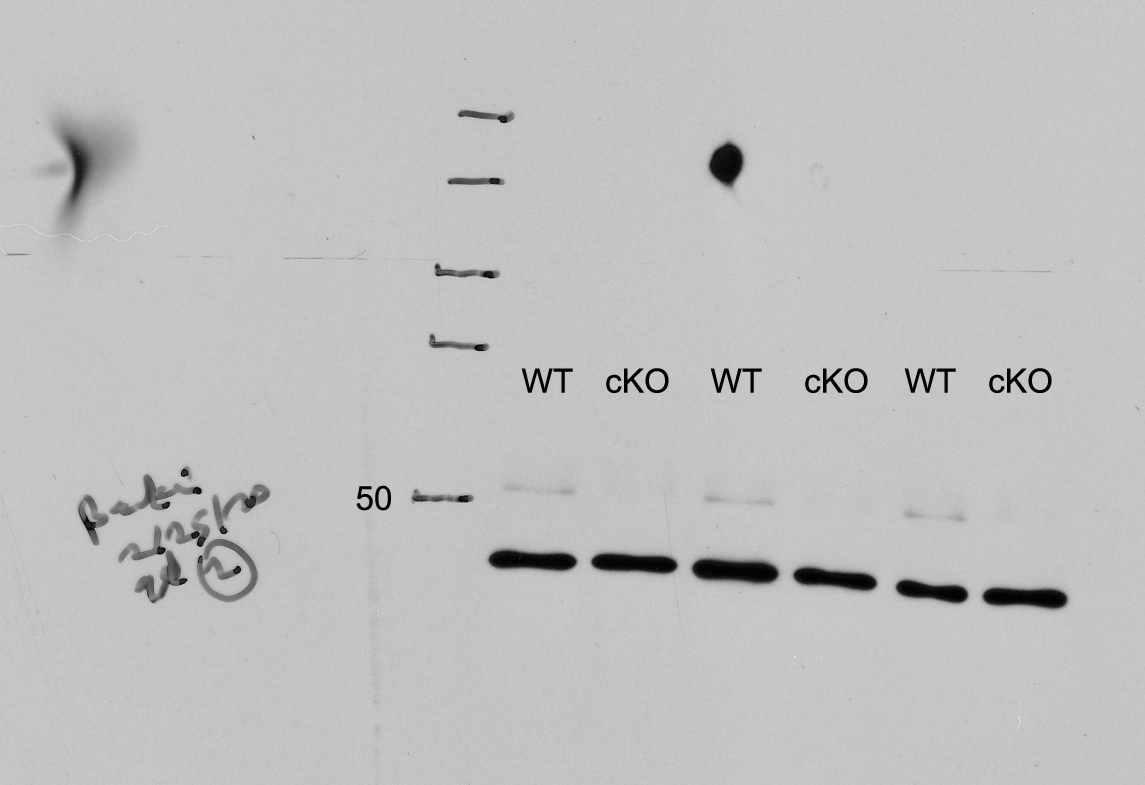

Supplement: Figure 4—source data 1. [file elife-87394-fig4-data1.zip › Fig 4 souce data 1/Fig 4A blots and prism files for graphs/actin for MPZ/uncropped labeled.tif]

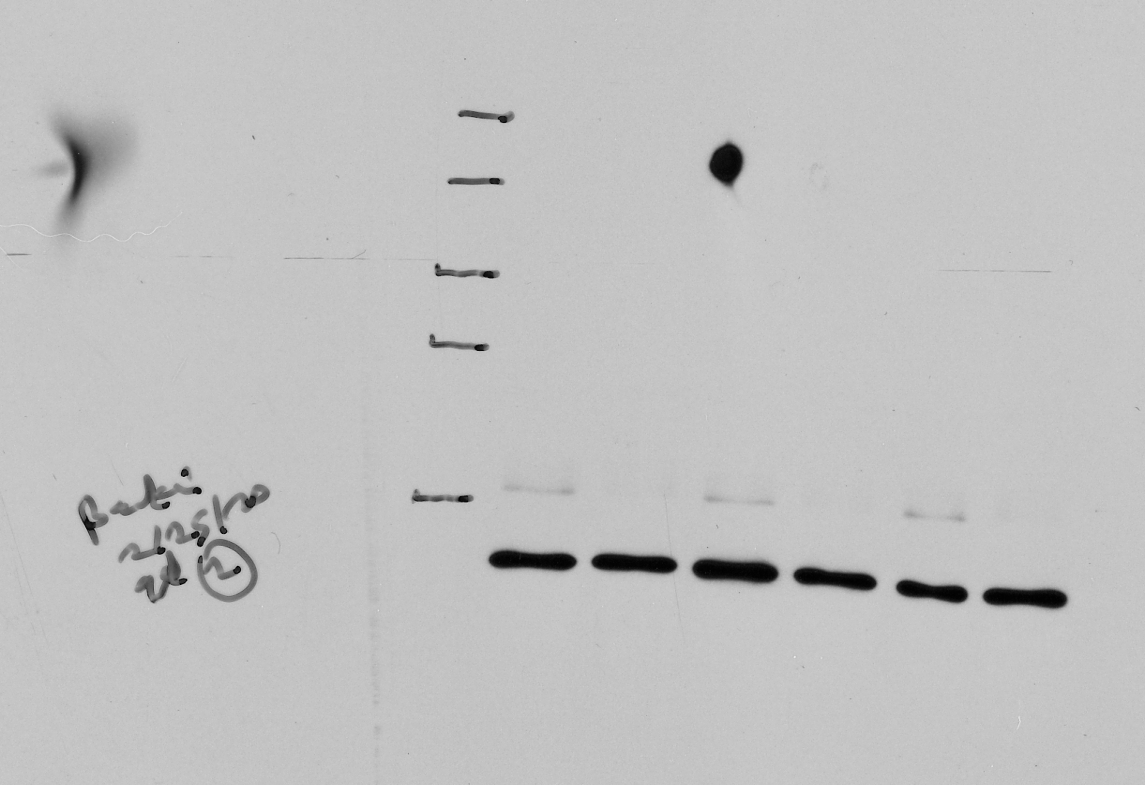

Supplement: Figure 4—source data 1. [file elife-87394-fig4-data1.zip › Fig 4 souce data 1/Fig 4A blots and prism files for graphs/actin for MPZ/uncropped.tif]

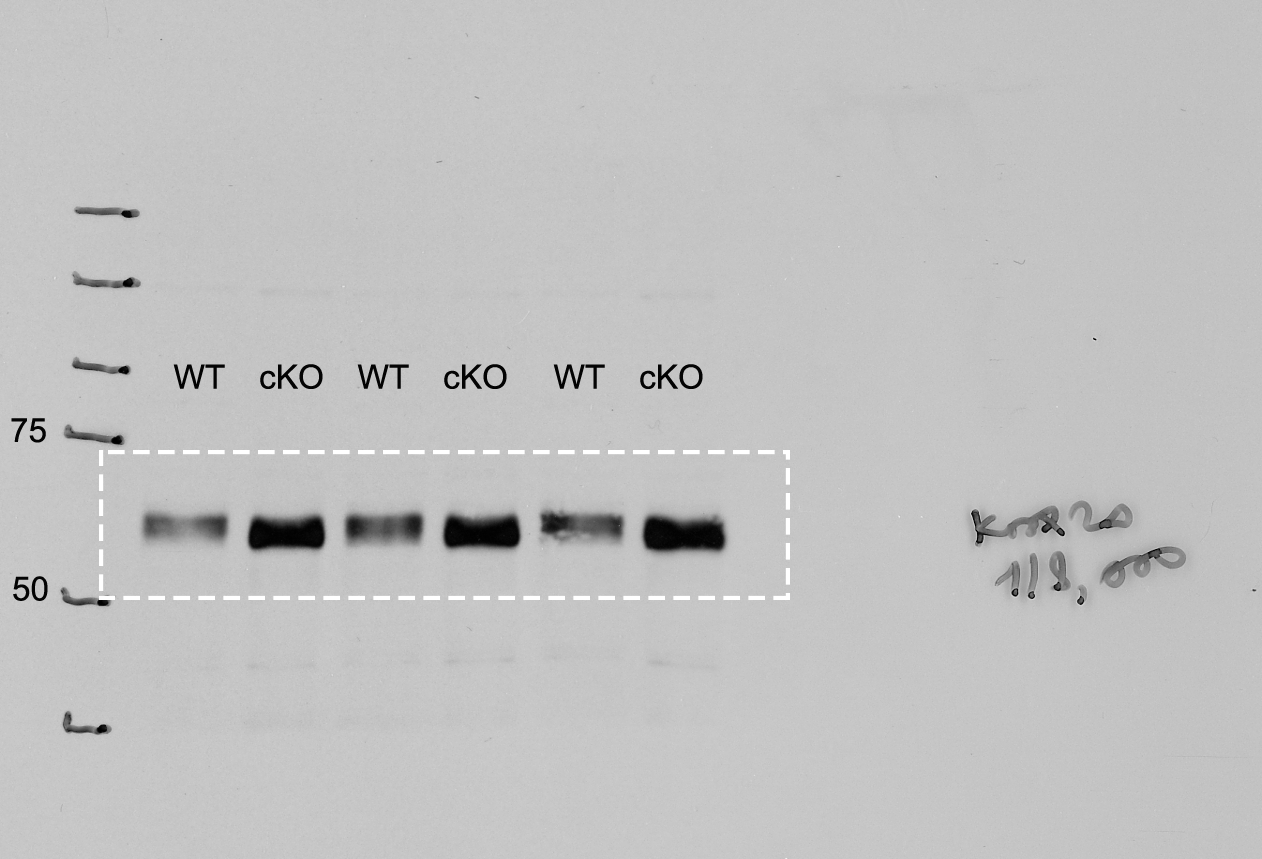

Supplement: Figure 4—source data 1. [file elife-87394-fig4-data1.zip › Fig 4 souce data 1/Fig 4A blots and prism files for graphs/Krox20/uncropped labeled.tif]

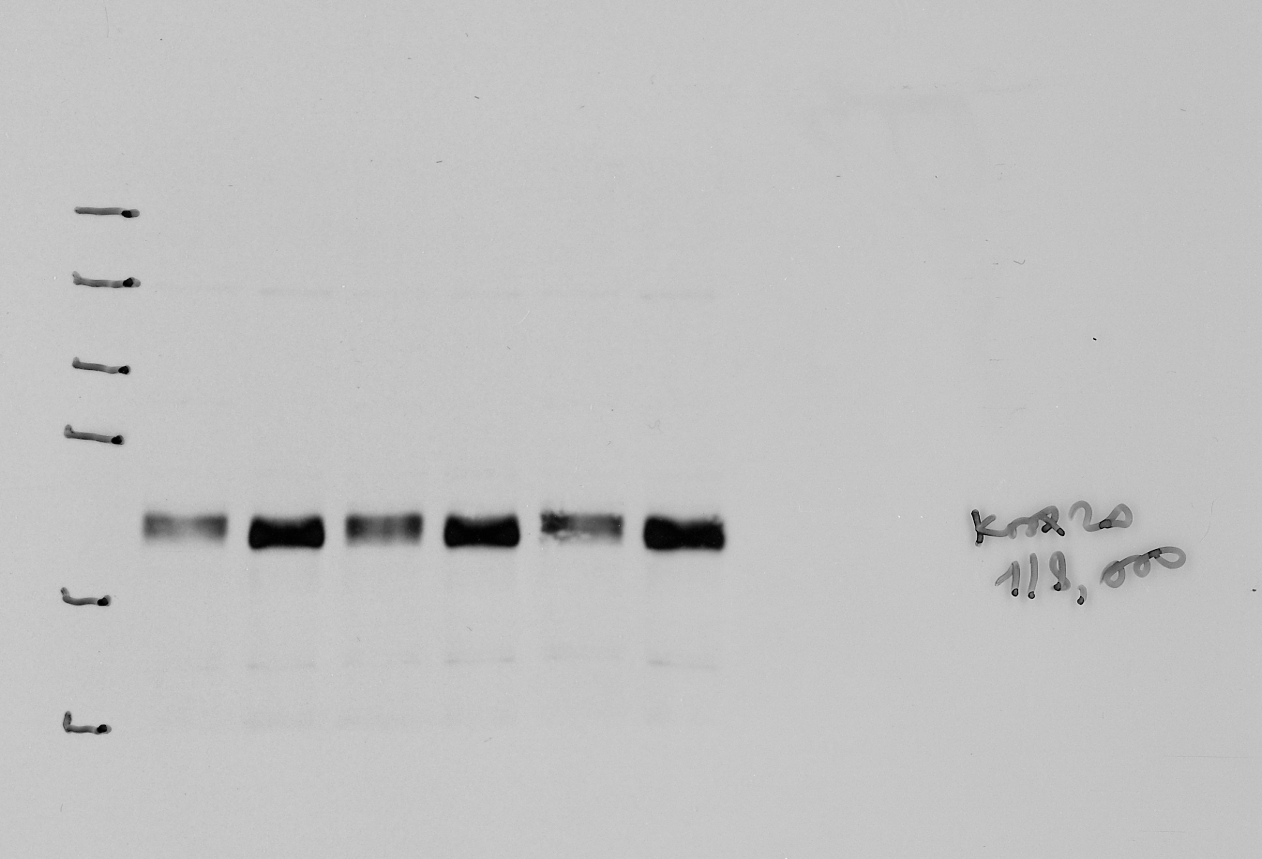

Supplement: Figure 4—source data 1. [file elife-87394-fig4-data1.zip › Fig 4 souce data 1/Fig 4A blots and prism files for graphs/Krox20/uncropped.tif]

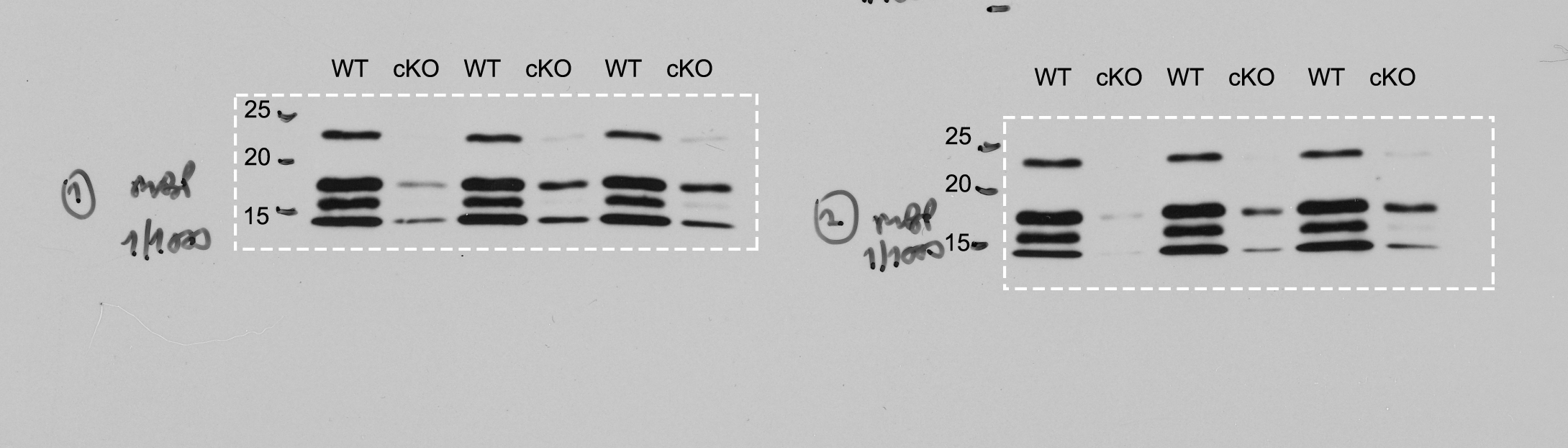

Supplement: Figure 4—source data 1. [file elife-87394-fig4-data1.zip › Fig 4 souce data 1/Fig 4A blots and prism files for graphs/MBP/uncropped labeled.tif]

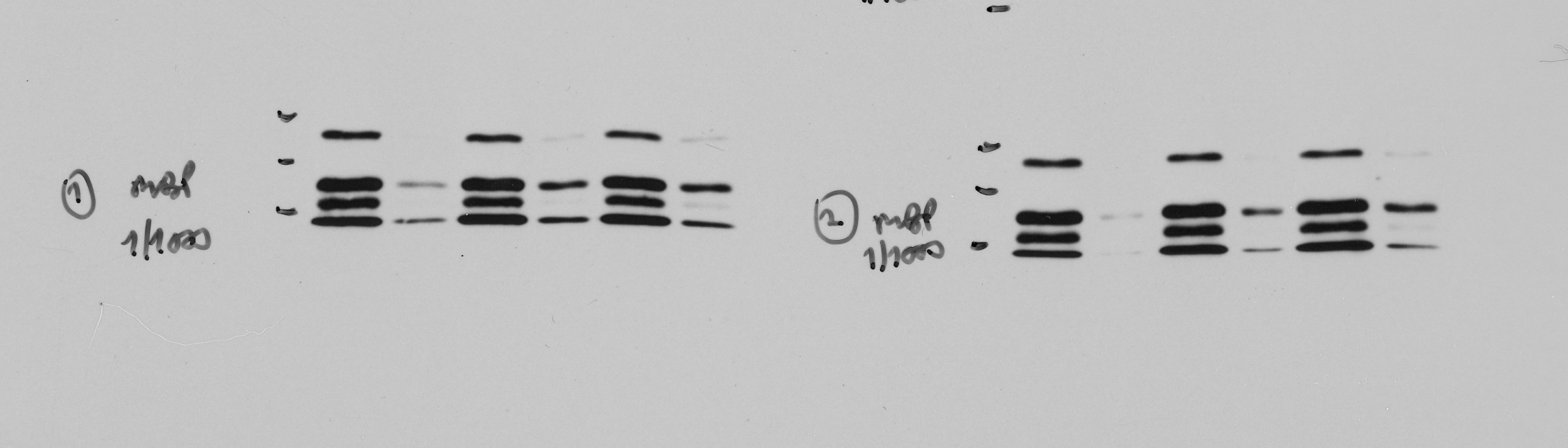

Supplement: Figure 4—source data 1. [file elife-87394-fig4-data1.zip › Fig 4 souce data 1/Fig 4A blots and prism files for graphs/MBP/uncropped.tif]

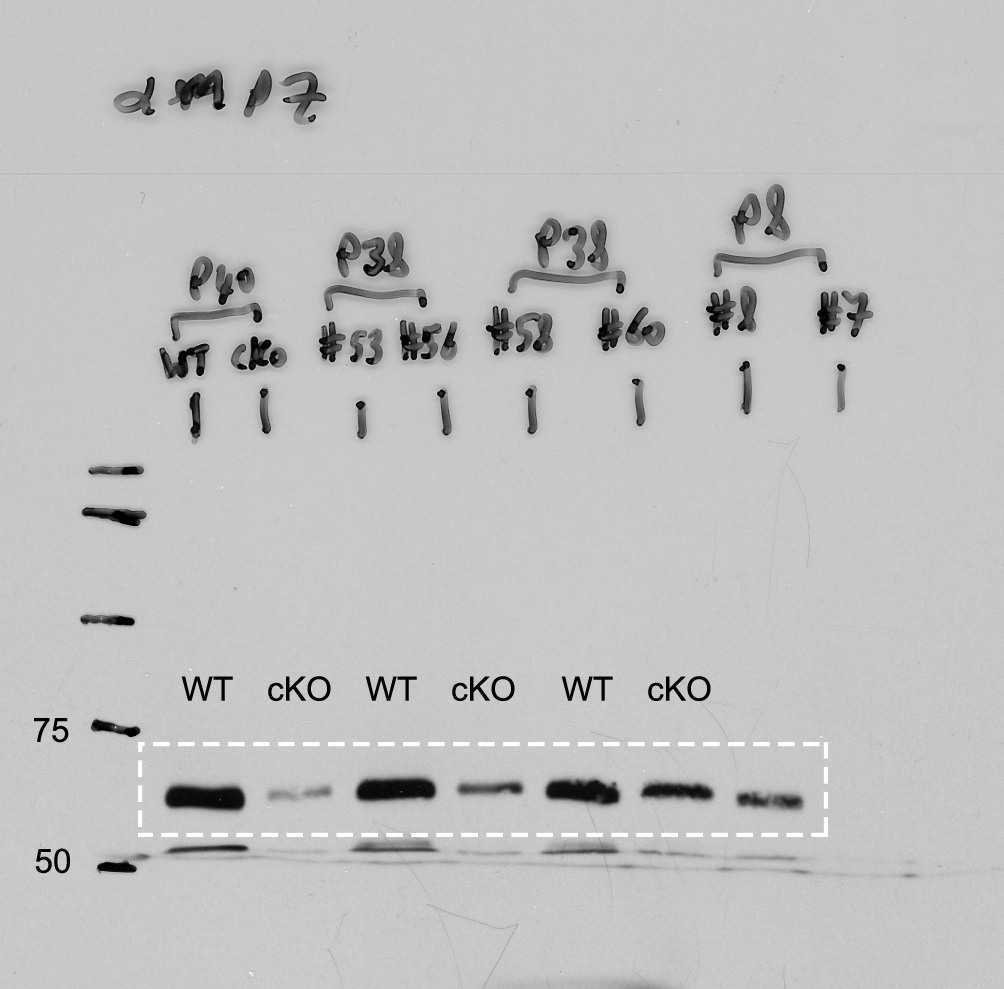

Supplement: Figure 4—source data 1. [file elife-87394-fig4-data1.zip › Fig 4 souce data 1/Fig 4A blots and prism files for graphs/MPZ/uncropped labeled.tif]

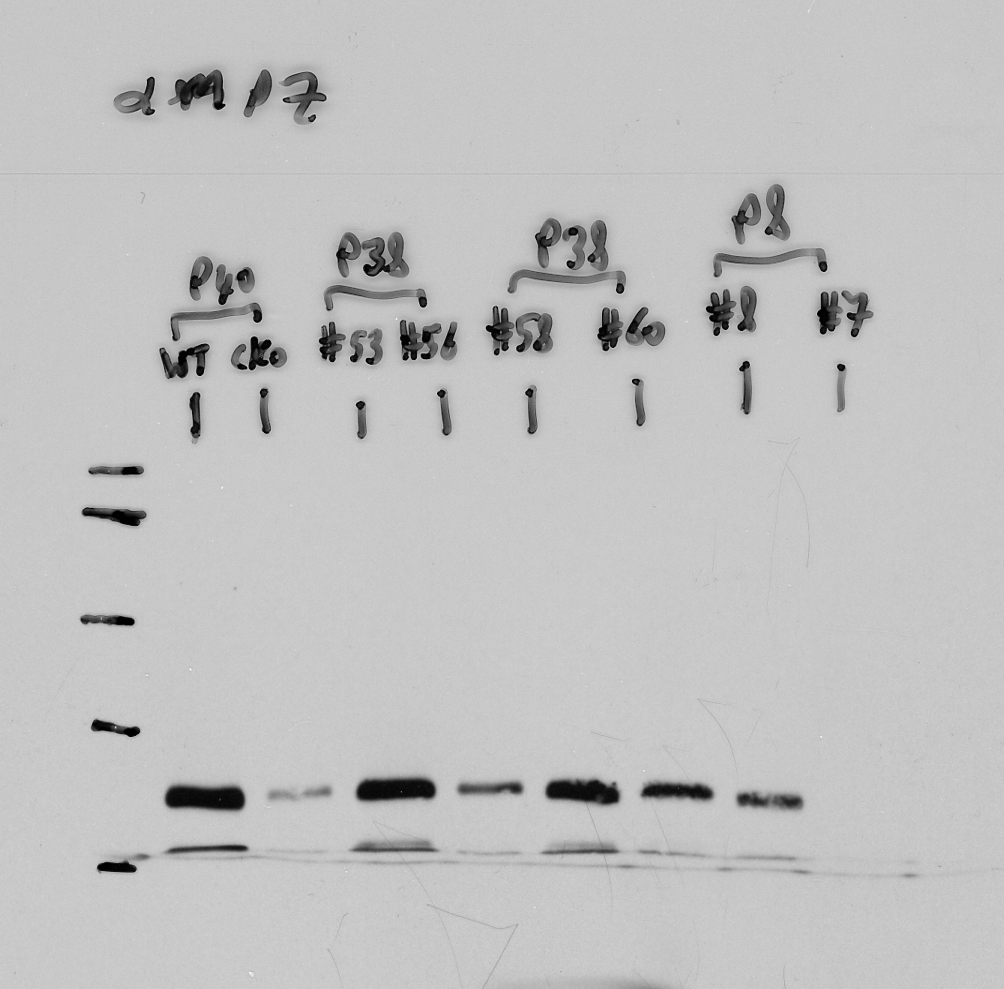

Supplement: Figure 4—source data 1. [file elife-87394-fig4-data1.zip › Fig 4 souce data 1/Fig 4A blots and prism files for graphs/MPZ/uncropped.tif]

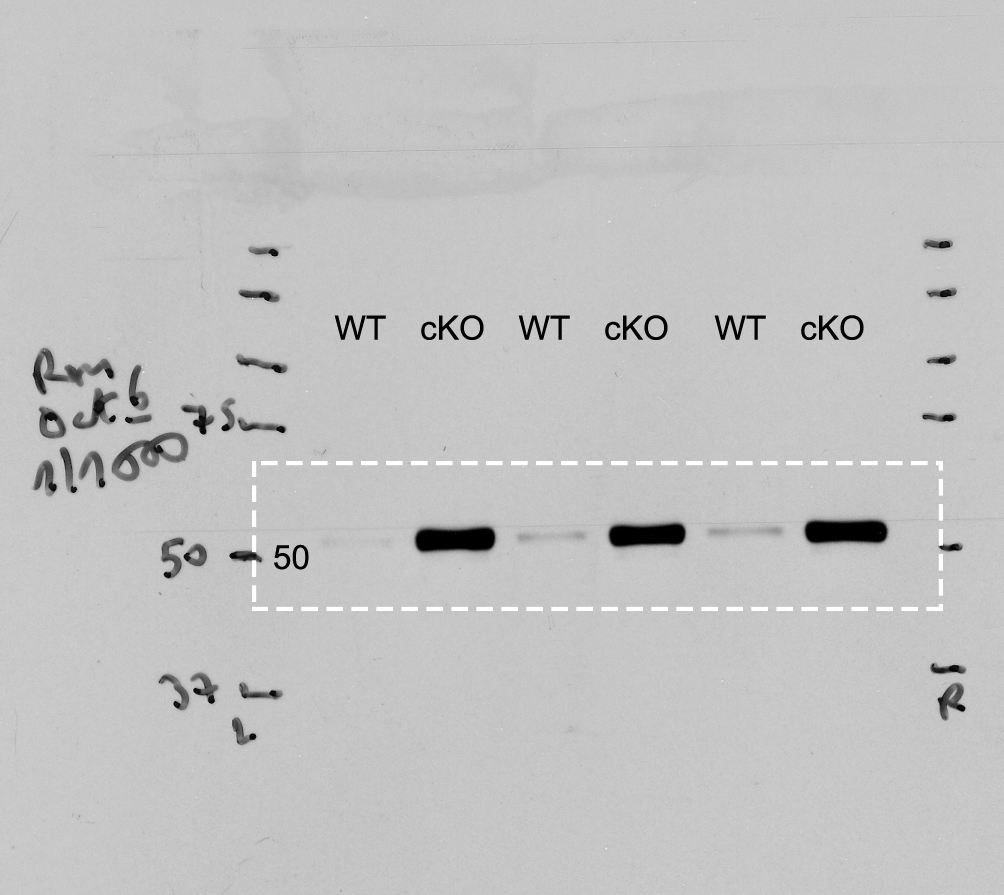

Supplement: Figure 4—source data 1. [file elife-87394-fig4-data1.zip › Fig 4 souce data 1/Fig 4A blots and prism files for graphs/Oct6/uncropped labeled.tif]

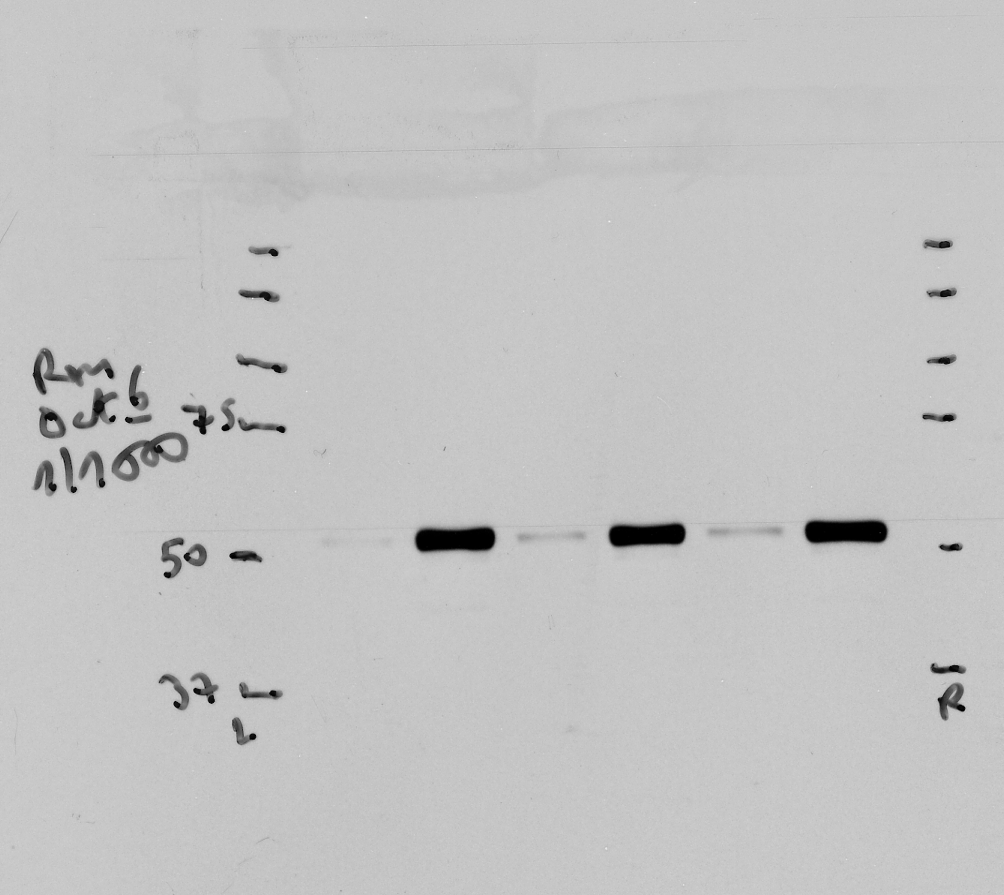

Supplement: Figure 4—source data 1. [file elife-87394-fig4-data1.zip › Fig 4 souce data 1/Fig 4A blots and prism files for graphs/Oct6/uncropped.tif]

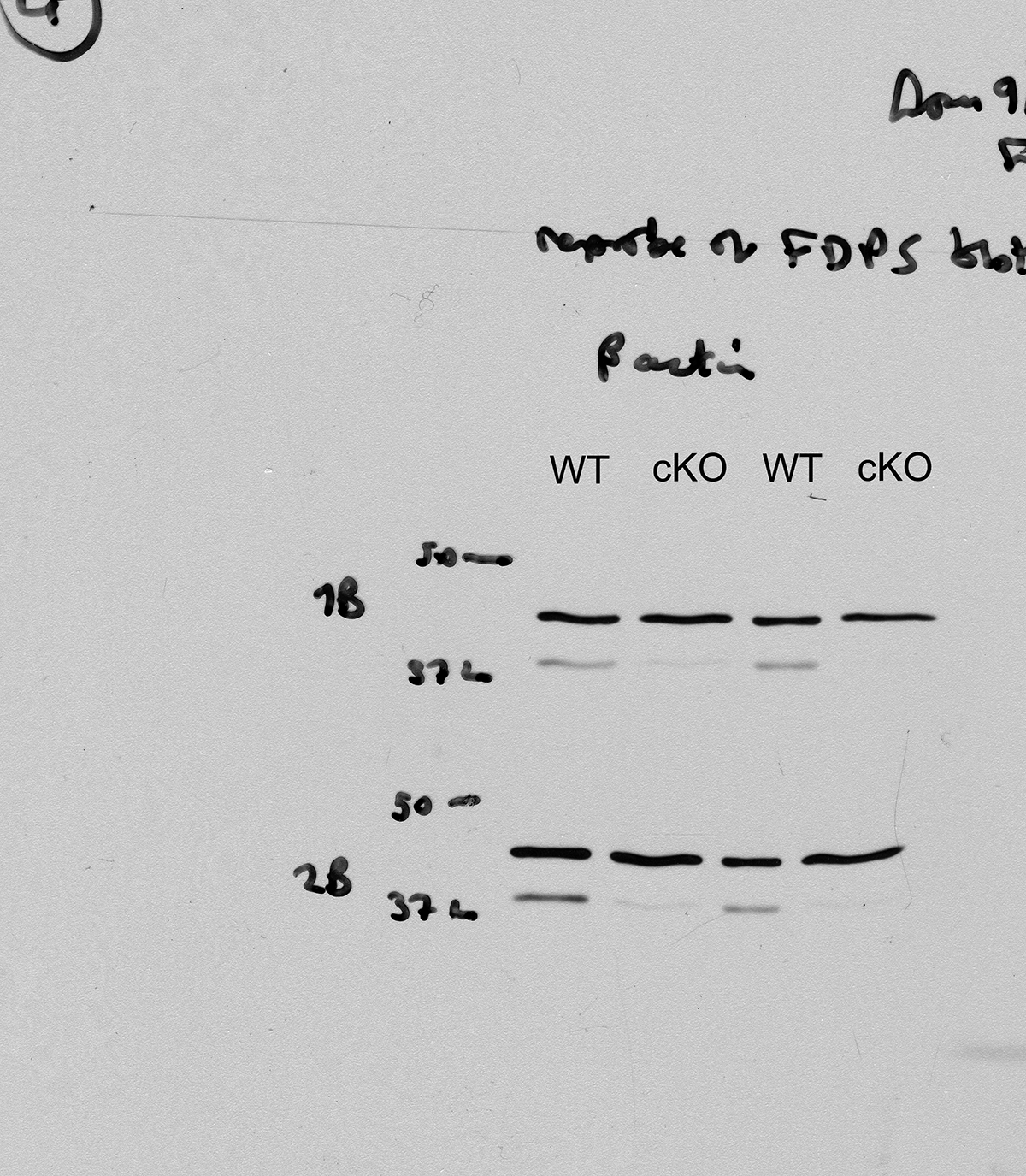

Supplement: Figure 5—source data 1. [file elife-87394-fig5-data1.zip › Fig 5 source data 1/Fig 5A P8 blots/P8 actin for IDI1/uncropped 1 labeled.tif]

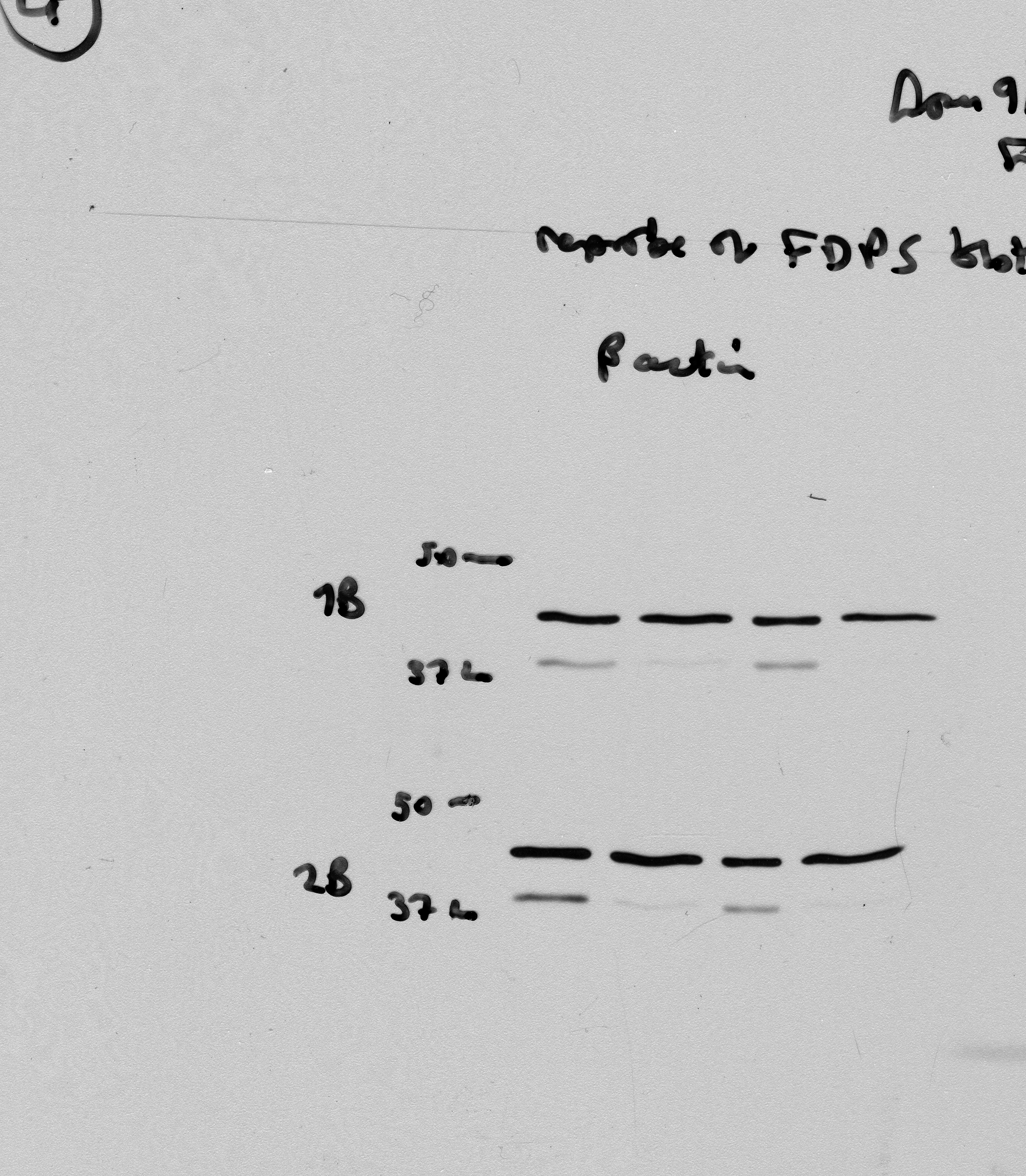

Supplement: Figure 5—source data 1. [file elife-87394-fig5-data1.zip › Fig 5 source data 1/Fig 5A P8 blots/P8 actin for IDI1/uncropped 1.tif]

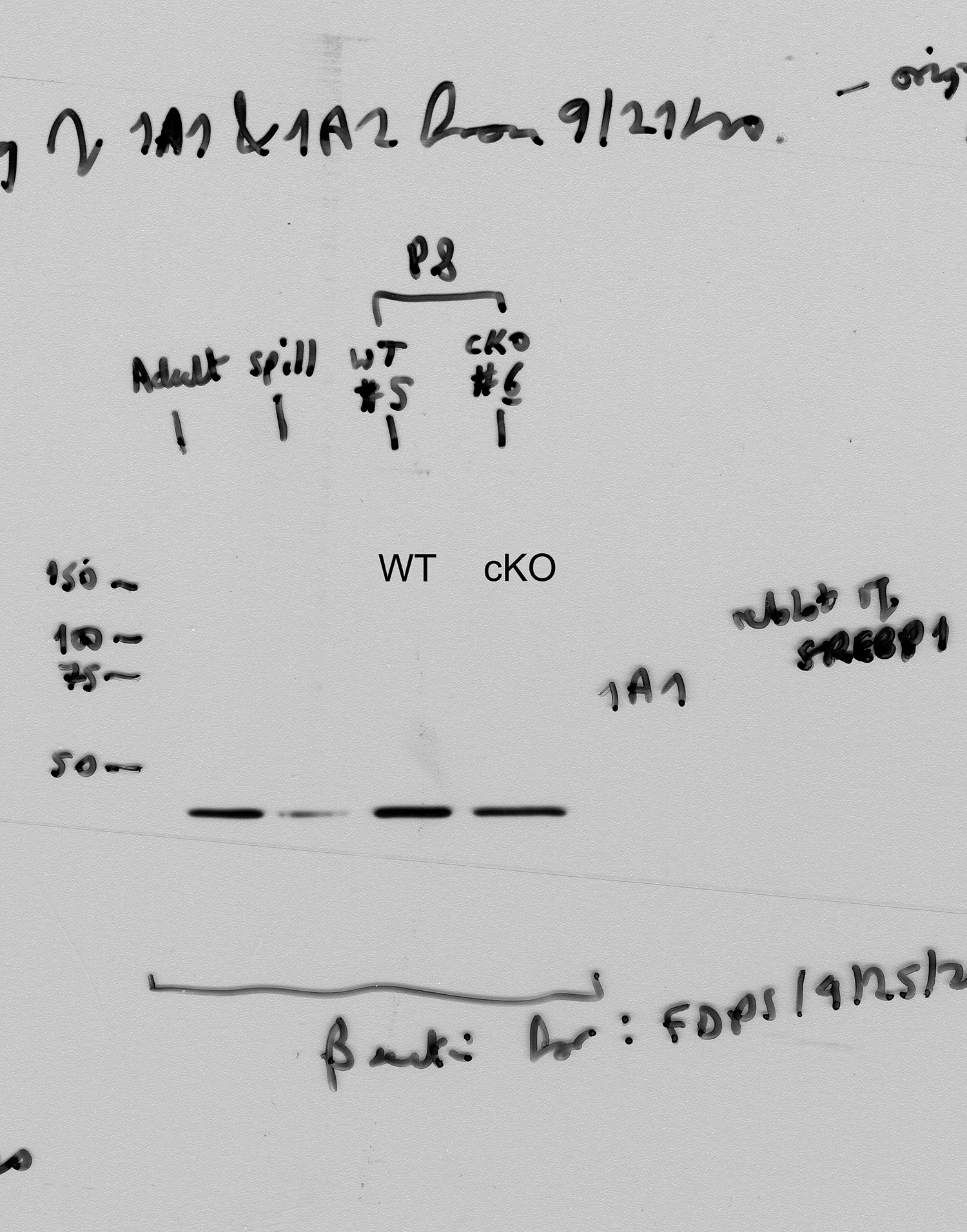

Supplement: Figure 5—source data 1. [file elife-87394-fig5-data1.zip › Fig 5 source data 1/Fig 5A P8 blots/P8 actin for IDI1/uncropped 2 labeled.tif]

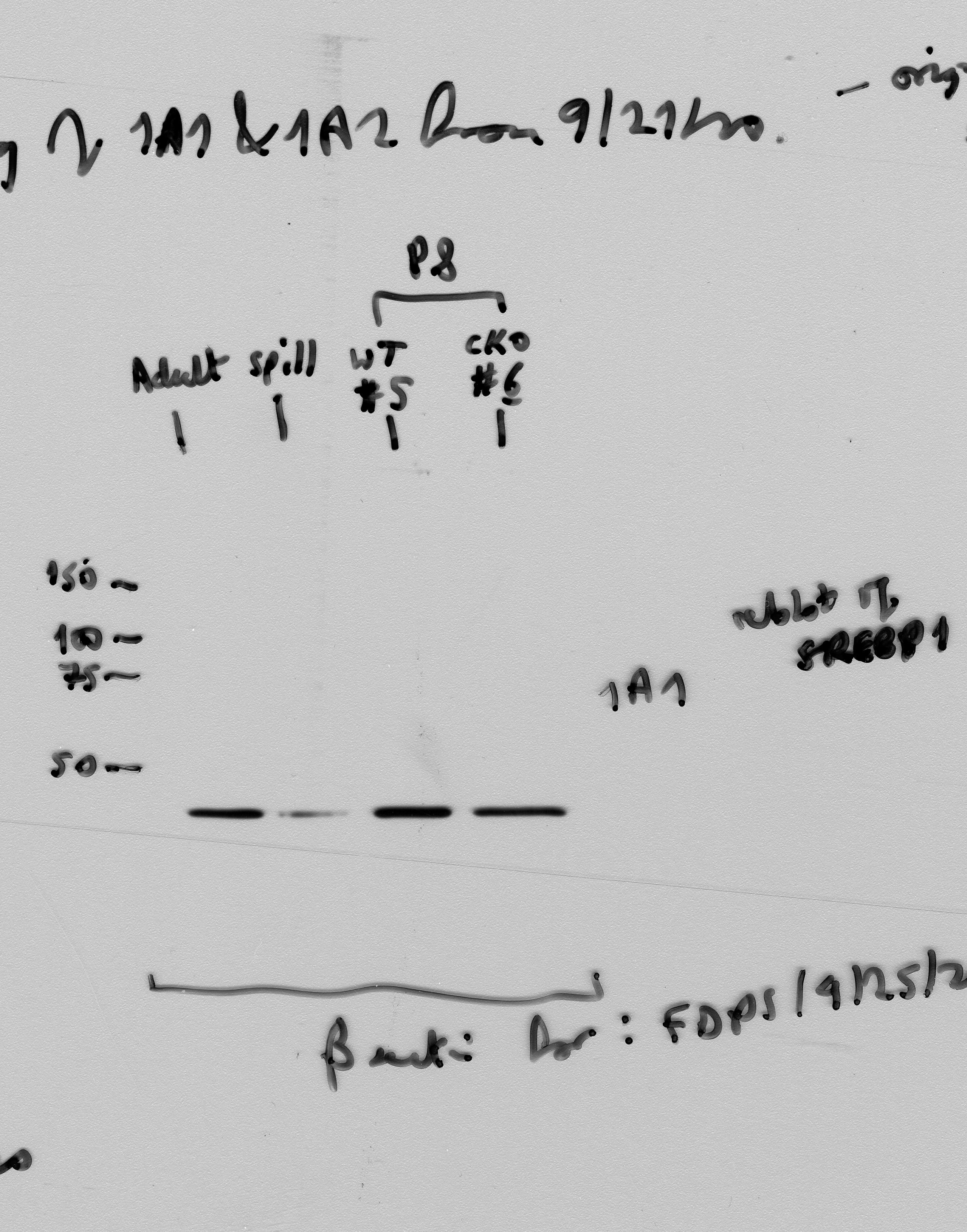

Supplement: Figure 5—source data 1. [file elife-87394-fig5-data1.zip › Fig 5 source data 1/Fig 5A P8 blots/P8 actin for IDI1/uncropped 2.tif]

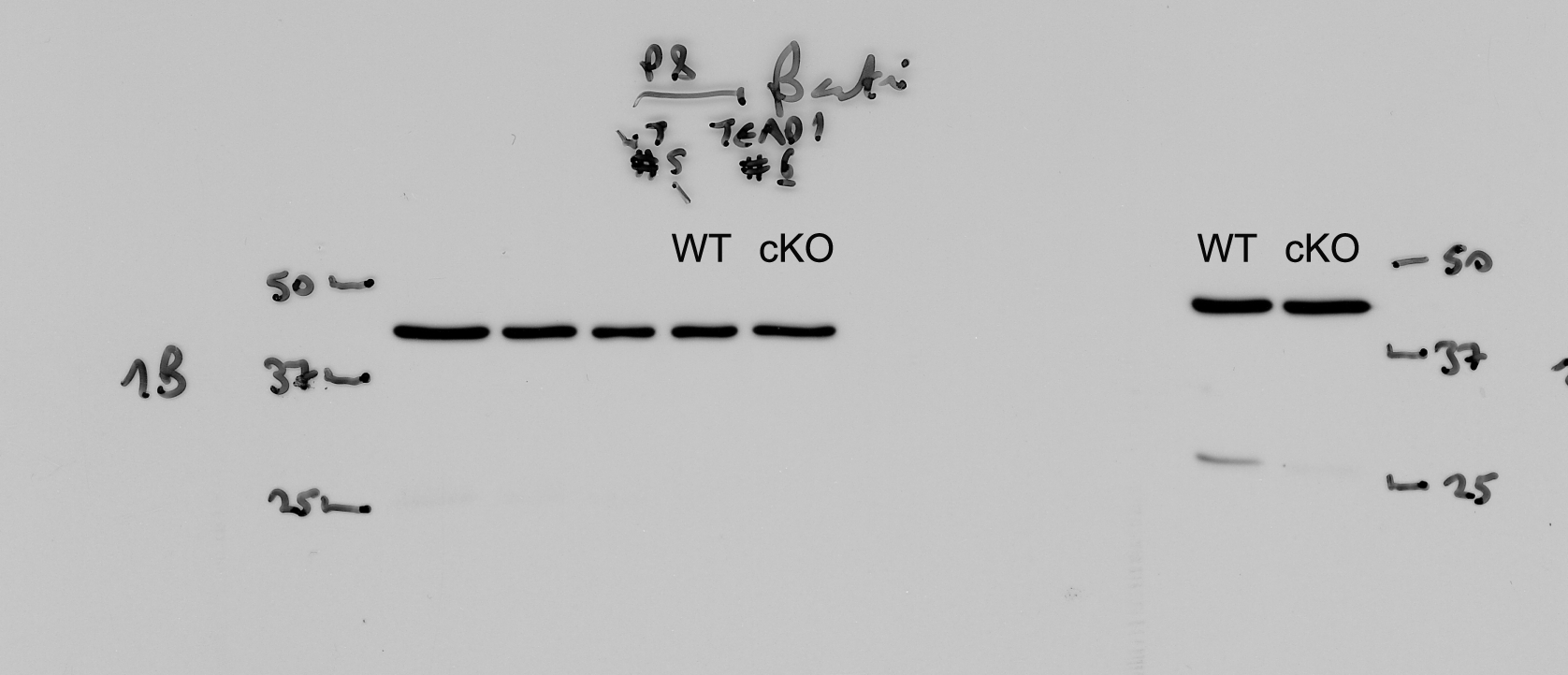

Supplement: Figure 5—source data 1. [file elife-87394-fig5-data1.zip › Fig 5 source data 1/Fig 5A P8 blots/P8 actin for SCD1/uncropped 1 labeled.tif]

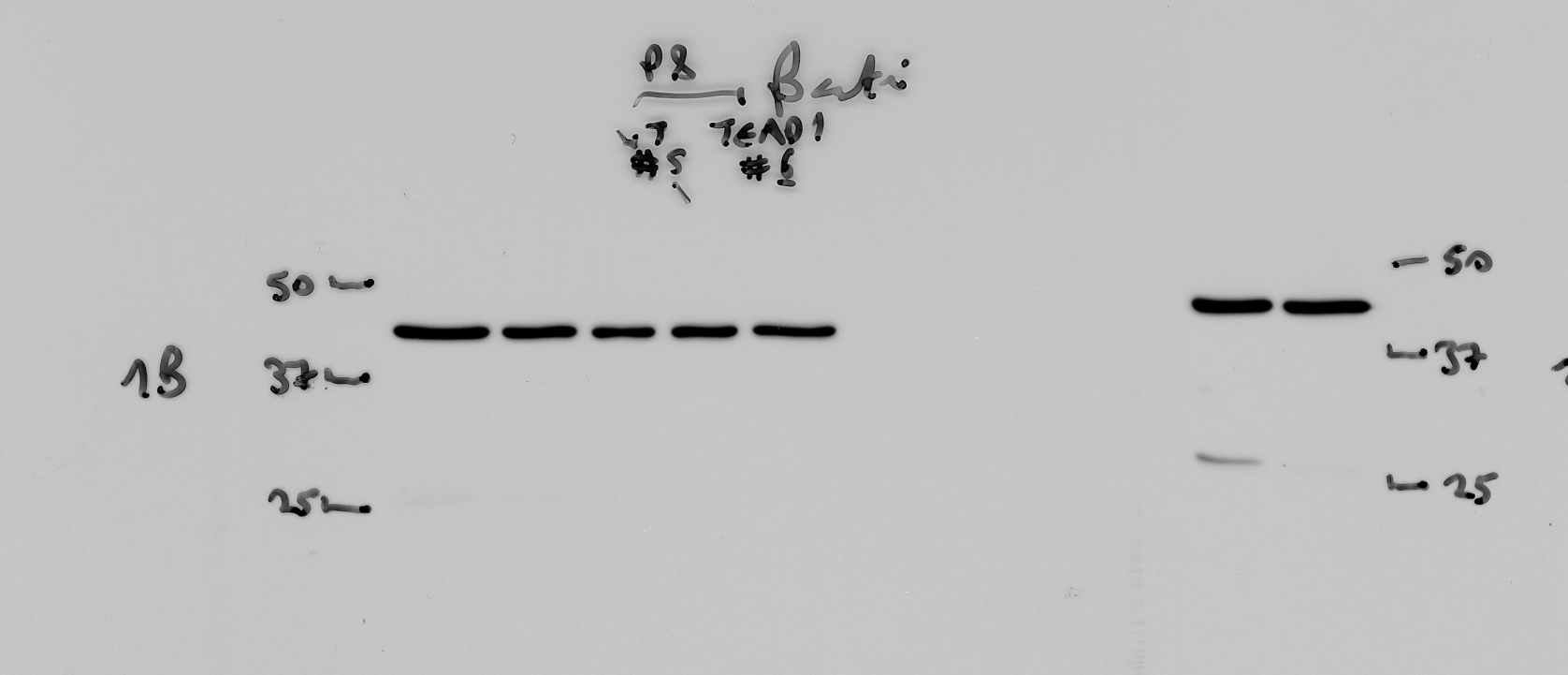

Supplement: Figure 5—source data 1. [file elife-87394-fig5-data1.zip › Fig 5 source data 1/Fig 5A P8 blots/P8 actin for SCD1/uncropped 1.tif]

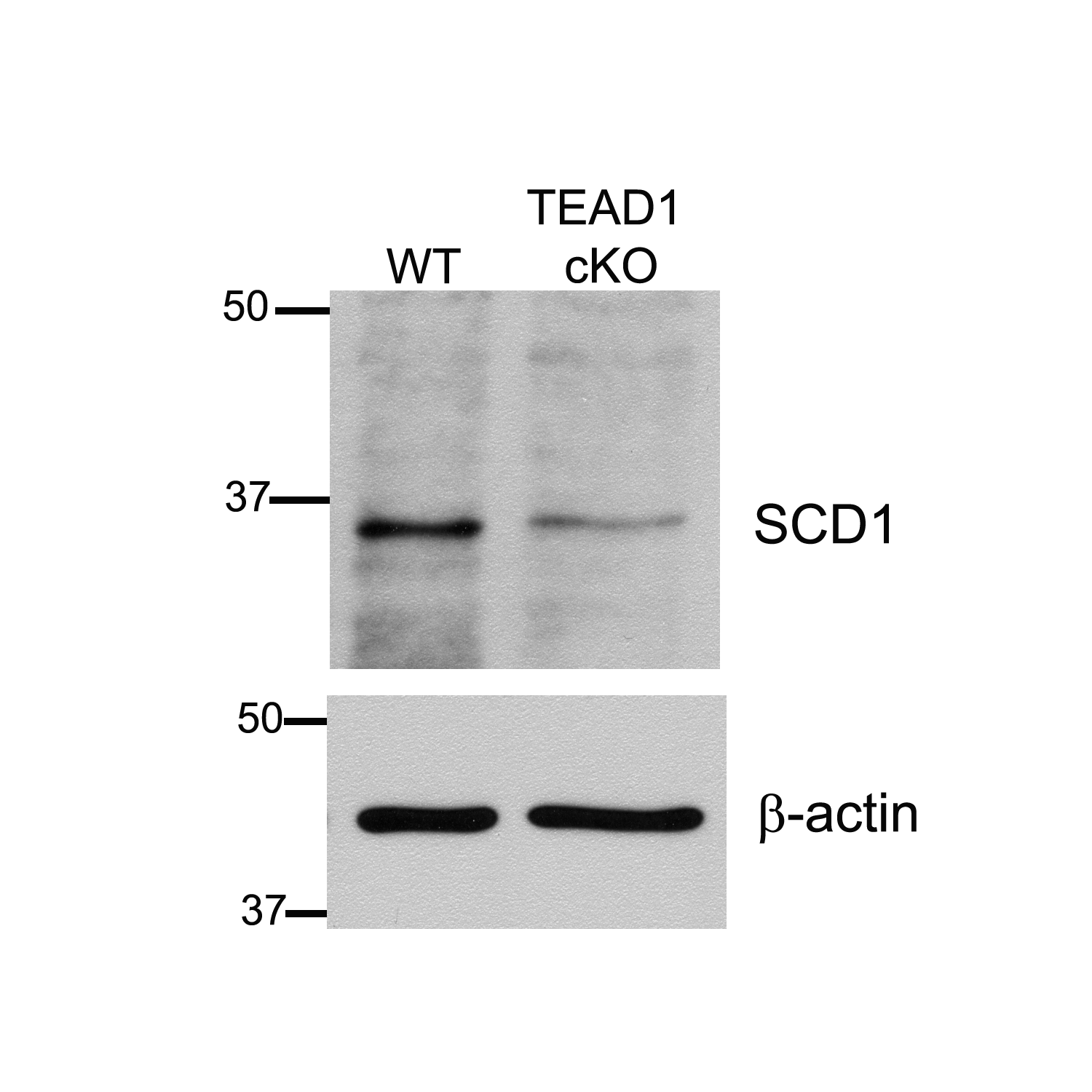

Supplement: Figure 5—source data 1. [file elife-87394-fig5-data1.zip › Fig 5 source data 1/Fig 5A P8 blots/P8 actin for SCD1/uncropped 2 labeled.tif]

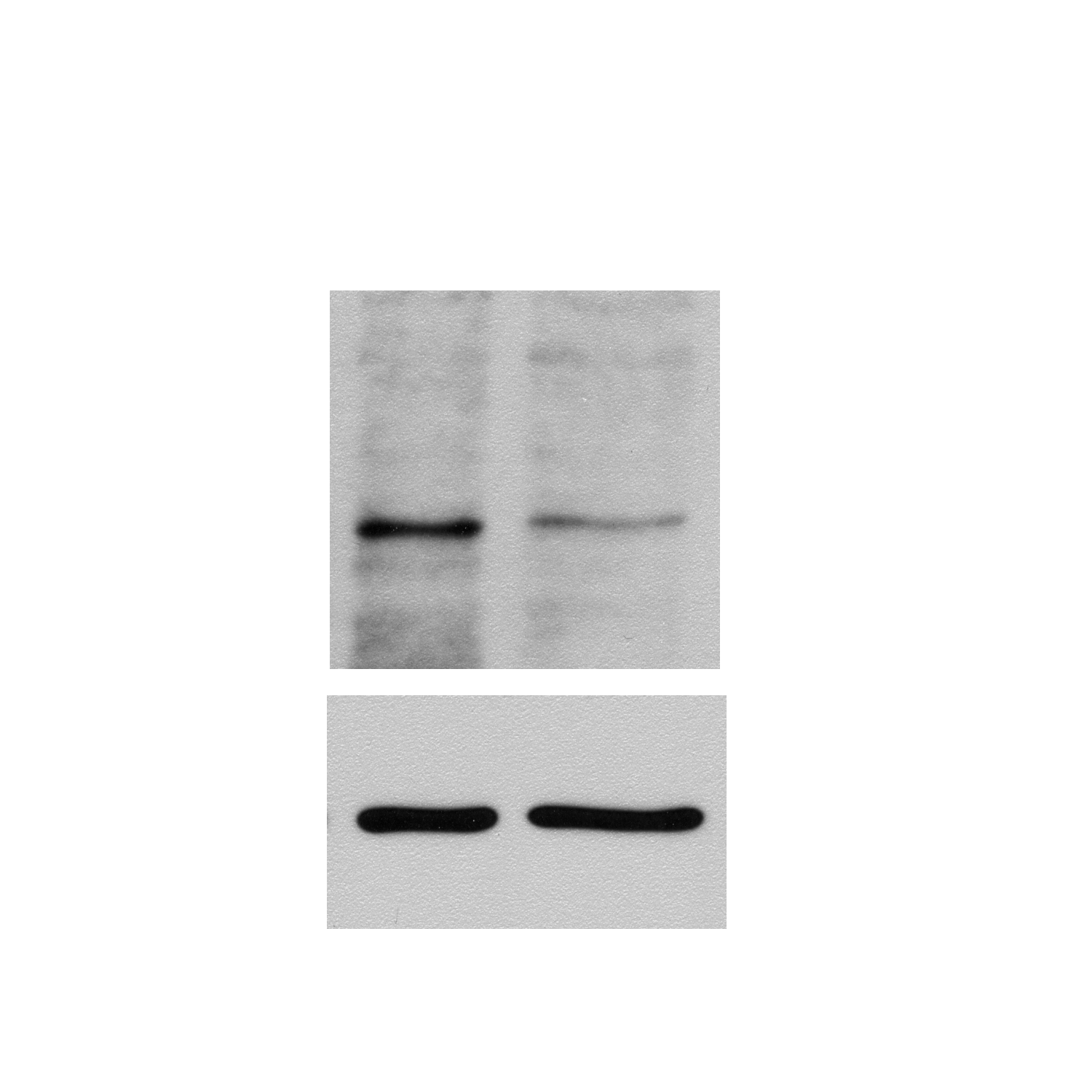

Supplement: Figure 5—source data 1. [file elife-87394-fig5-data1.zip › Fig 5 source data 1/Fig 5A P8 blots/P8 actin for SCD1/uncropped 2.tif]

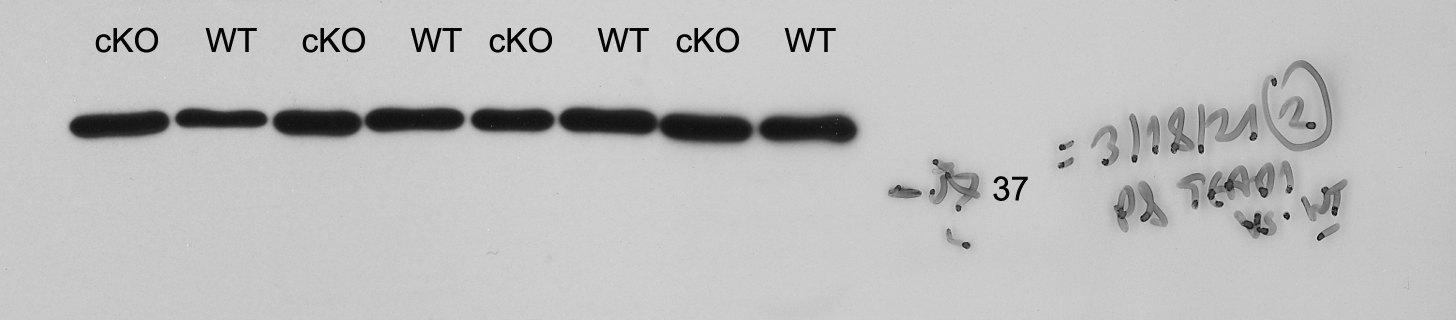

Supplement: Figure 5—source data 1. [file elife-87394-fig5-data1.zip › Fig 5 source data 1/Fig 5A P8 blots/P8 actin for SREBP1/uncropped labeled.tif]

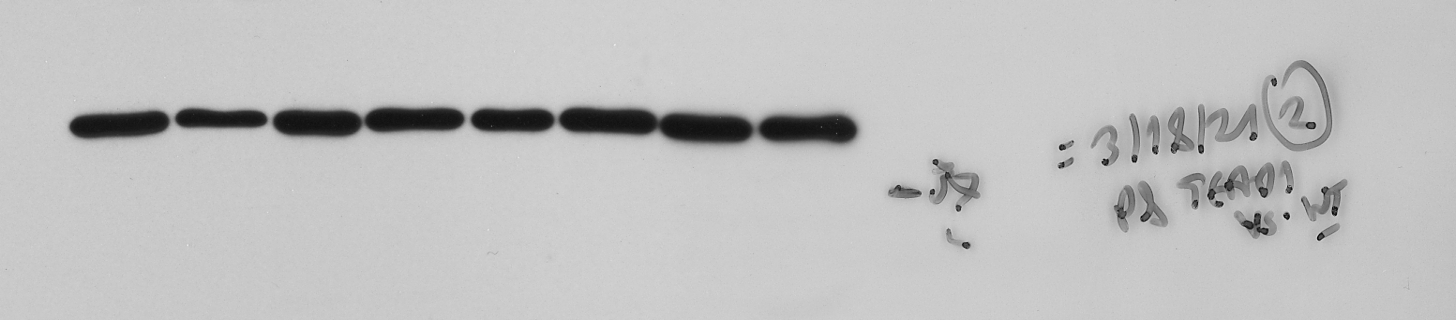

Supplement: Figure 5—source data 1. [file elife-87394-fig5-data1.zip › Fig 5 source data 1/Fig 5A P8 blots/P8 actin for SREBP1/uncropped.tif]

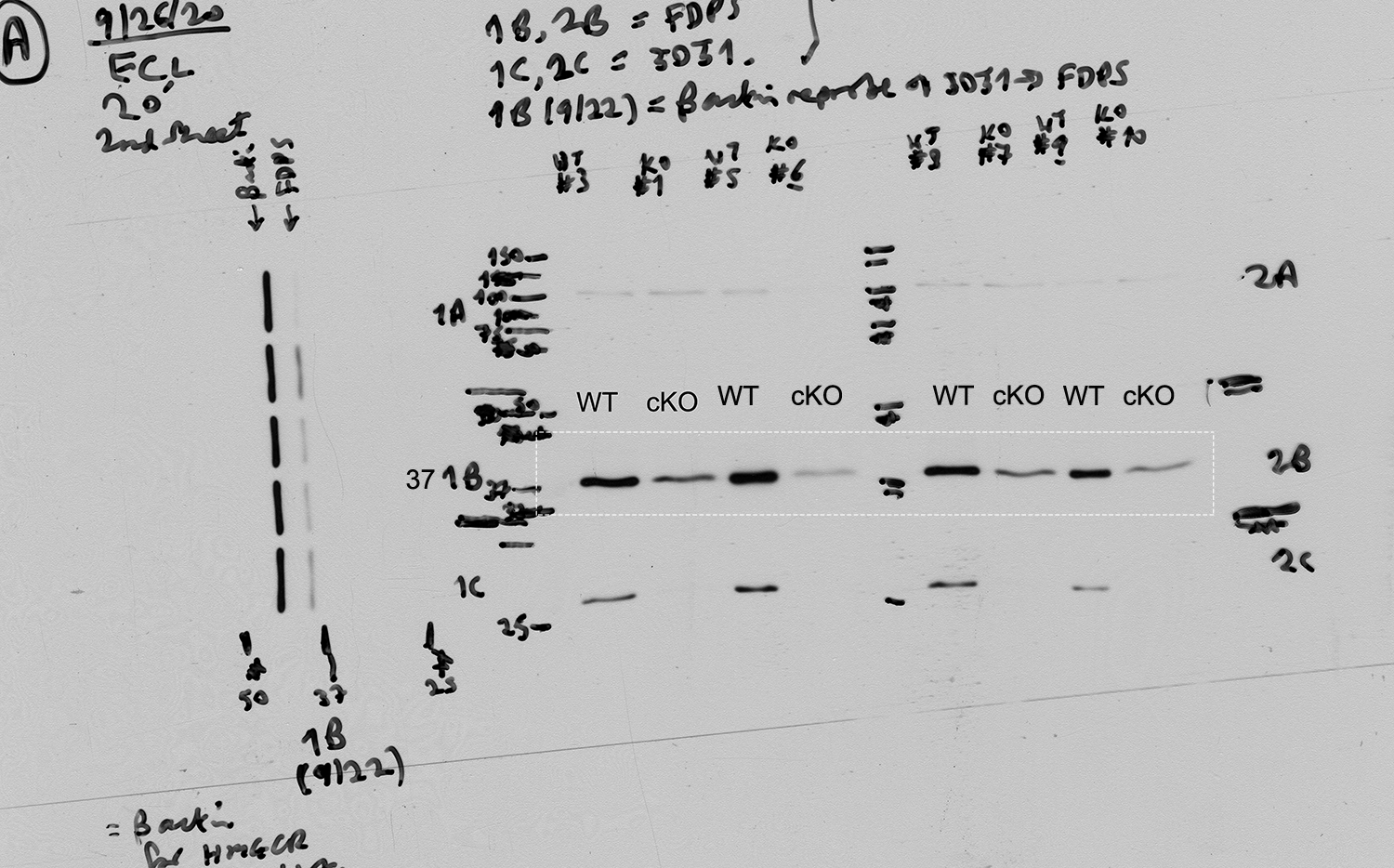

Supplement: Figure 5—source data 1. [file elife-87394-fig5-data1.zip › Fig 5 source data 1/Fig 5A P8 blots/P8 FDPS/uncropped labeled.tif]

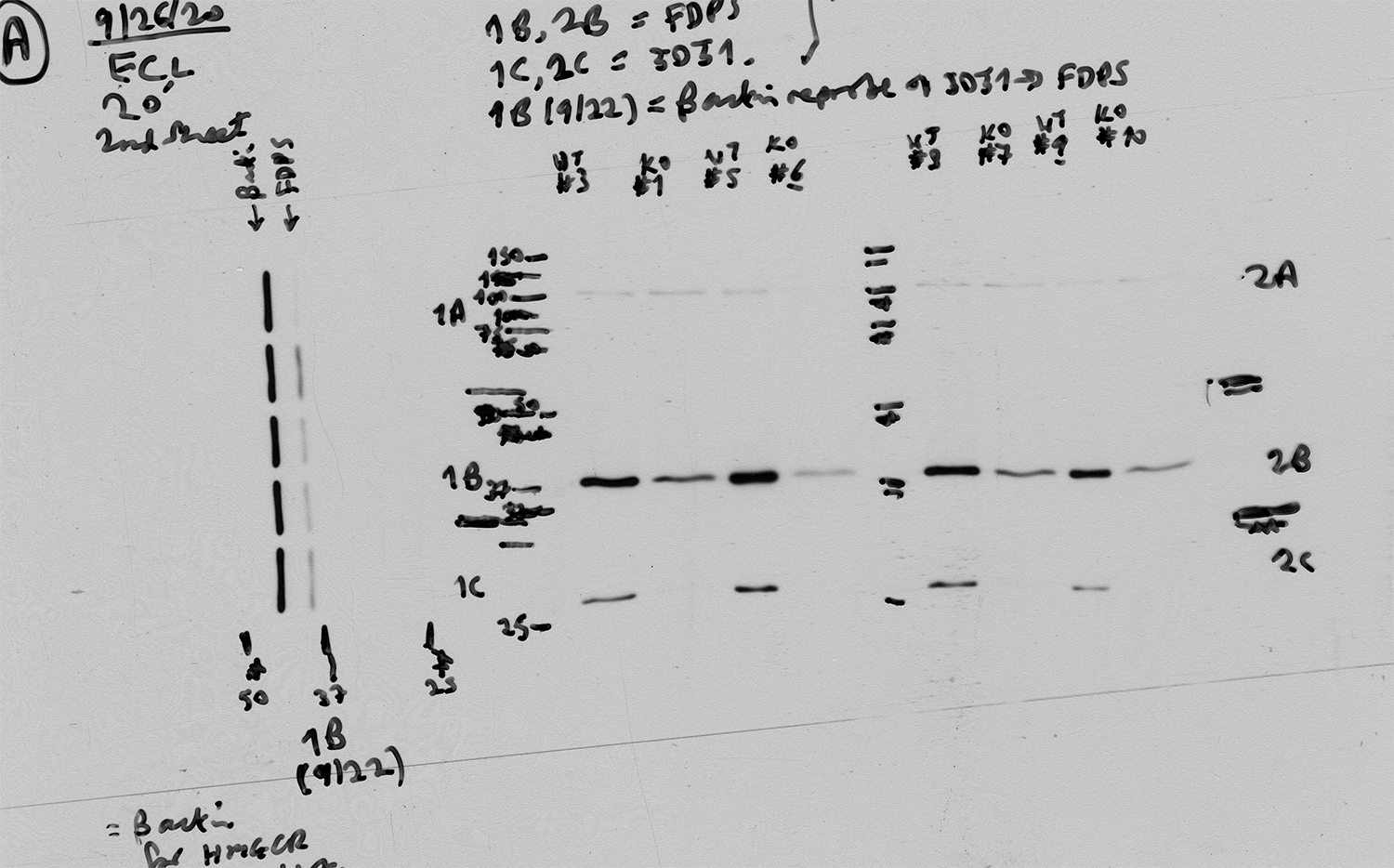

Supplement: Figure 5—source data 1. [file elife-87394-fig5-data1.zip › Fig 5 source data 1/Fig 5A P8 blots/P8 FDPS/uncropped.tif]

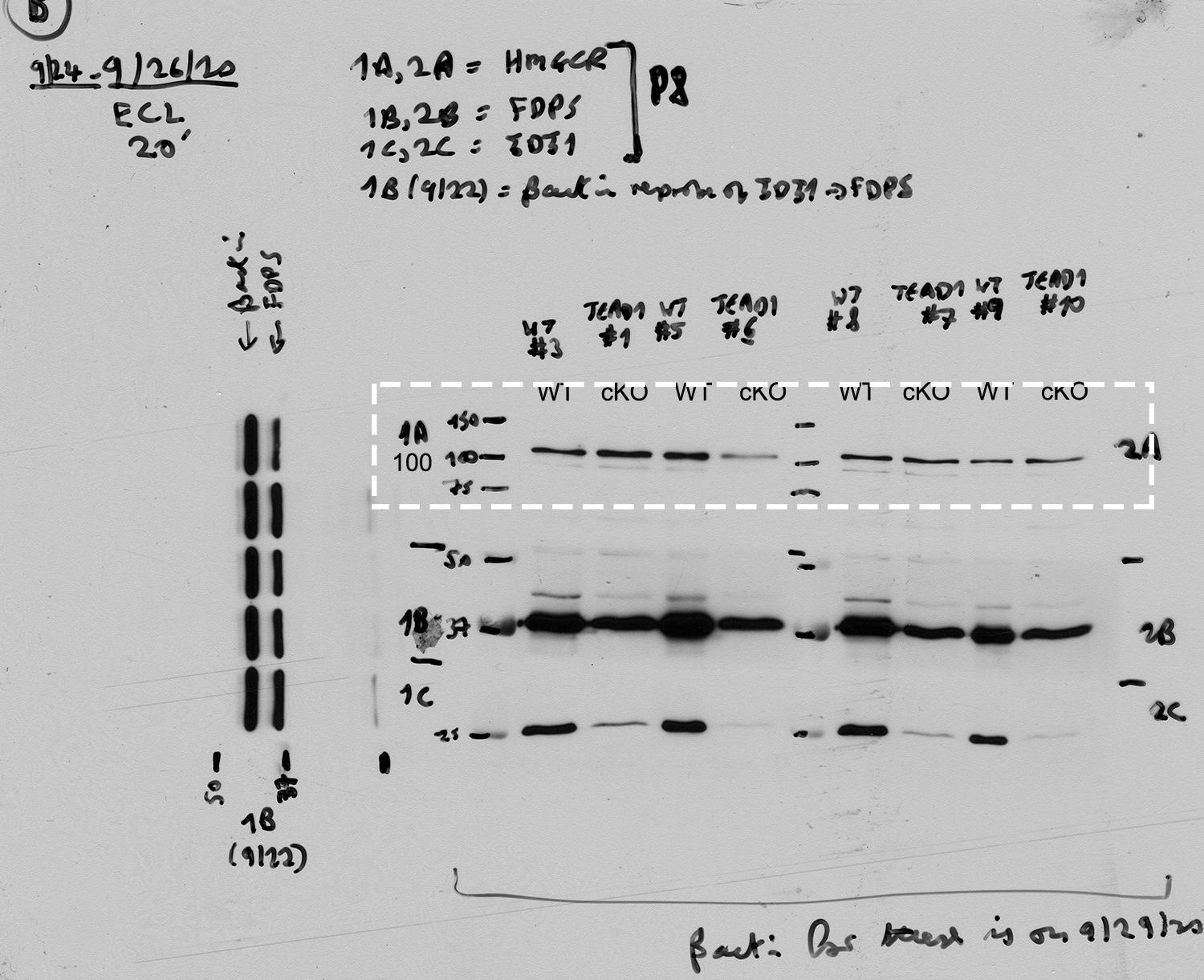

Supplement: Figure 5—source data 1. [file elife-87394-fig5-data1.zip › Fig 5 source data 1/Fig 5A P8 blots/P8 HMGCR/uncropped labeled.tif]

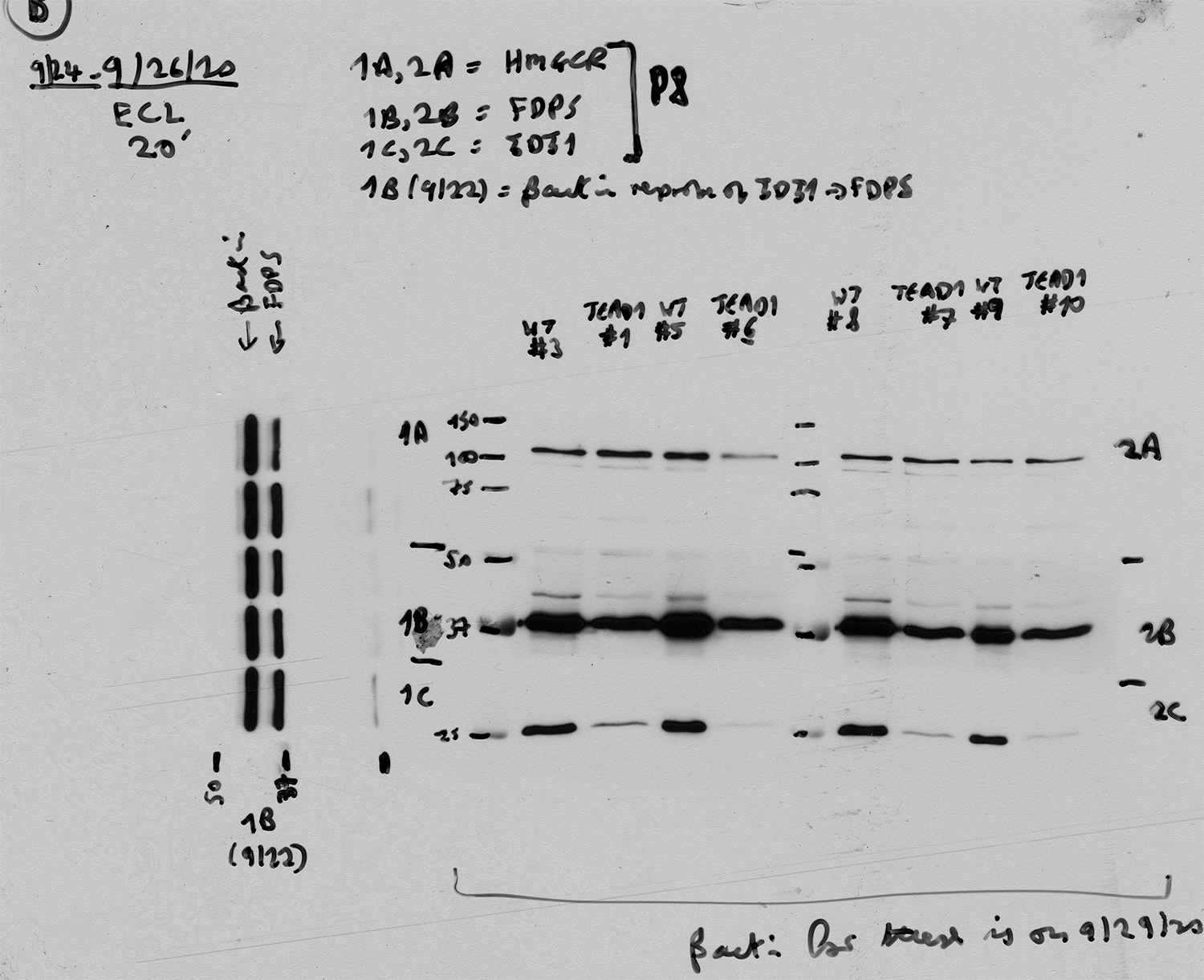

Supplement: Figure 5—source data 1. [file elife-87394-fig5-data1.zip › Fig 5 source data 1/Fig 5A P8 blots/P8 HMGCR/uncropped.tif]

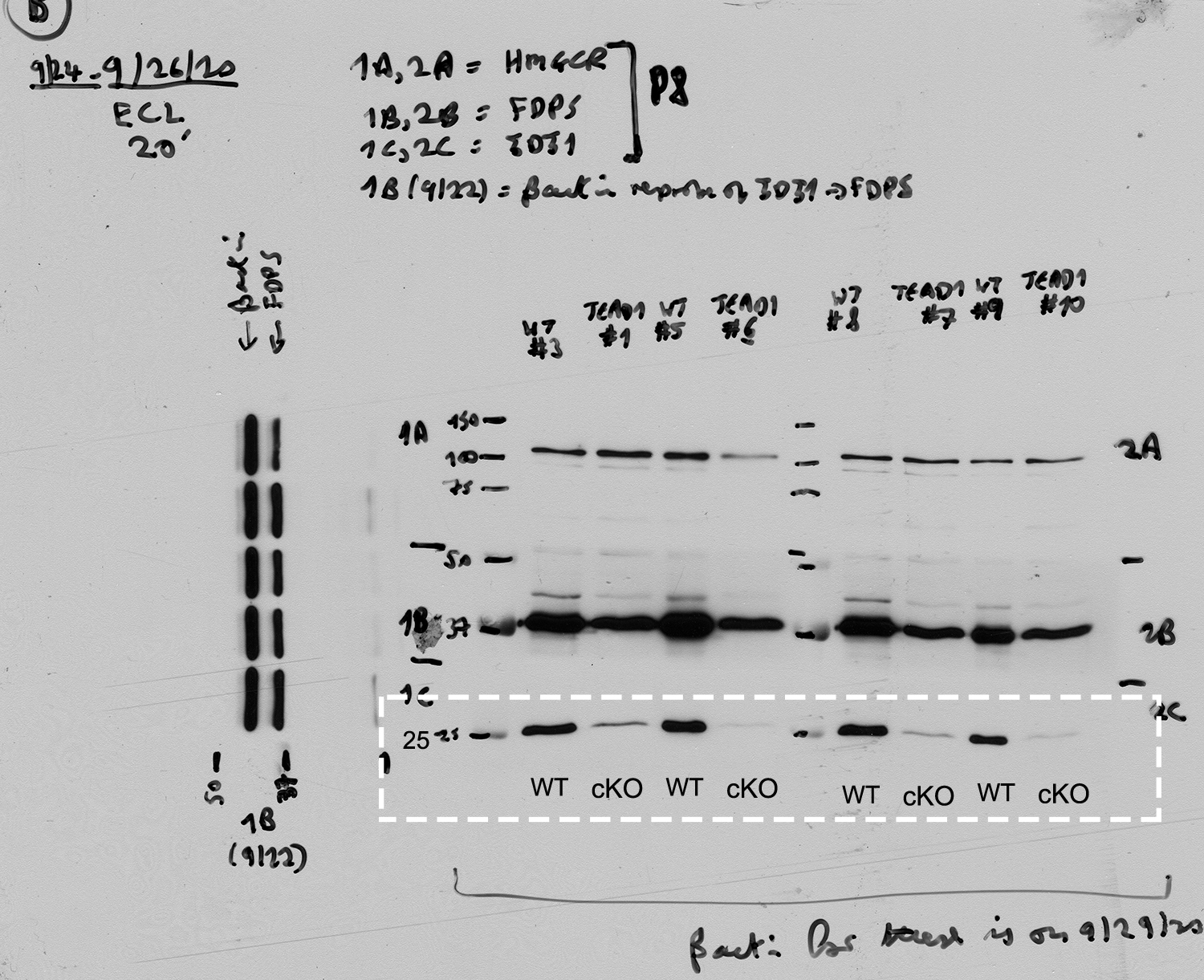

Supplement: Figure 5—source data 1. [file elife-87394-fig5-data1.zip › Fig 5 source data 1/Fig 5A P8 blots/P8 IDI1/uncropped labeled.tif]

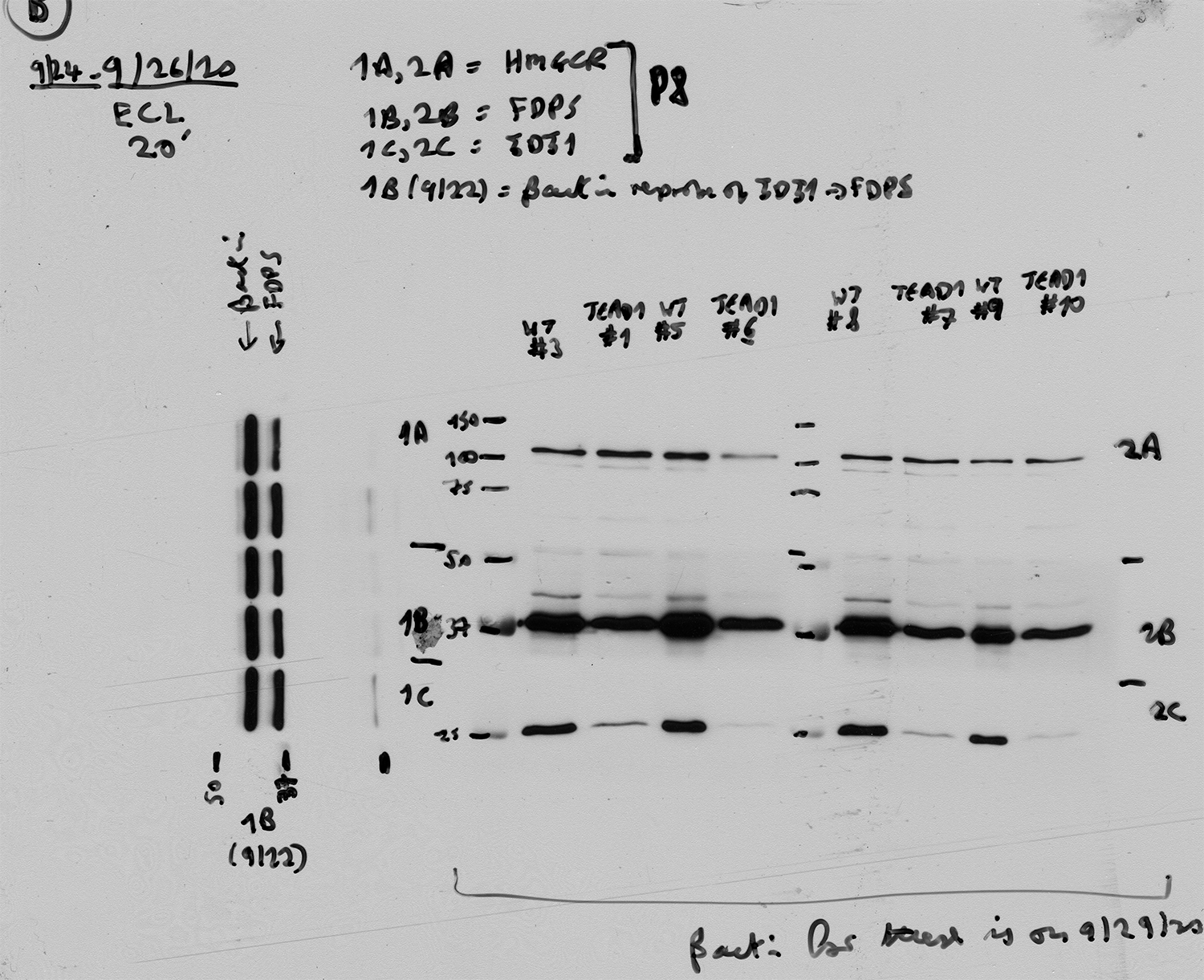

Supplement: Figure 5—source data 1. [file elife-87394-fig5-data1.zip › Fig 5 source data 1/Fig 5A P8 blots/P8 IDI1/uncropped.tif]

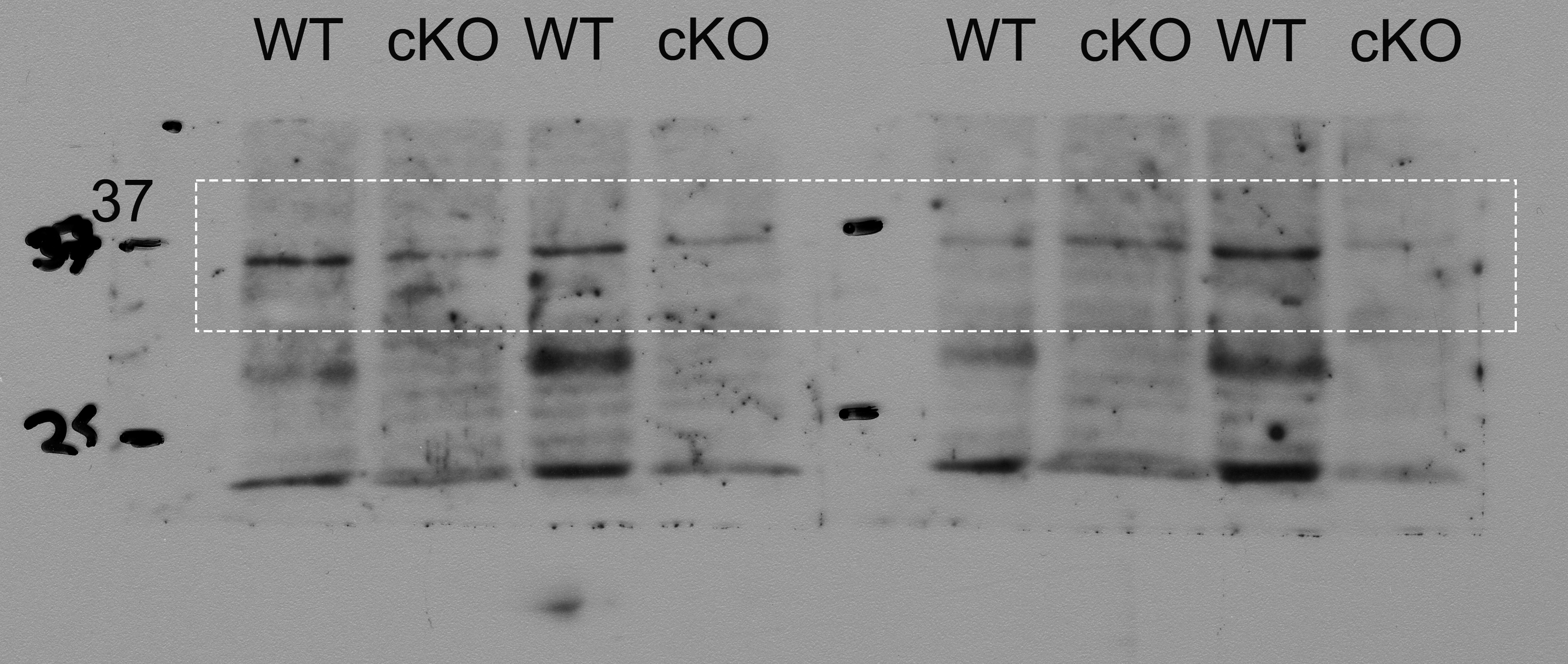

Supplement: Figure 5—source data 1. [file elife-87394-fig5-data1.zip › Fig 5 source data 1/Fig 5A P8 blots/P8 SCD1/uncropped 1 labeled.tif]

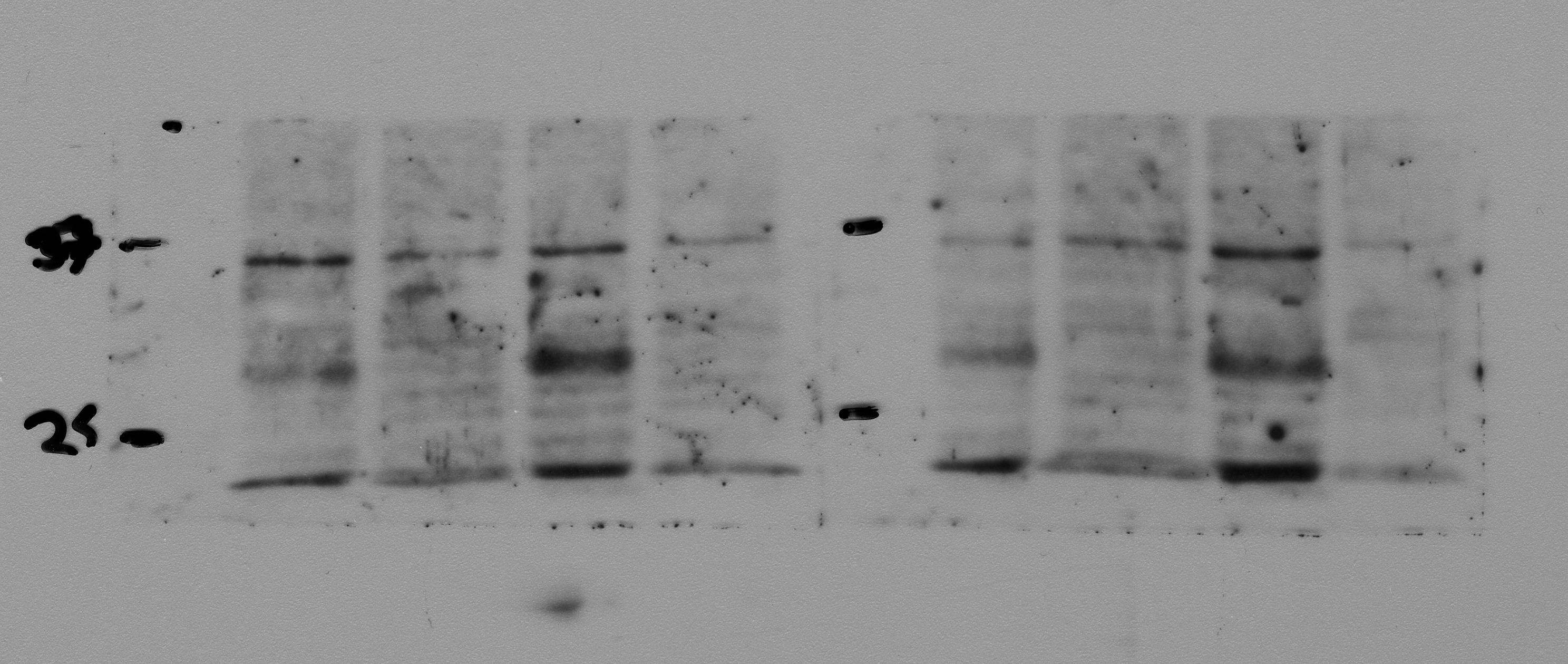

Supplement: Figure 5—source data 1. [file elife-87394-fig5-data1.zip › Fig 5 source data 1/Fig 5A P8 blots/P8 SCD1/uncropped 1.tif]

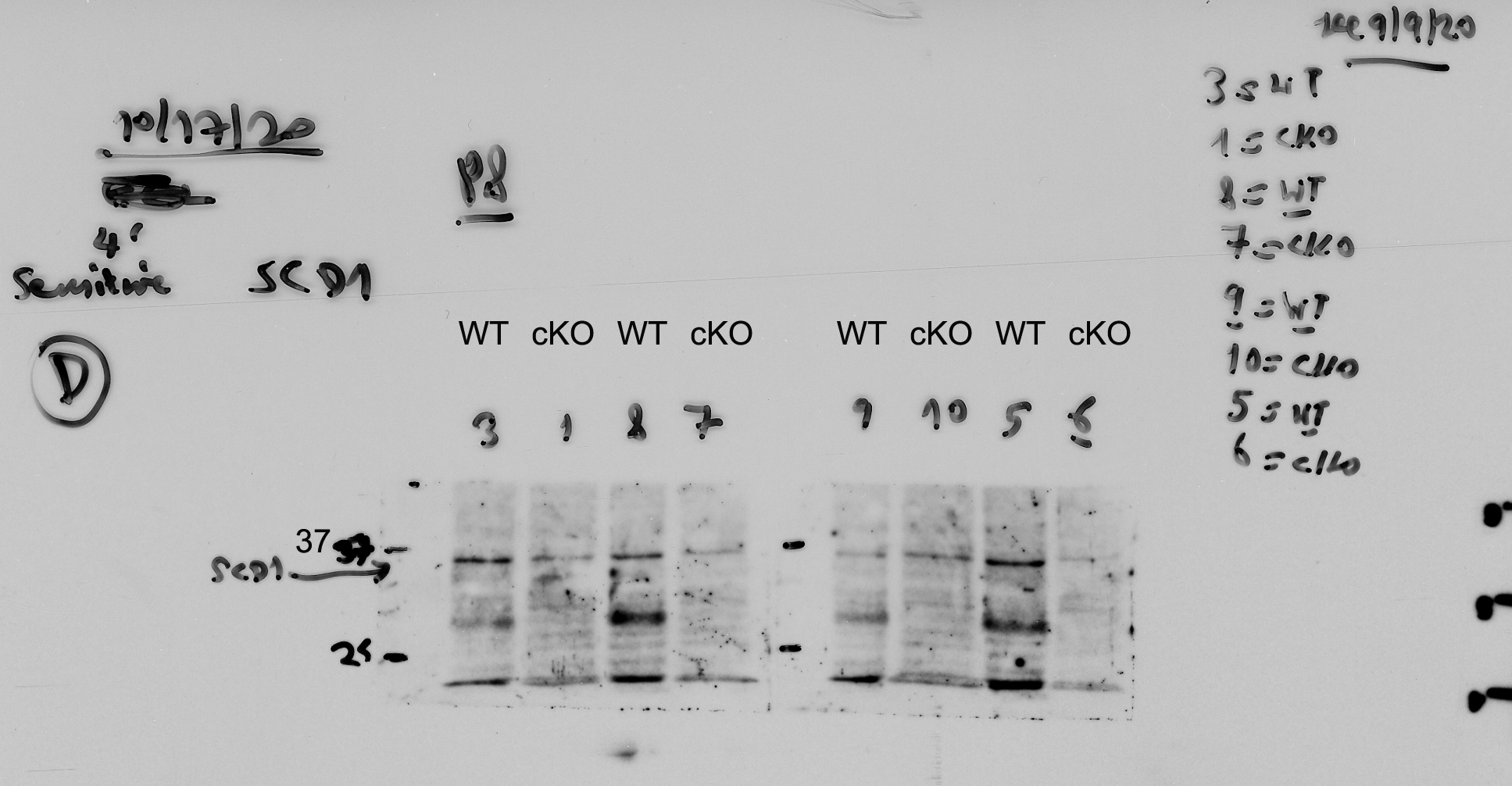

Supplement: Figure 5—source data 1. [file elife-87394-fig5-data1.zip › Fig 5 source data 1/Fig 5A P8 blots/P8 SCD1/uncropped 2 labeled.tif]

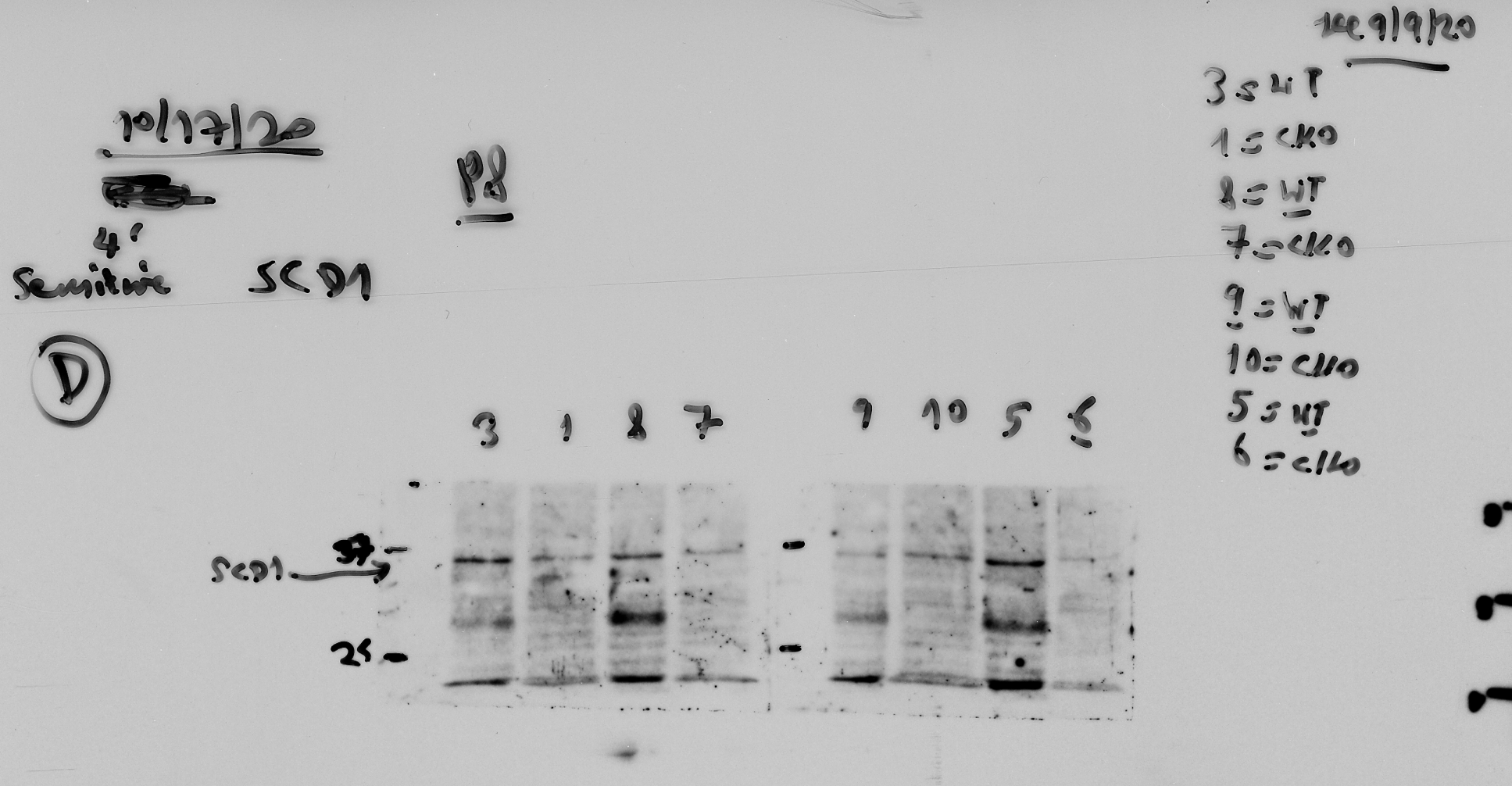

Supplement: Figure 5—source data 1. [file elife-87394-fig5-data1.zip › Fig 5 source data 1/Fig 5A P8 blots/P8 SCD1/uncropped 2.tif]

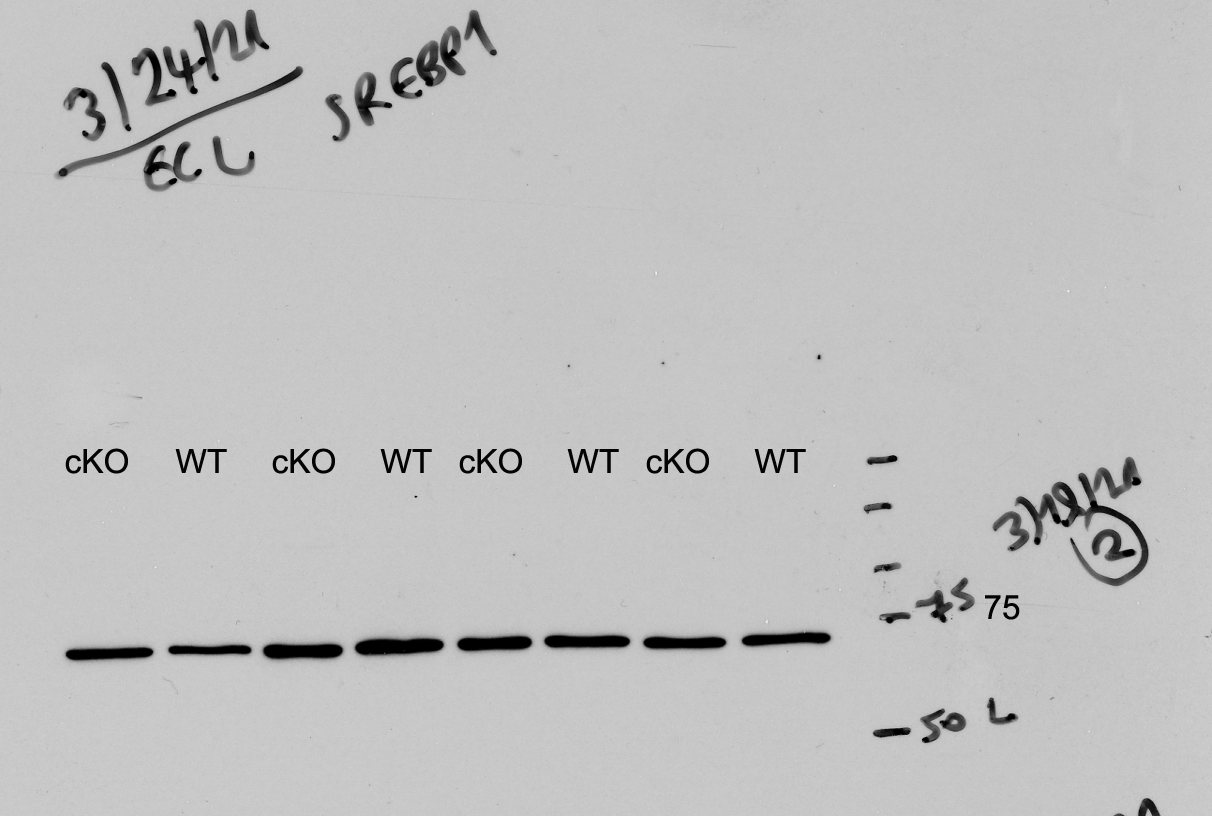

Supplement: Figure 5—source data 1. [file elife-87394-fig5-data1.zip › Fig 5 source data 1/Fig 5A P8 blots/P8 SREBP1/uncropped labeled.tif]

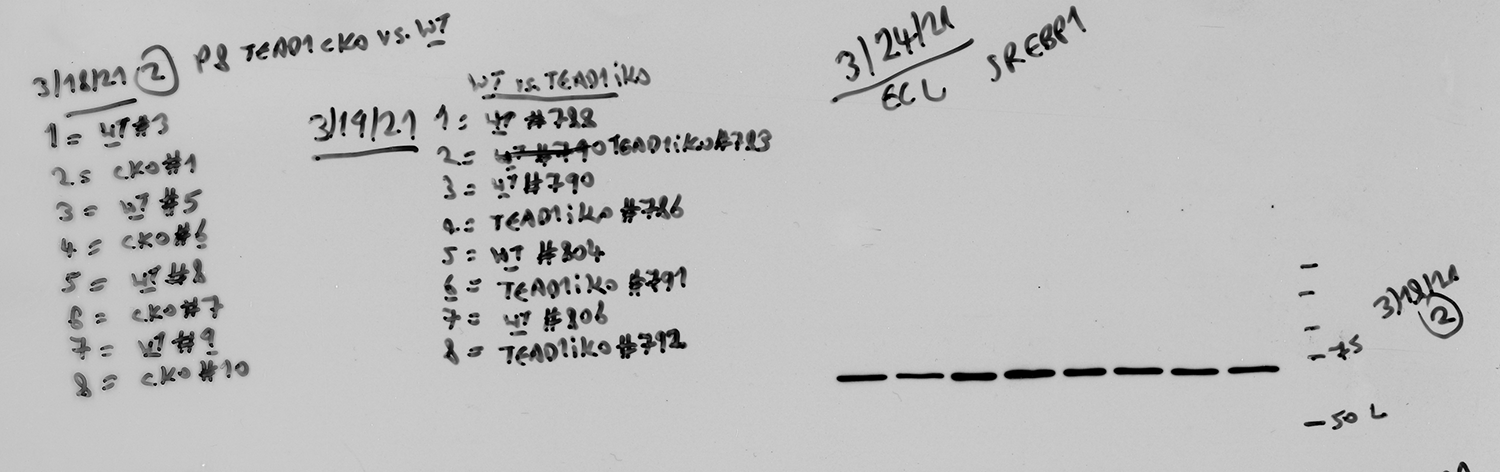

Supplement: Figure 5—source data 1. [file elife-87394-fig5-data1.zip › Fig 5 source data 1/Fig 5A P8 blots/P8 SREBP1/uncropped.tif]

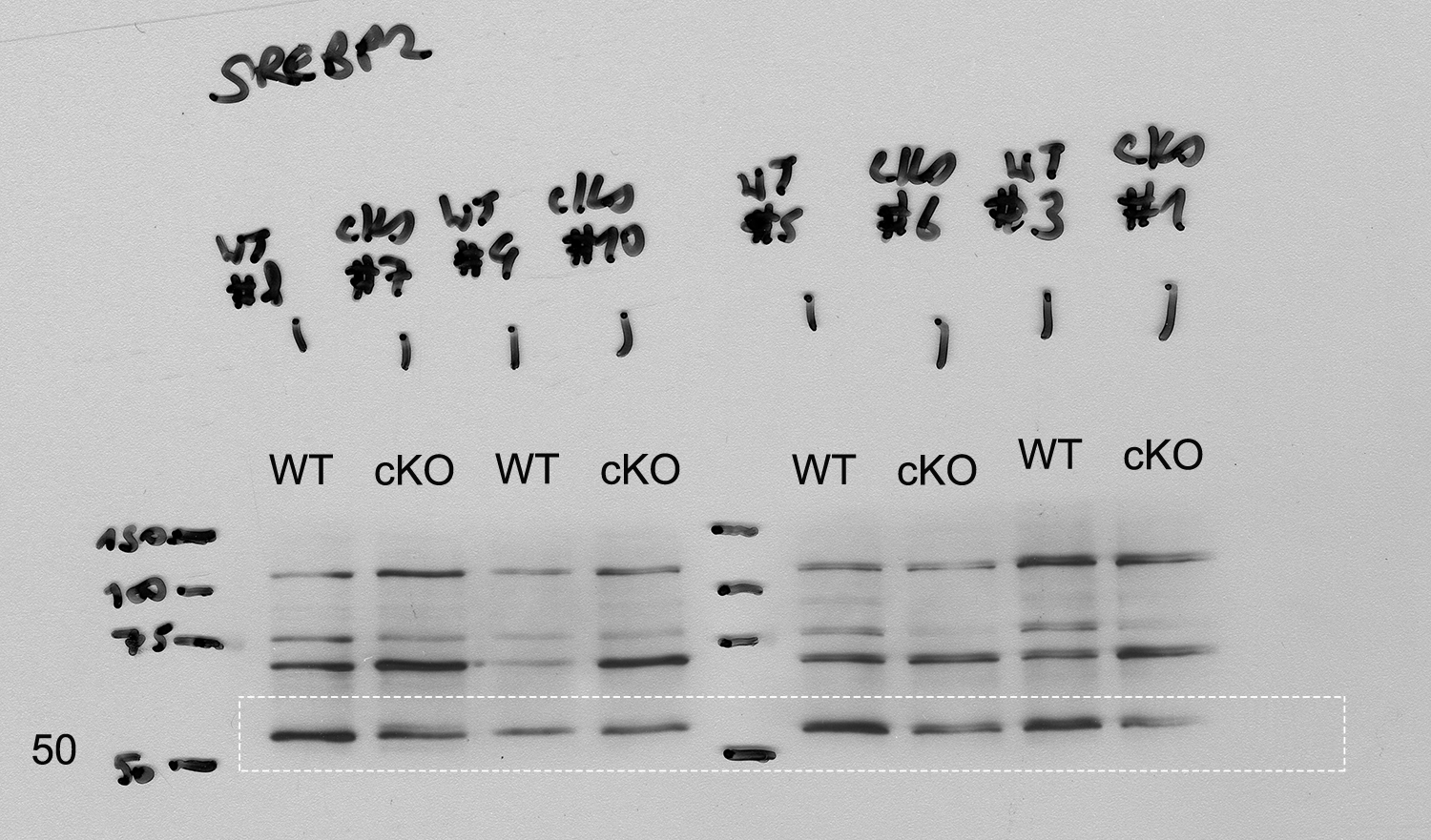

Supplement: Figure 5—source data 1. [file elife-87394-fig5-data1.zip › Fig 5 source data 1/Fig 5A P8 blots/P8 SREBP2/uncropped labeled.tif]

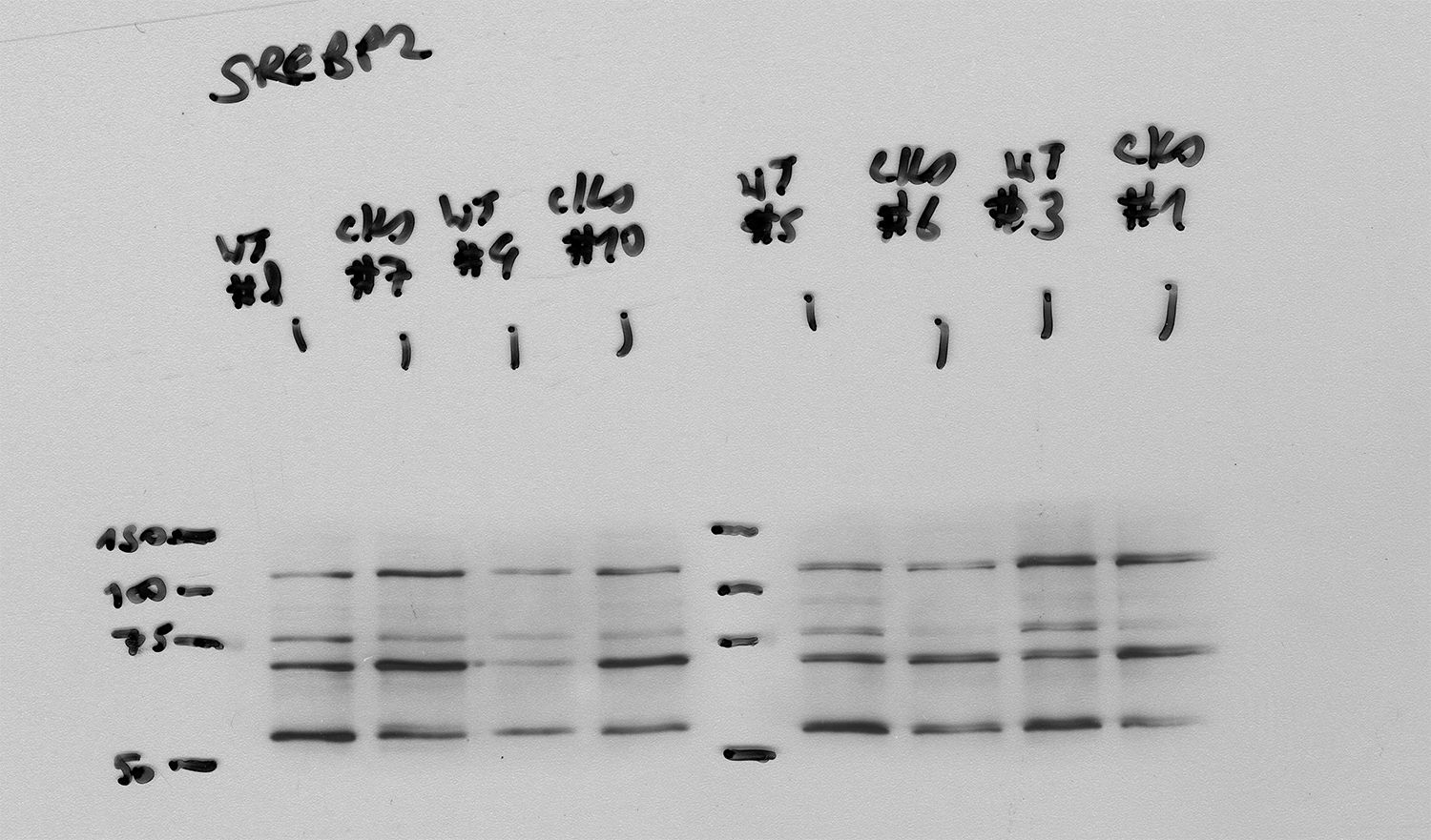

Supplement: Figure 5—source data 1. [file elife-87394-fig5-data1.zip › Fig 5 source data 1/Fig 5A P8 blots/P8 SREBP2/uncropped.tif]

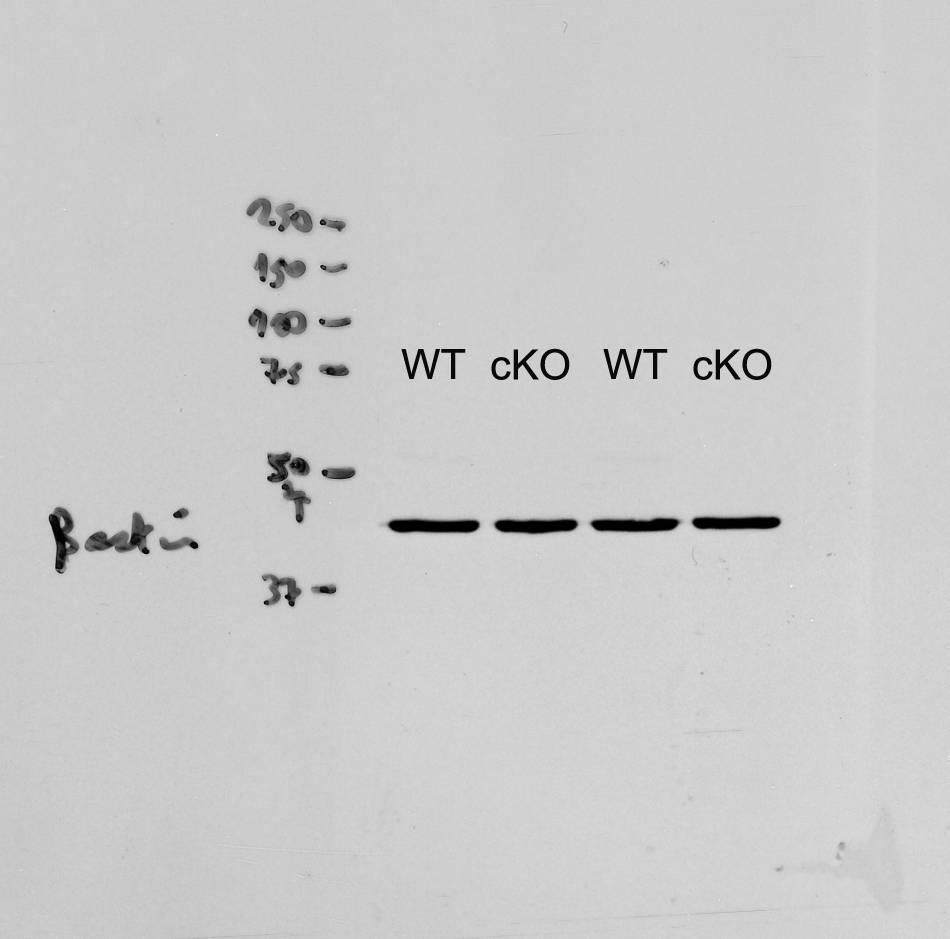

Supplement: Figure 5—source data 2. [file elife-87394-fig5-data2.zip › Fig 5 souce data 2/Fig 5A P40 blots/Tead1 cKO actin for HMGCR/uncropped labeled.tif]

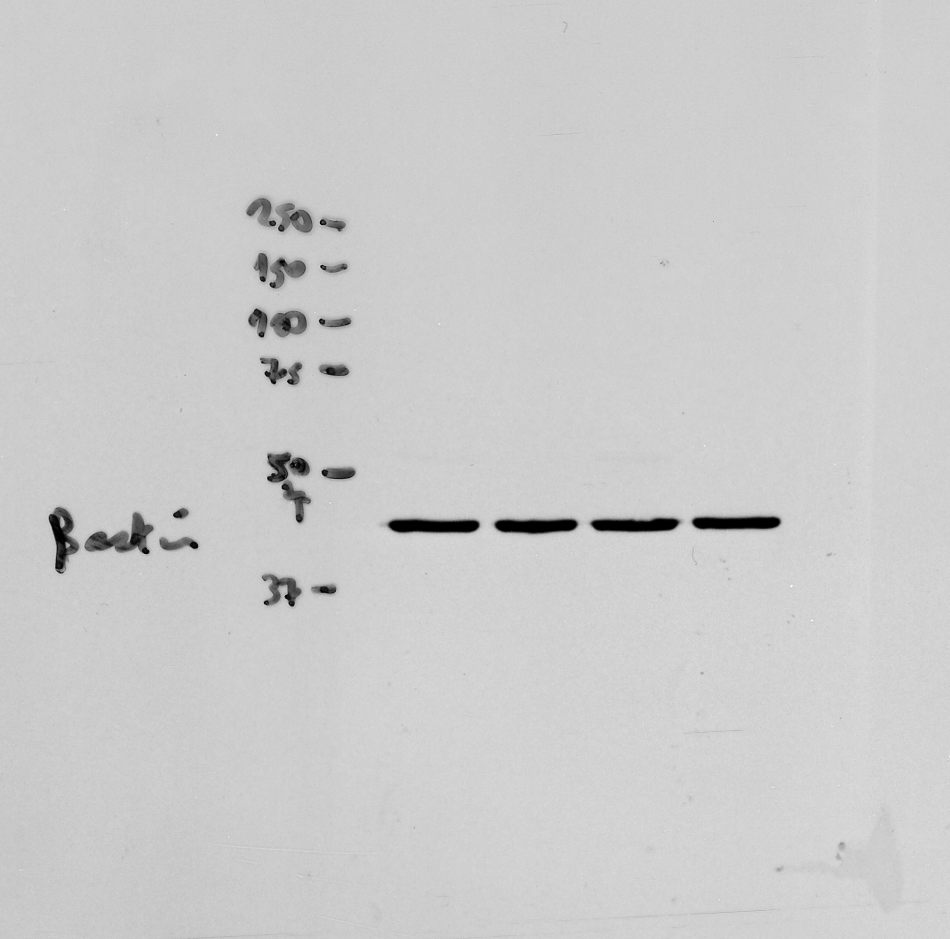

Supplement: Figure 5—source data 2. [file elife-87394-fig5-data2.zip › Fig 5 souce data 2/Fig 5A P40 blots/Tead1 cKO actin for HMGCR/uncropped.tif]

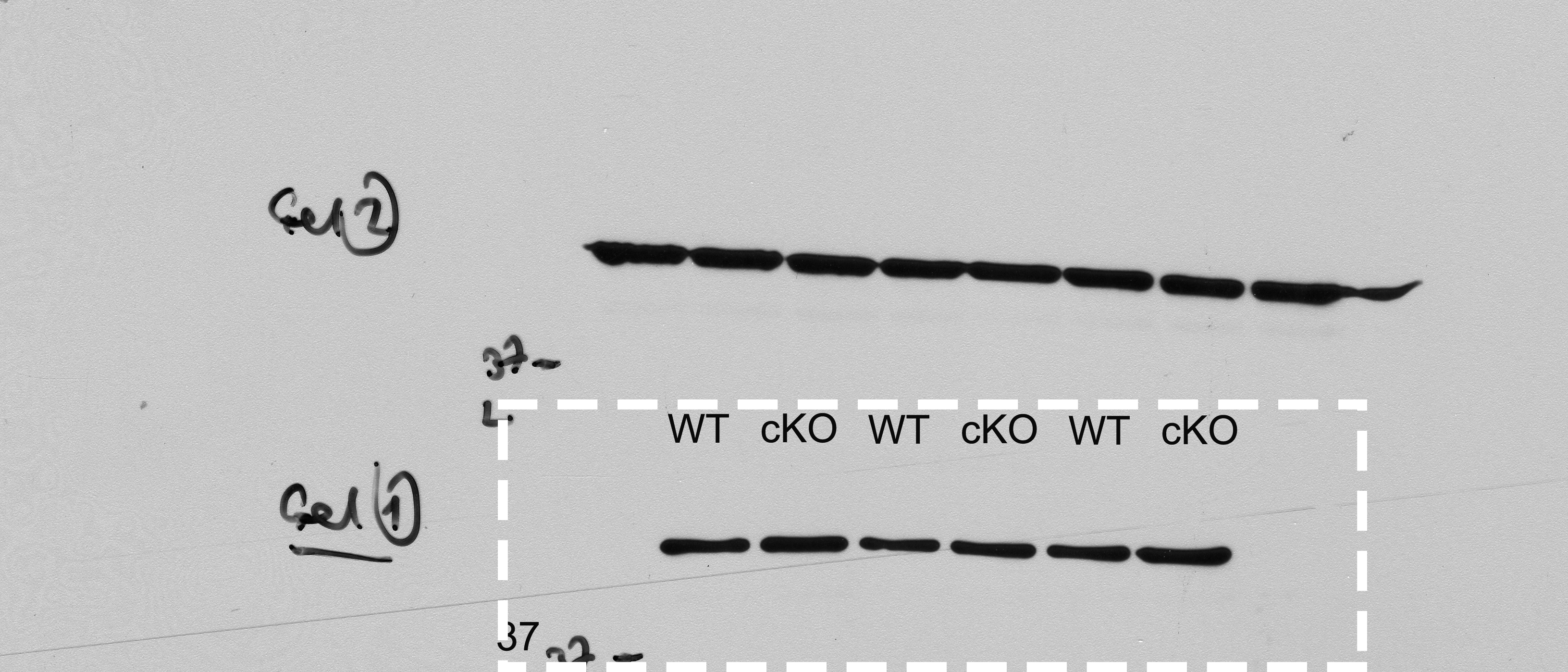

Supplement: Figure 5—source data 2. [file elife-87394-fig5-data2.zip › Fig 5 souce data 2/Fig 5A P40 blots/Tead1 cKO actin for SCD1/uncropped labeled.tif]

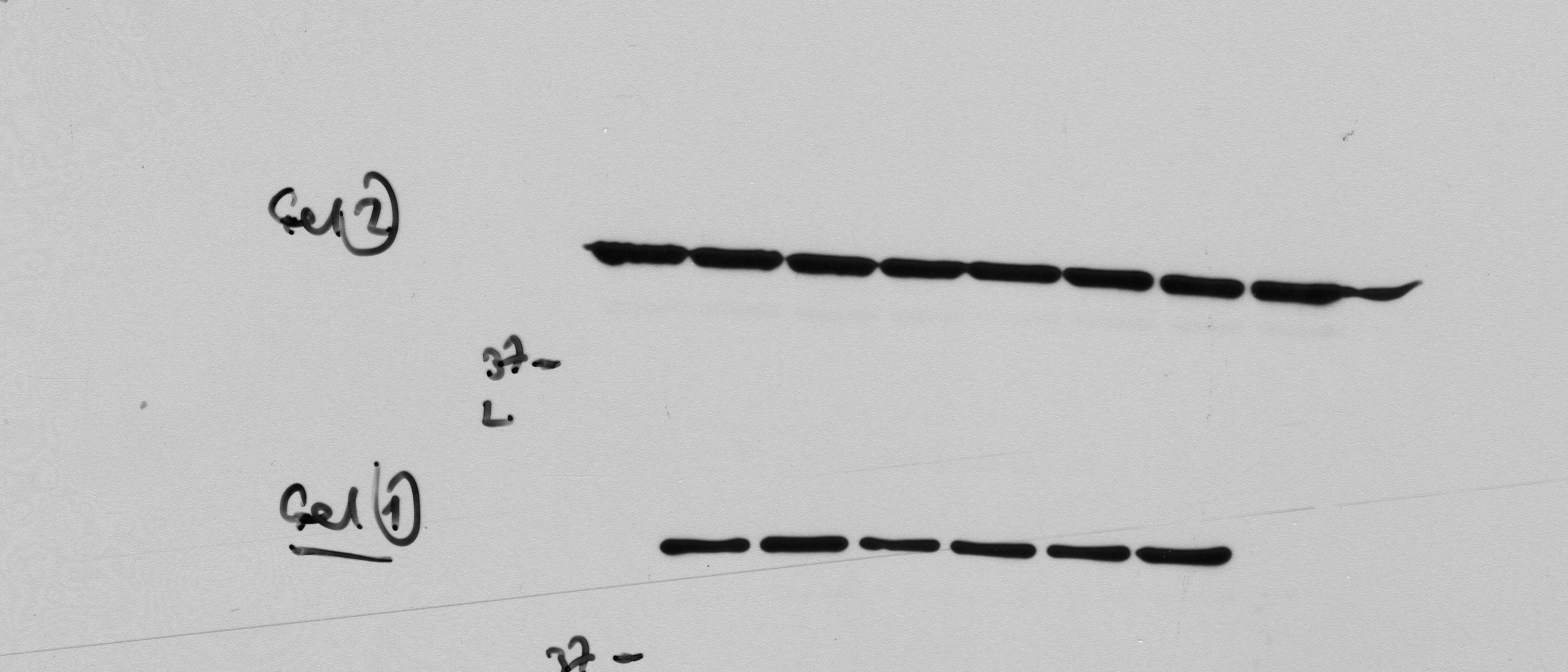

Supplement: Figure 5—source data 2. [file elife-87394-fig5-data2.zip › Fig 5 souce data 2/Fig 5A P40 blots/Tead1 cKO actin for SCD1/uncropped.tif]

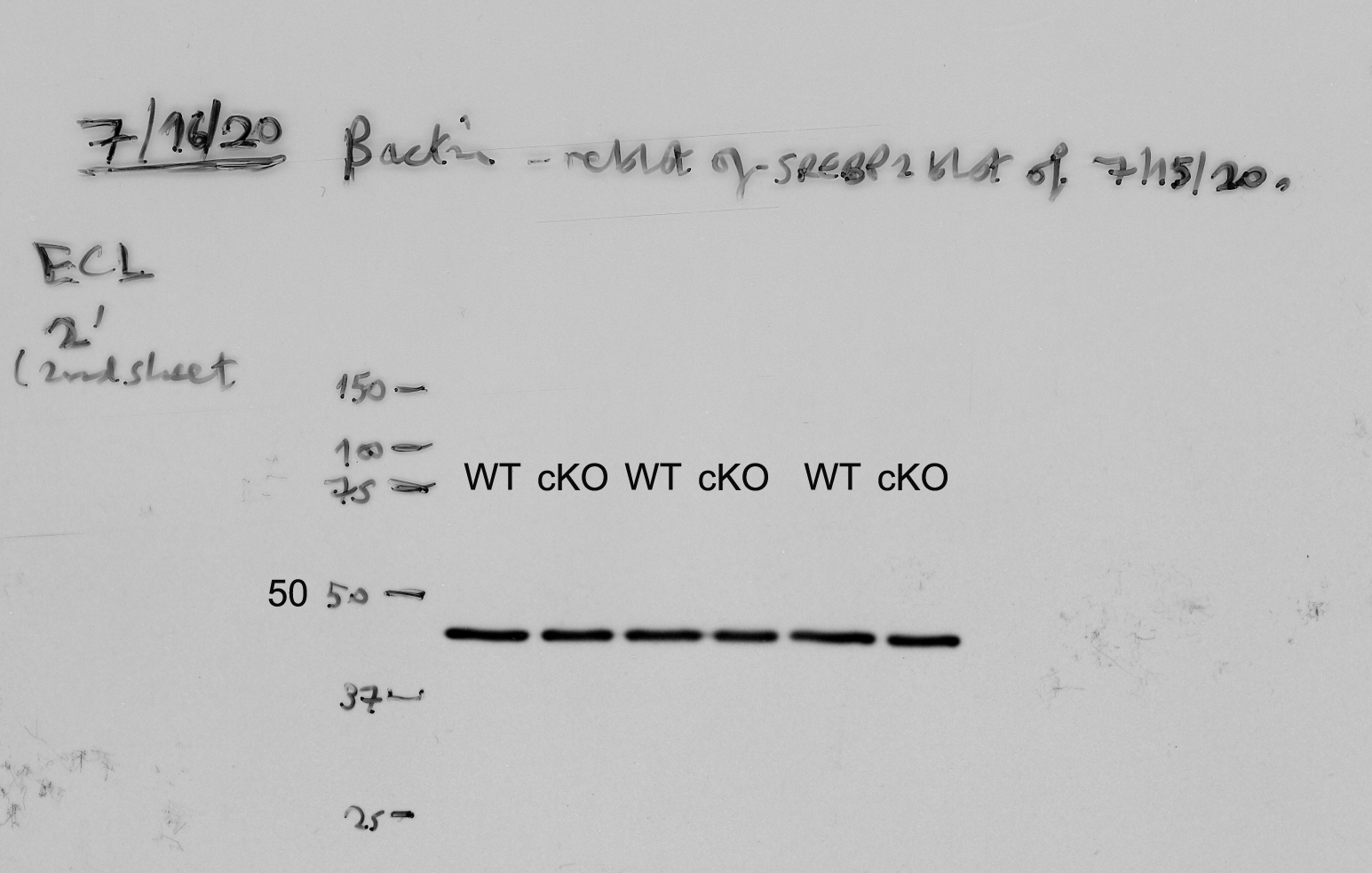

Supplement: Figure 5—source data 2. [file elife-87394-fig5-data2.zip › Fig 5 souce data 2/Fig 5A P40 blots/Tead1 cKO actin for SREBP2/uncropped labeled.tif]

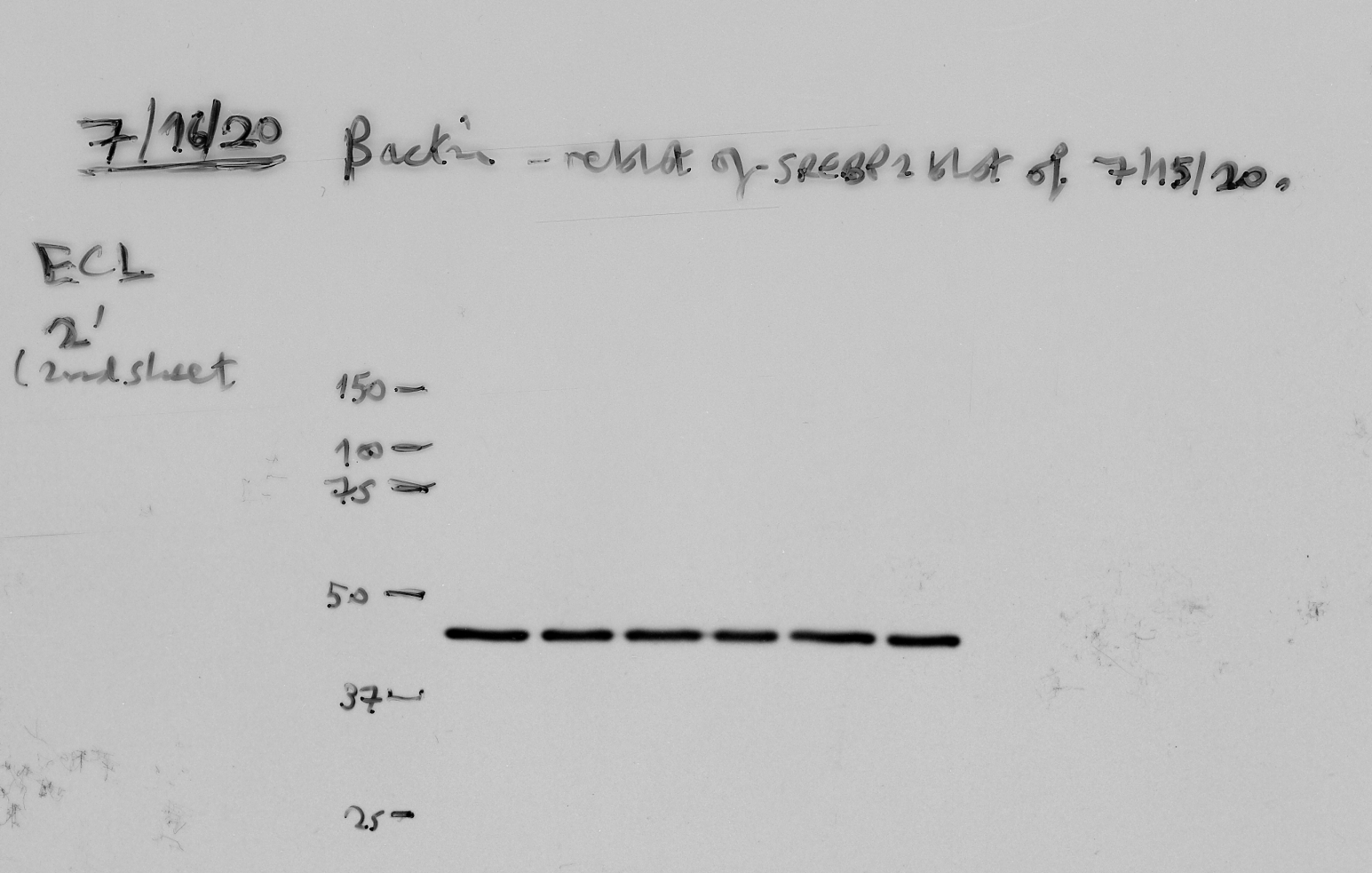

Supplement: Figure 5—source data 2. [file elife-87394-fig5-data2.zip › Fig 5 souce data 2/Fig 5A P40 blots/Tead1 cKO actin for SREBP2/uncropped.tif]

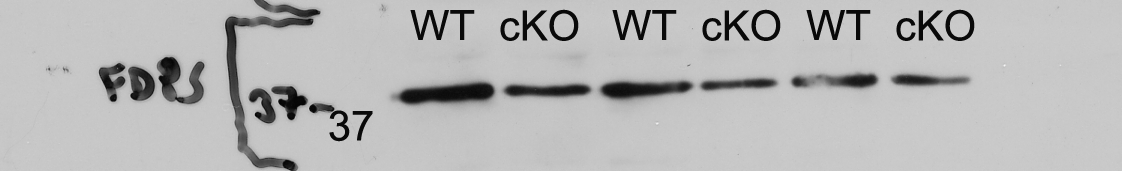

Supplement: Figure 5—source data 2. [file elife-87394-fig5-data2.zip › Fig 5 souce data 2/Fig 5A P40 blots/Tead1 cKO FDPS/uncropped labeled.tif]

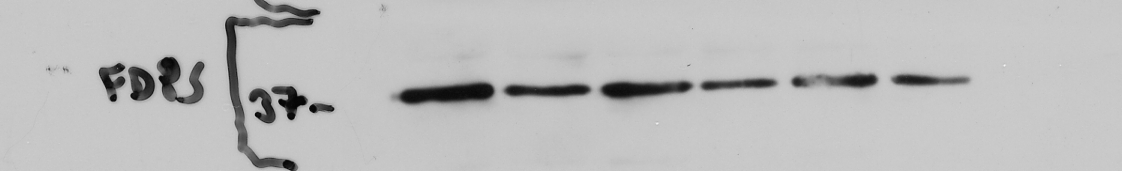

Supplement: Figure 5—source data 2. [file elife-87394-fig5-data2.zip › Fig 5 souce data 2/Fig 5A P40 blots/Tead1 cKO FDPS/uncropped.tif]

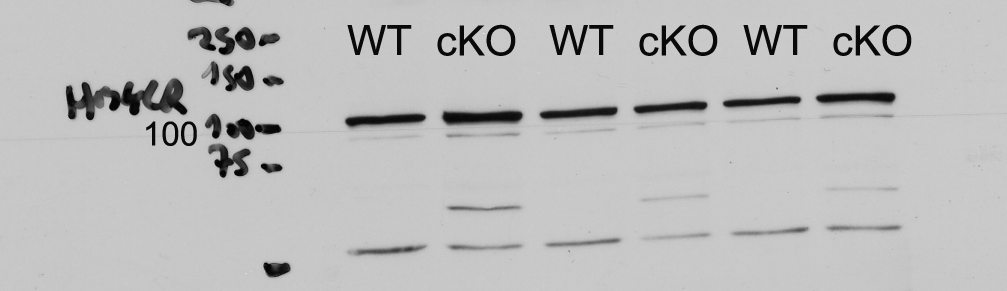

Supplement: Figure 5—source data 2. [file elife-87394-fig5-data2.zip › Fig 5 souce data 2/Fig 5A P40 blots/Tead1 cKO HMGCR/uncropped labeled.tif]

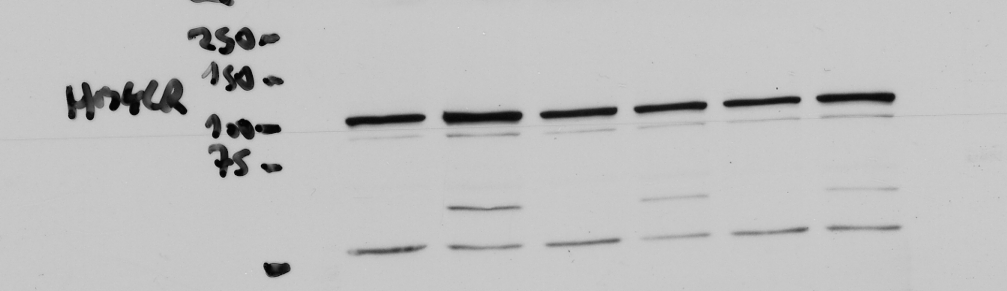

Supplement: Figure 5—source data 2. [file elife-87394-fig5-data2.zip › Fig 5 souce data 2/Fig 5A P40 blots/Tead1 cKO HMGCR/uncropped.tif]

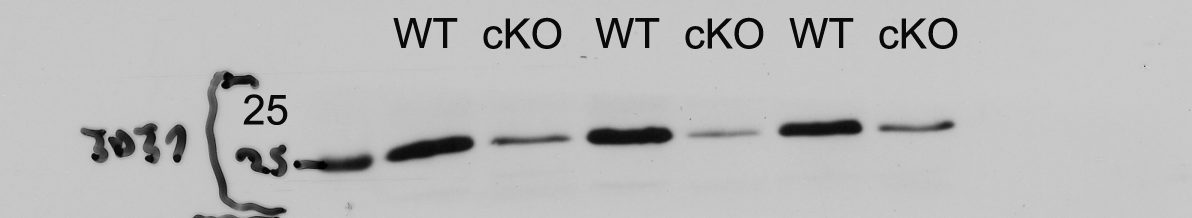

Supplement: Figure 5—source data 2. [file elife-87394-fig5-data2.zip › Fig 5 souce data 2/Fig 5A P40 blots/Tead1 cKO IDI1/uncropped labeled.tif]

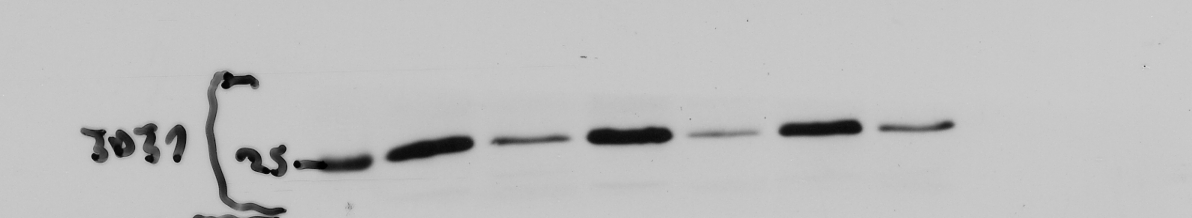

Supplement: Figure 5—source data 2. [file elife-87394-fig5-data2.zip › Fig 5 souce data 2/Fig 5A P40 blots/Tead1 cKO IDI1/uncropped.tif]

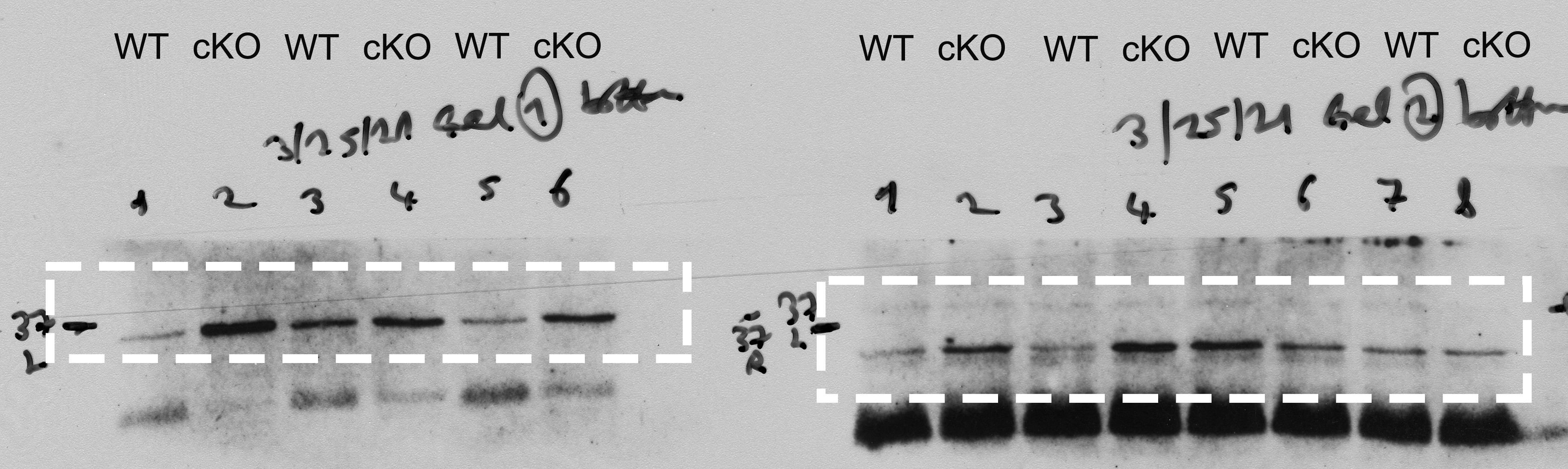

Supplement: Figure 5—source data 2. [file elife-87394-fig5-data2.zip › Fig 5 souce data 2/Fig 5A P40 blots/Tead1 cKO SCD1/uncropped labeled.tif]

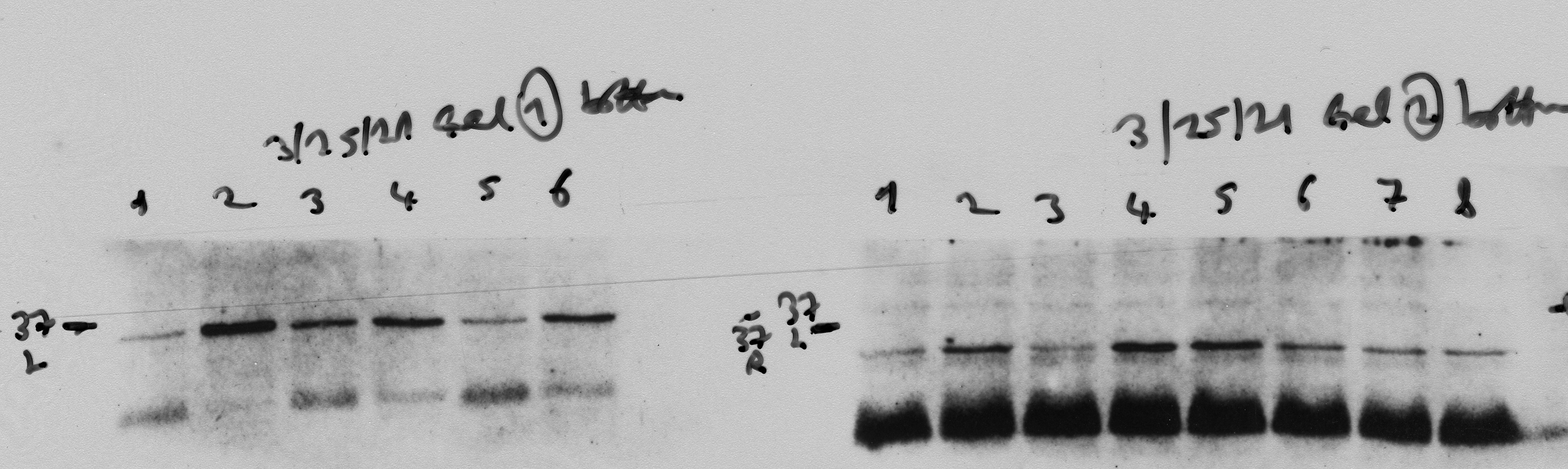

Supplement: Figure 5—source data 2. [file elife-87394-fig5-data2.zip › Fig 5 souce data 2/Fig 5A P40 blots/Tead1 cKO SCD1/uncropped.tif]

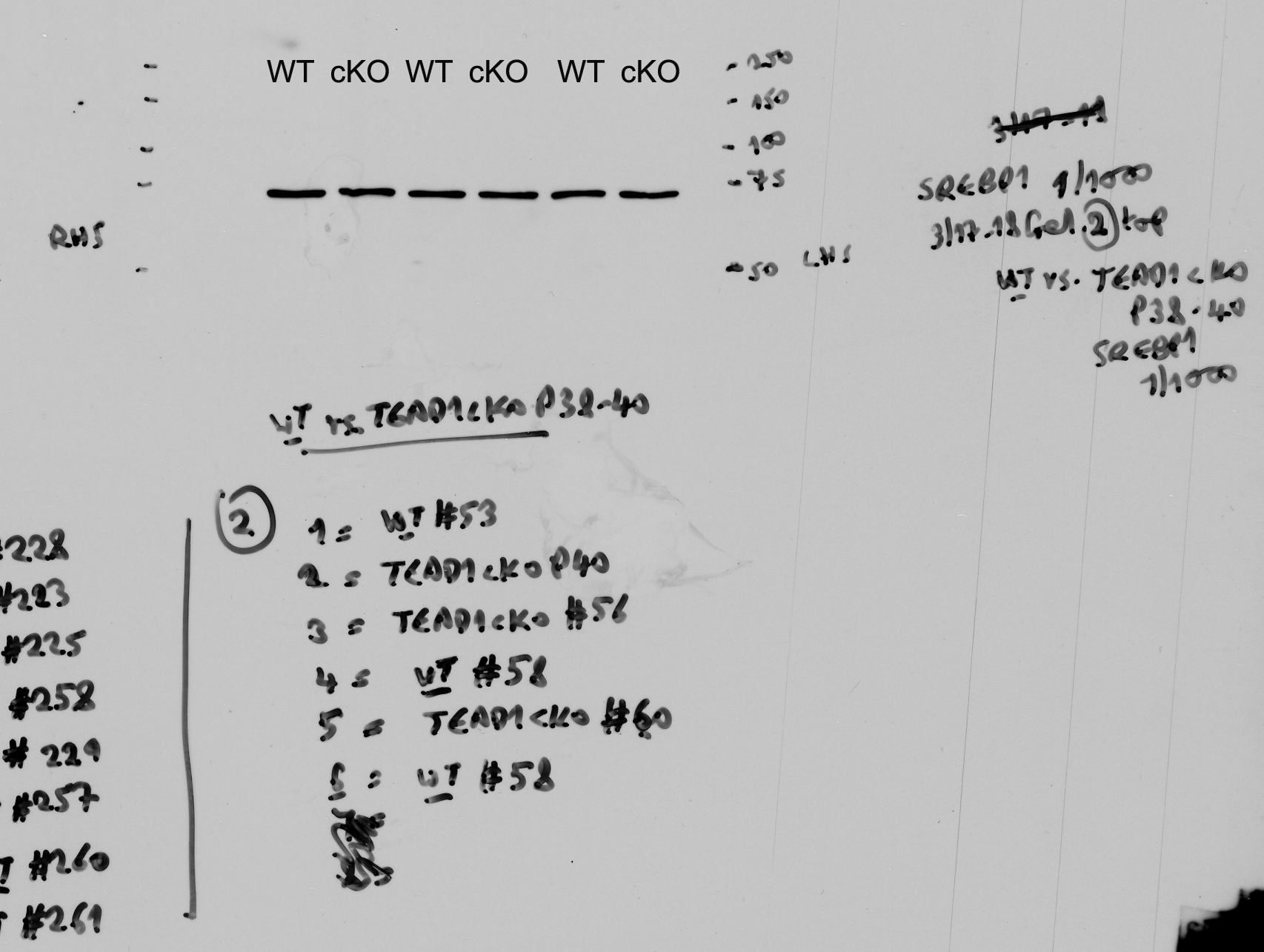

Supplement: Figure 5—source data 2. [file elife-87394-fig5-data2.zip › Fig 5 souce data 2/Fig 5A P40 blots/Tead1 cKO SREBP1/uncropped labeled.tif]

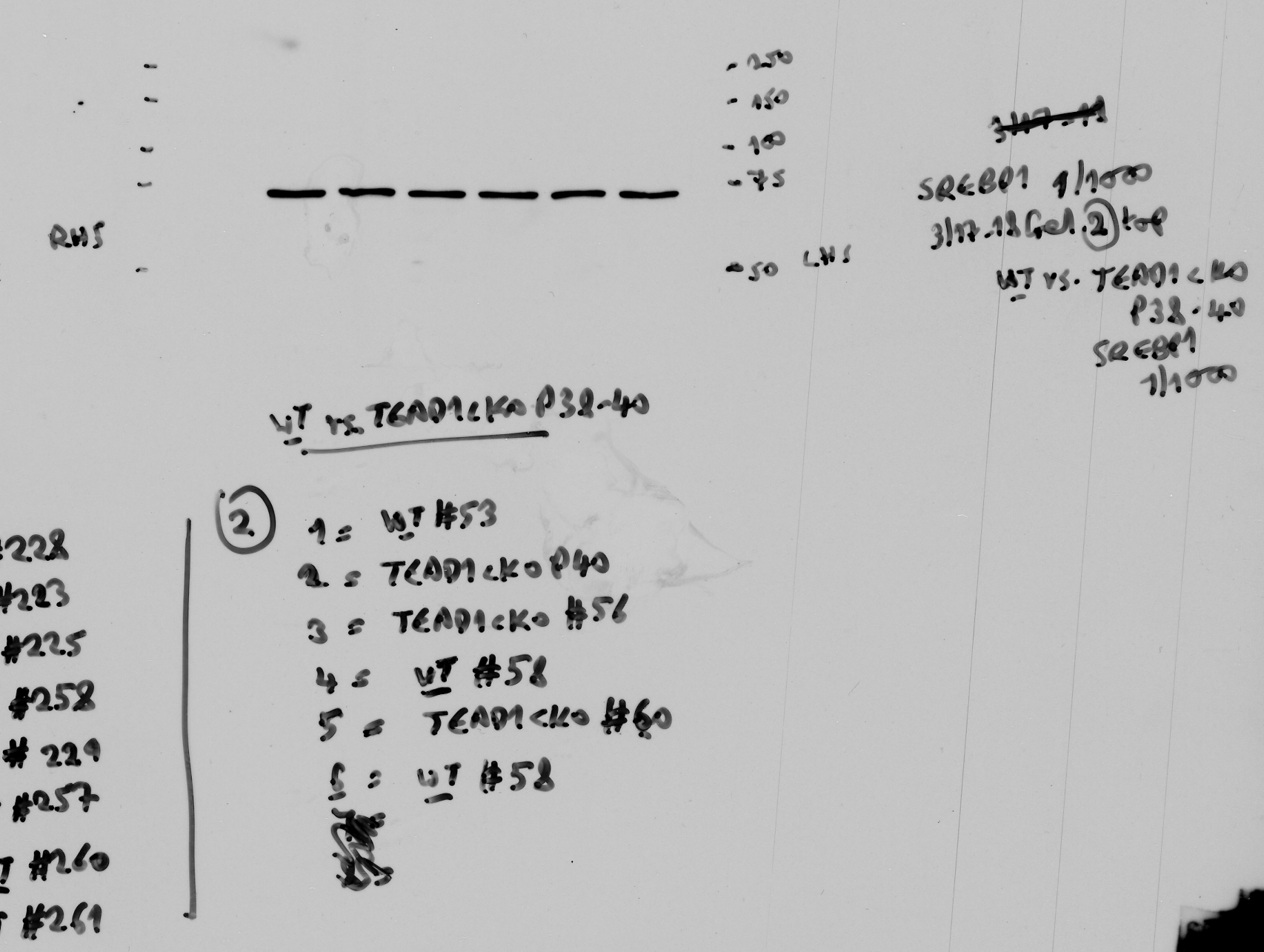

Supplement: Figure 5—source data 2. [file elife-87394-fig5-data2.zip › Fig 5 souce data 2/Fig 5A P40 blots/Tead1 cKO SREBP1/uncropped.tif]

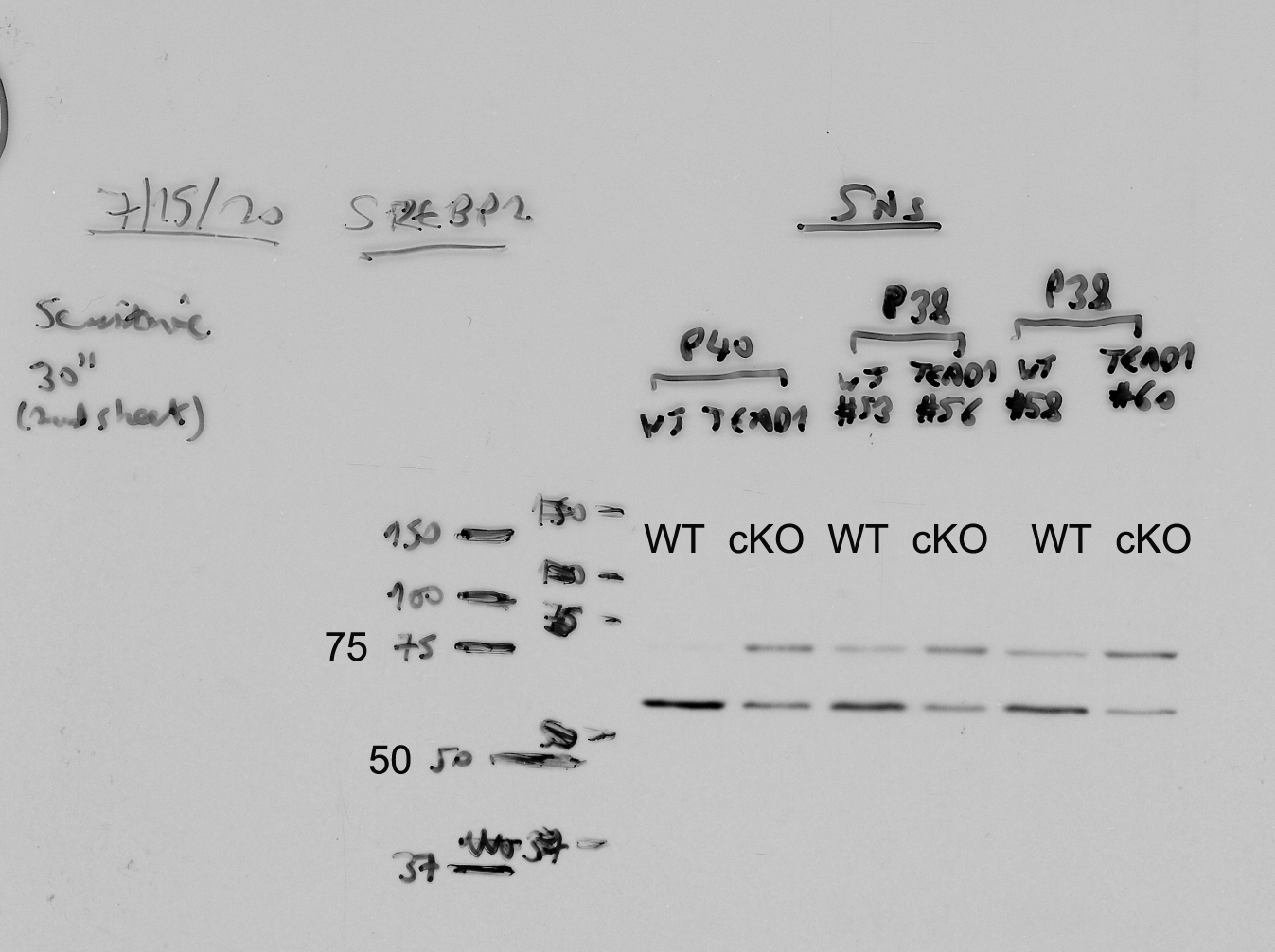

Supplement: Figure 5—source data 2. [file elife-87394-fig5-data2.zip › Fig 5 souce data 2/Fig 5A P40 blots/Tead1 cKO SREBP2/uncropped labeled.tif]

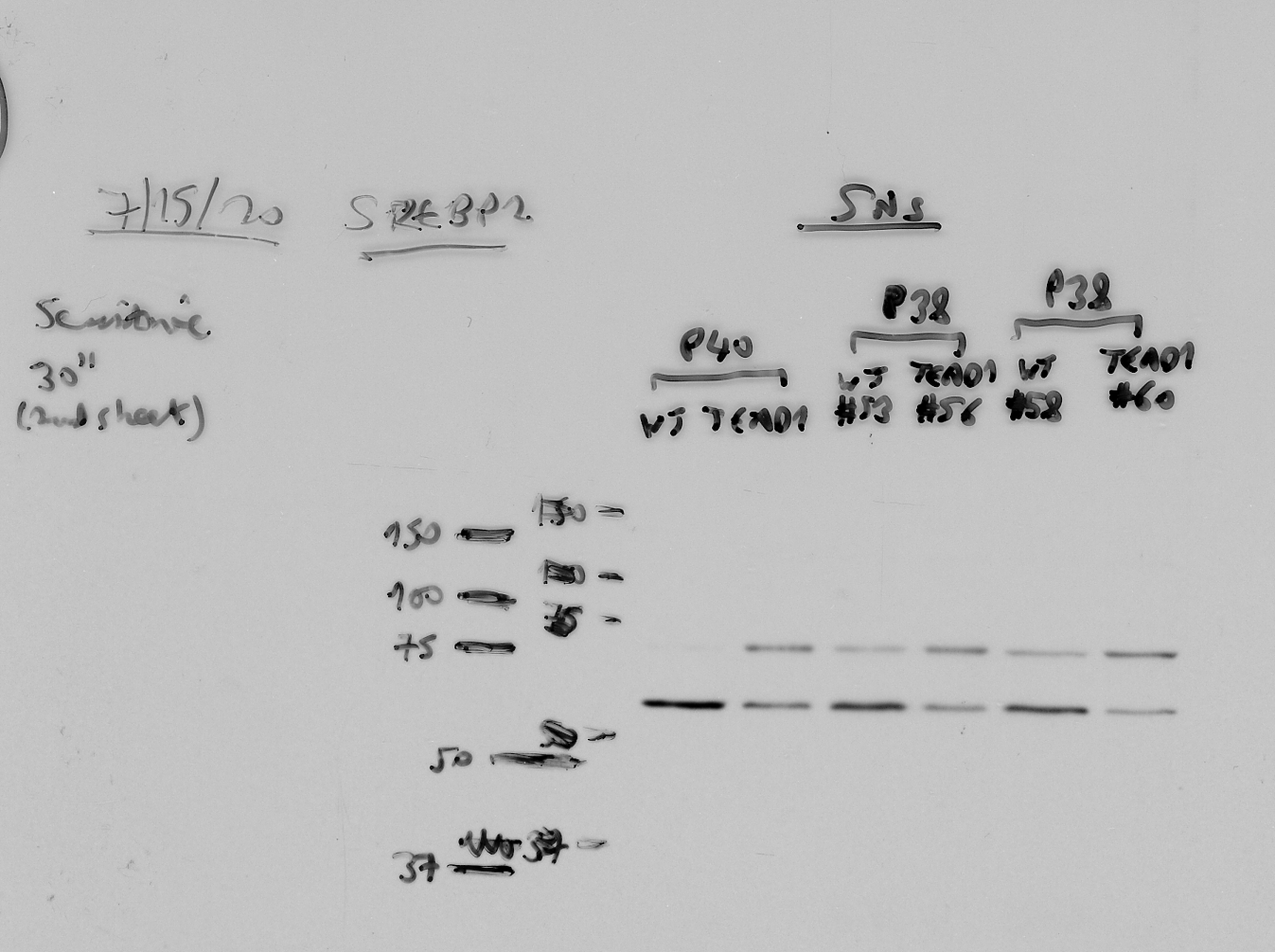

Supplement: Figure 5—source data 2. [file elife-87394-fig5-data2.zip › Fig 5 souce data 2/Fig 5A P40 blots/Tead1 cKO SREBP2/uncropped.tif]

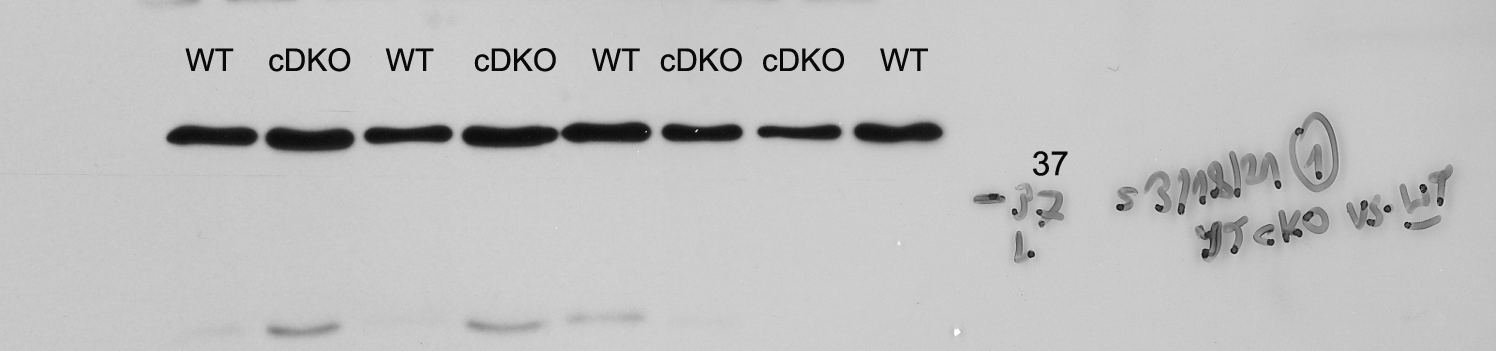

Supplement: Figure 5—source data 2. [file elife-87394-fig5-data2.zip › Fig 5 souce data 2/Fig 5B P60 blots and prism files/YT beta actin for SREBP1/uncropped labeled.tif]

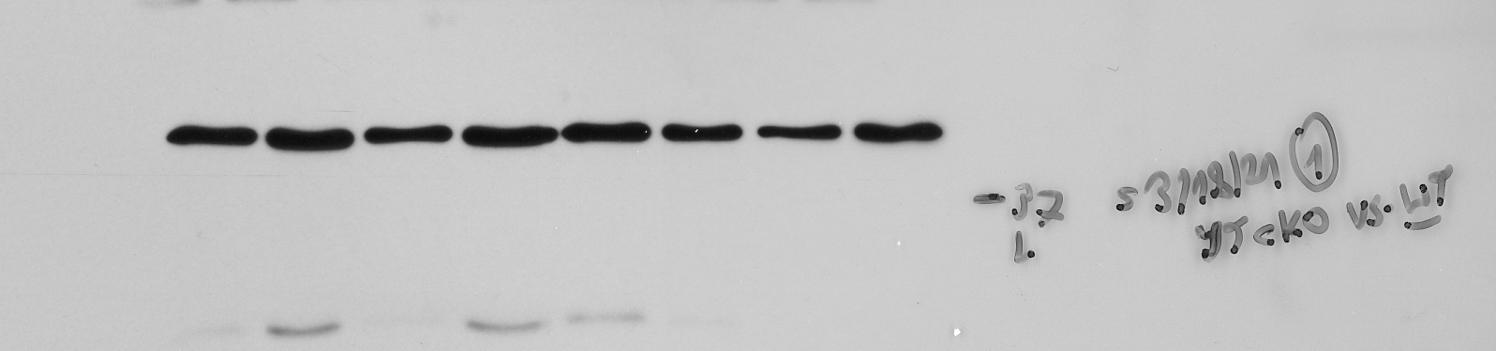

Supplement: Figure 5—source data 2. [file elife-87394-fig5-data2.zip › Fig 5 souce data 2/Fig 5B P60 blots and prism files/YT beta actin for SREBP1/uncropped.tif]

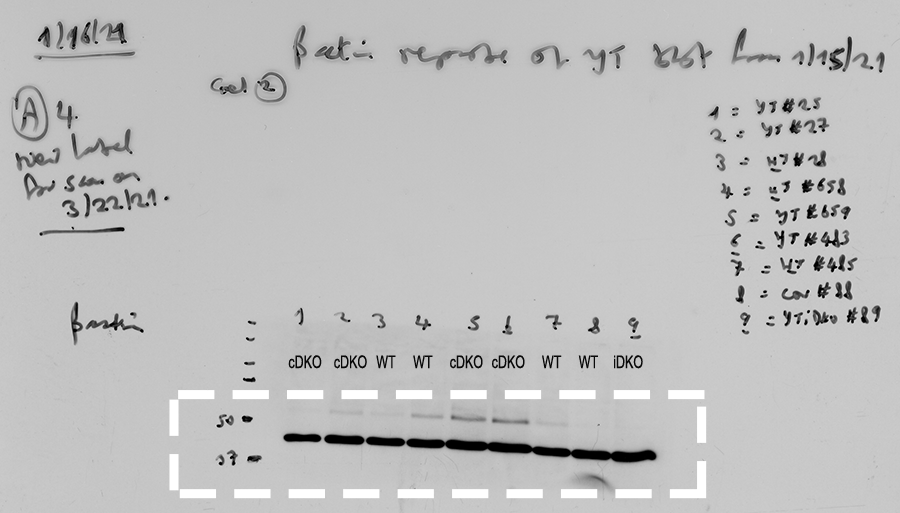

Supplement: Figure 5—source data 2. [file elife-87394-fig5-data2.zip › Fig 5 souce data 2/Fig 5B P60 blots and prism files/YT cDKO actin for HMGCR/uncropped labeled.tif]

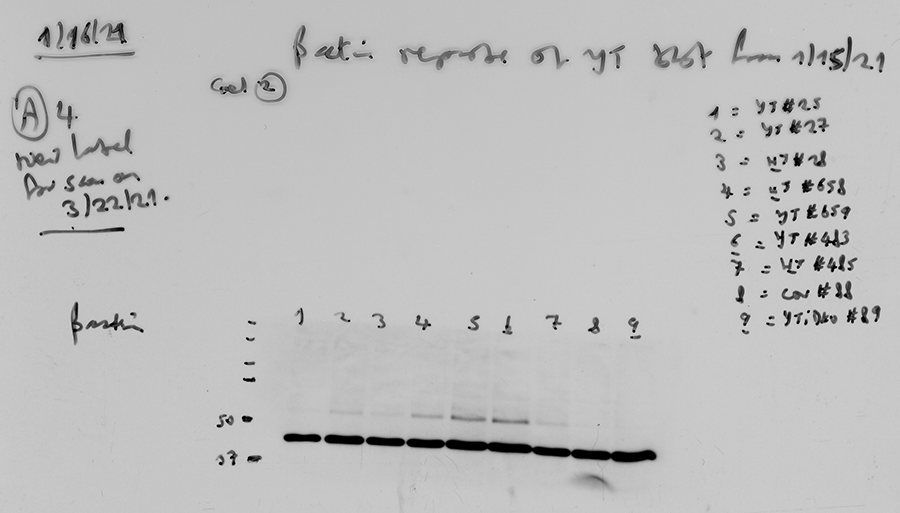

Supplement: Figure 5—source data 2. [file elife-87394-fig5-data2.zip › Fig 5 souce data 2/Fig 5B P60 blots and prism files/YT cDKO actin for HMGCR/uncropped.tif]

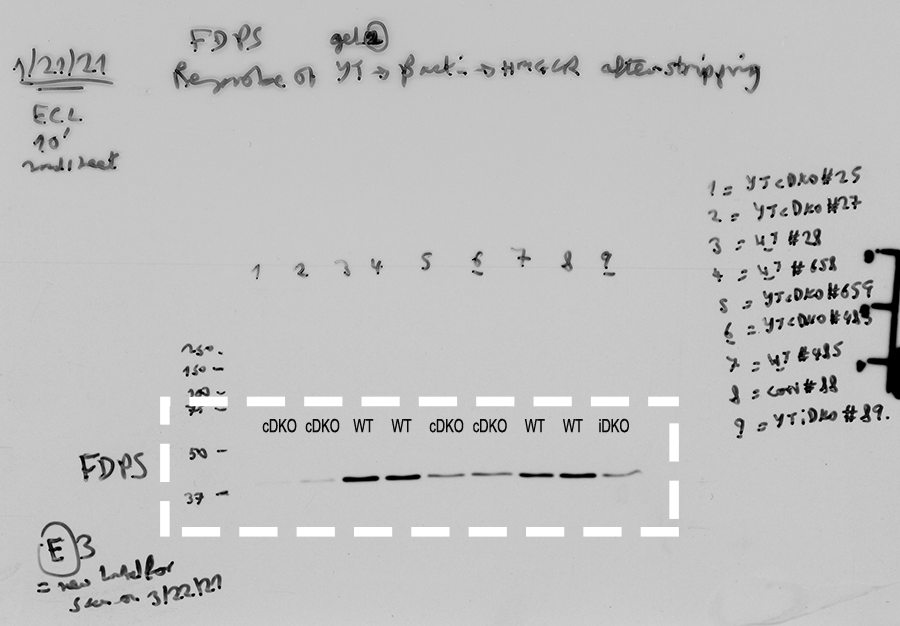

Supplement: Figure 5—source data 2. [file elife-87394-fig5-data2.zip › Fig 5 souce data 2/Fig 5B P60 blots and prism files/YT cDKO FDPS/uncropped labeled.tif]

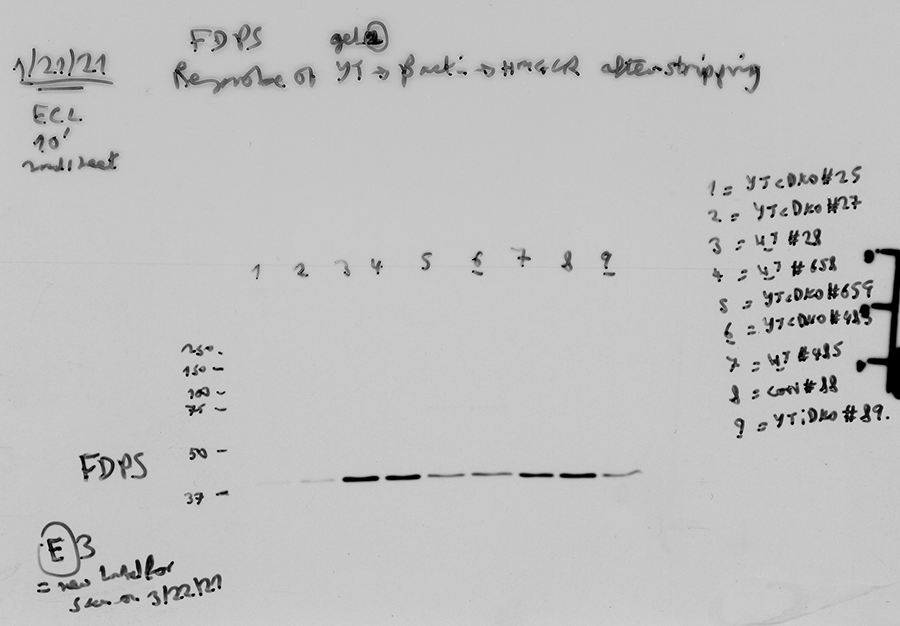

Supplement: Figure 5—source data 2. [file elife-87394-fig5-data2.zip › Fig 5 souce data 2/Fig 5B P60 blots and prism files/YT cDKO FDPS/uncropped.tif]
