# Supplementary figures and images for: TEAD1 is crucial for developmental myelination, Remak bundles, and functional regeneration of peripheral nerves (part 2 of 2)
Source: eLife. 2024 Mar 8;13:e87394. doi: 10.7554/eLife.87394 (PMC10959528; doi:10.7554/eLife.87394)

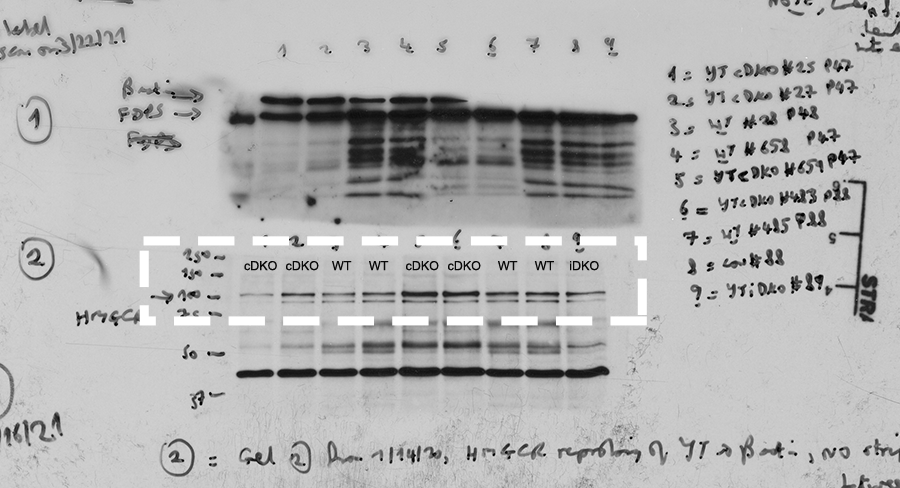

Supplement: Figure 5—source data 2. [file elife-87394-fig5-data2.zip › Fig 5 souce data 2/Fig 5B P60 blots and prism files/YT cDKO HMGCR/uncropped labeled.tif]

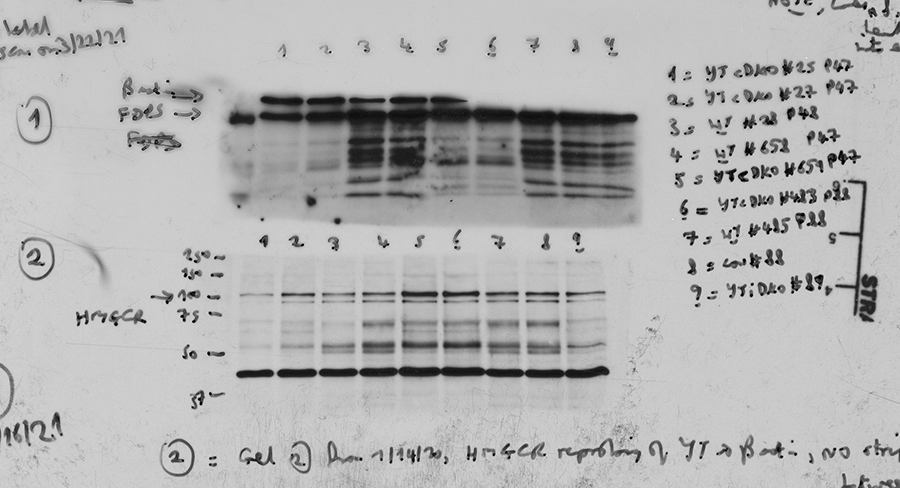

Supplement: Figure 5—source data 2. [file elife-87394-fig5-data2.zip › Fig 5 souce data 2/Fig 5B P60 blots and prism files/YT cDKO HMGCR/uncropped.tif]

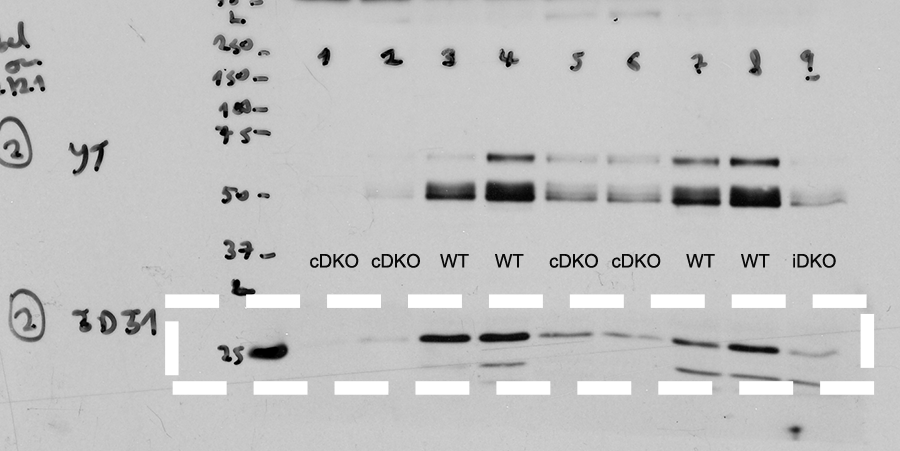

Supplement: Figure 5—source data 2. [file elife-87394-fig5-data2.zip › Fig 5 souce data 2/Fig 5B P60 blots and prism files/YT cDKO IDI1/uncropped labeled.tif]

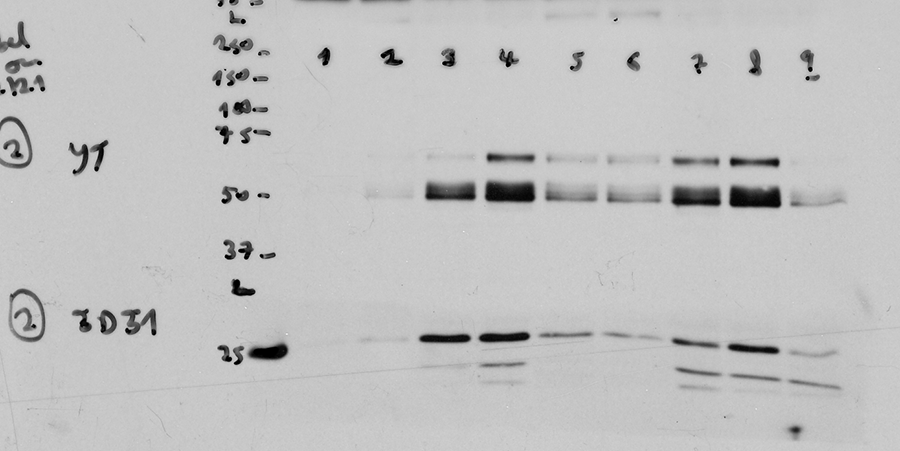

Supplement: Figure 5—source data 2. [file elife-87394-fig5-data2.zip › Fig 5 souce data 2/Fig 5B P60 blots and prism files/YT cDKO IDI1/uncropped.tif]

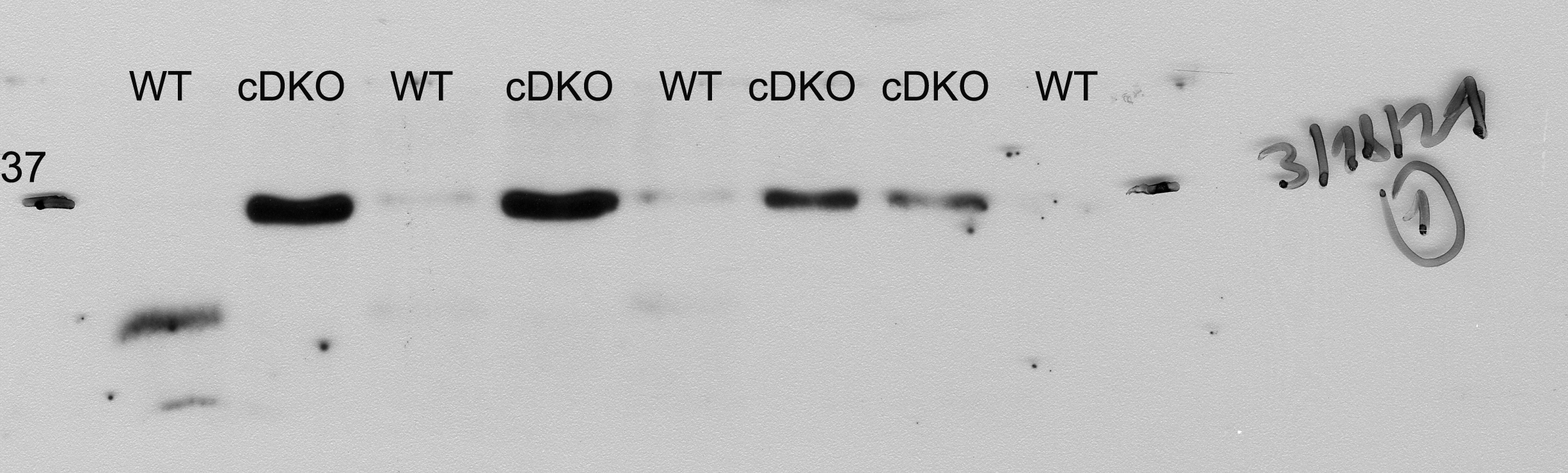

Supplement: Figure 5—source data 2. [file elife-87394-fig5-data2.zip › Fig 5 souce data 2/Fig 5B P60 blots and prism files/YT cDKO SCD1/uncropped labeled.tif]

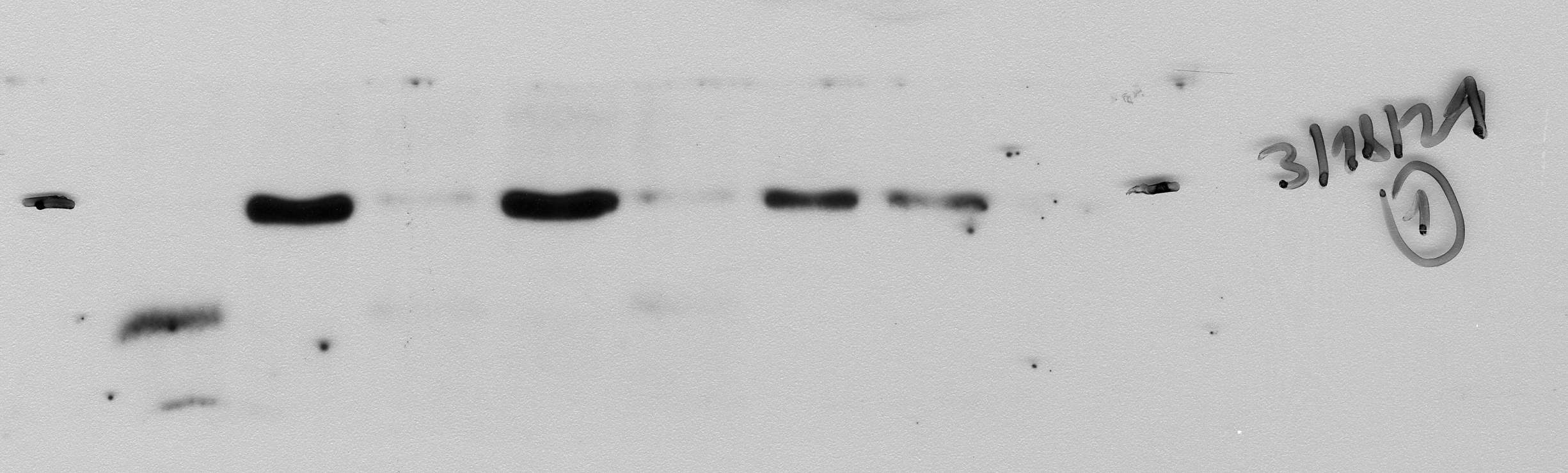

Supplement: Figure 5—source data 2. [file elife-87394-fig5-data2.zip › Fig 5 souce data 2/Fig 5B P60 blots and prism files/YT cDKO SCD1/uncropped.tif]

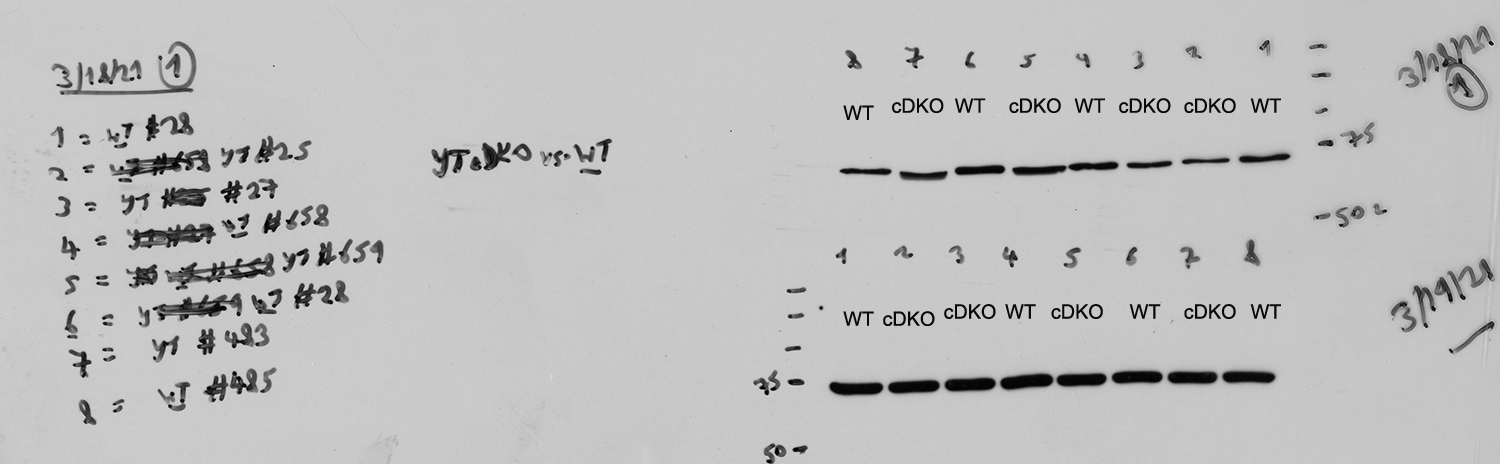

Supplement: Figure 5—source data 2. [file elife-87394-fig5-data2.zip › Fig 5 souce data 2/Fig 5B P60 blots and prism files/YT cDKO SREBP1/uncropped labeled.tif]

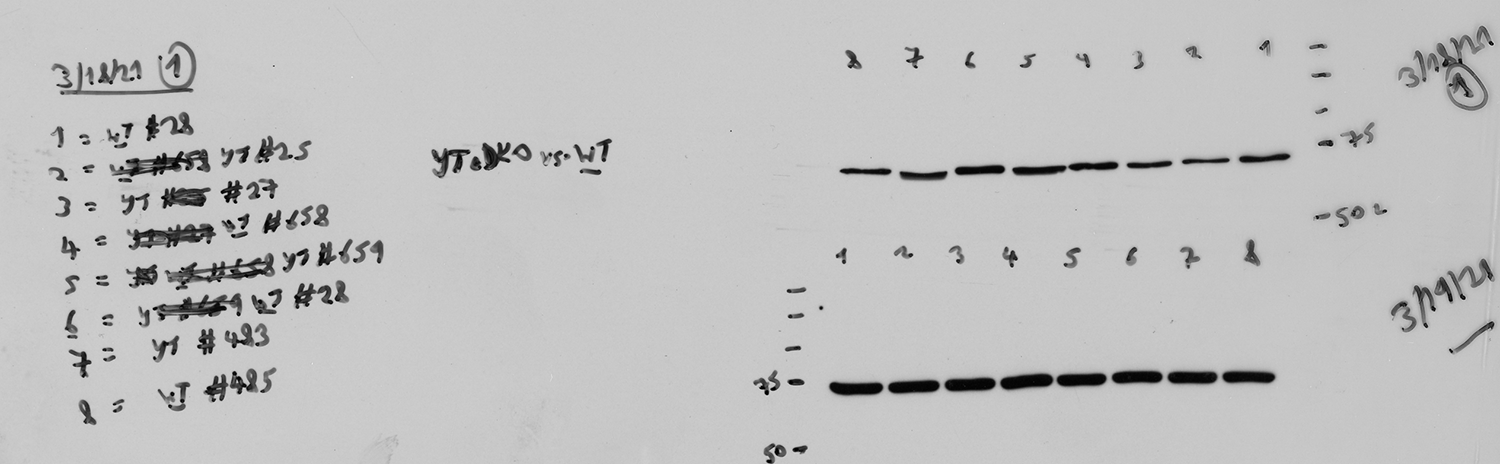

Supplement: Figure 5—source data 2. [file elife-87394-fig5-data2.zip › Fig 5 souce data 2/Fig 5B P60 blots and prism files/YT cDKO SREBP1/uncropped.tif]

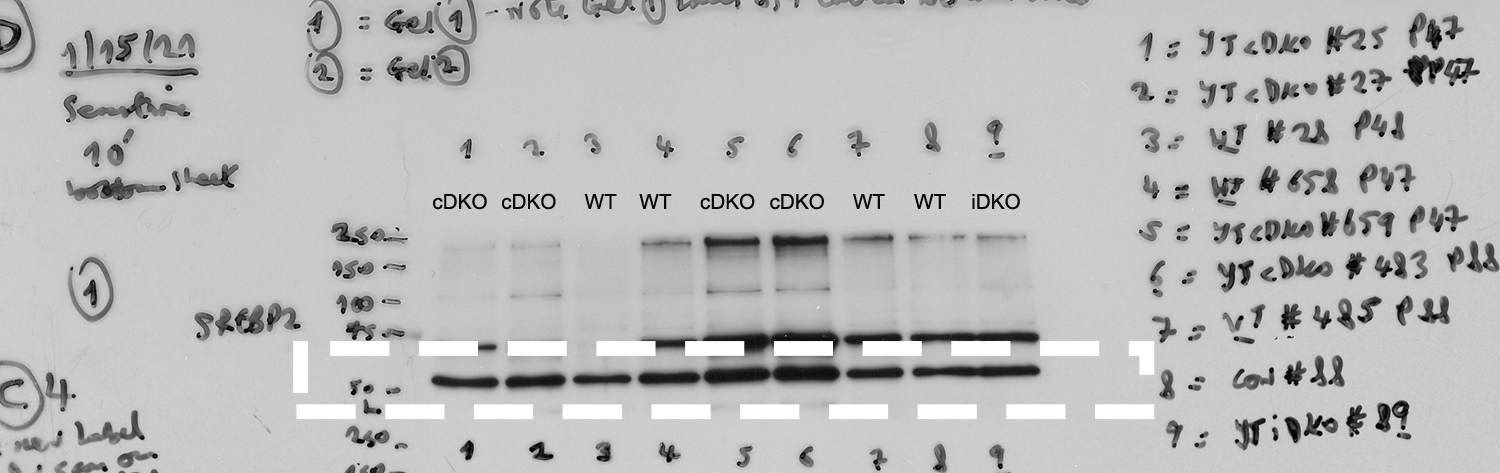

Supplement: Figure 5—source data 2. [file elife-87394-fig5-data2.zip › Fig 5 souce data 2/Fig 5B P60 blots and prism files/YT cDKO SREBP2/uncropped labeled.tif]

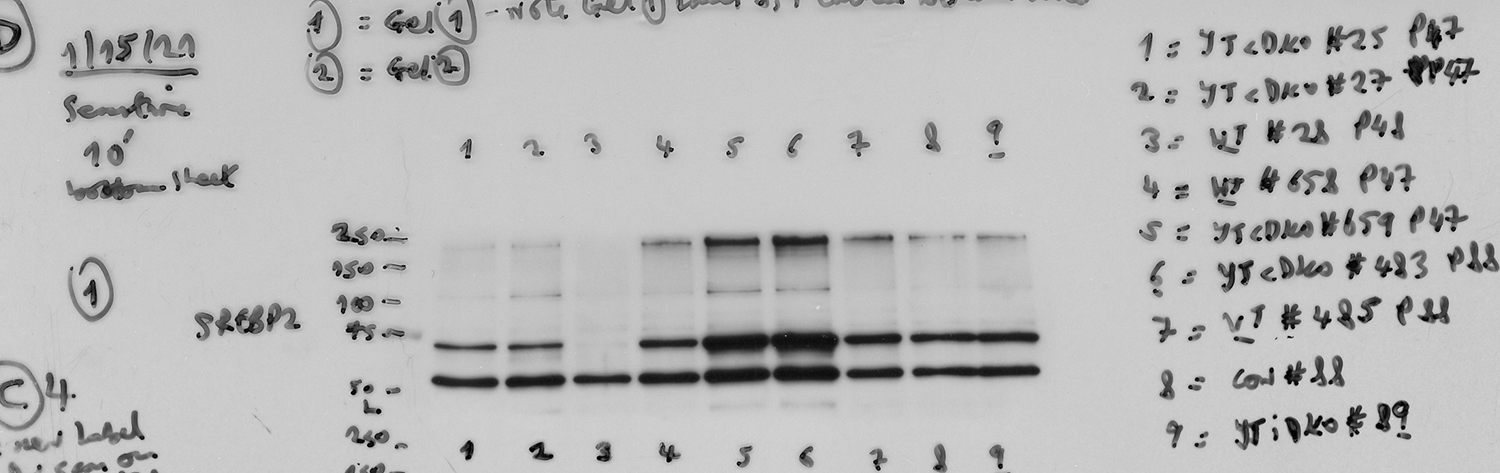

Supplement: Figure 5—source data 2. [file elife-87394-fig5-data2.zip › Fig 5 souce data 2/Fig 5B P60 blots and prism files/YT cDKO SREBP2/uncropped.tif]

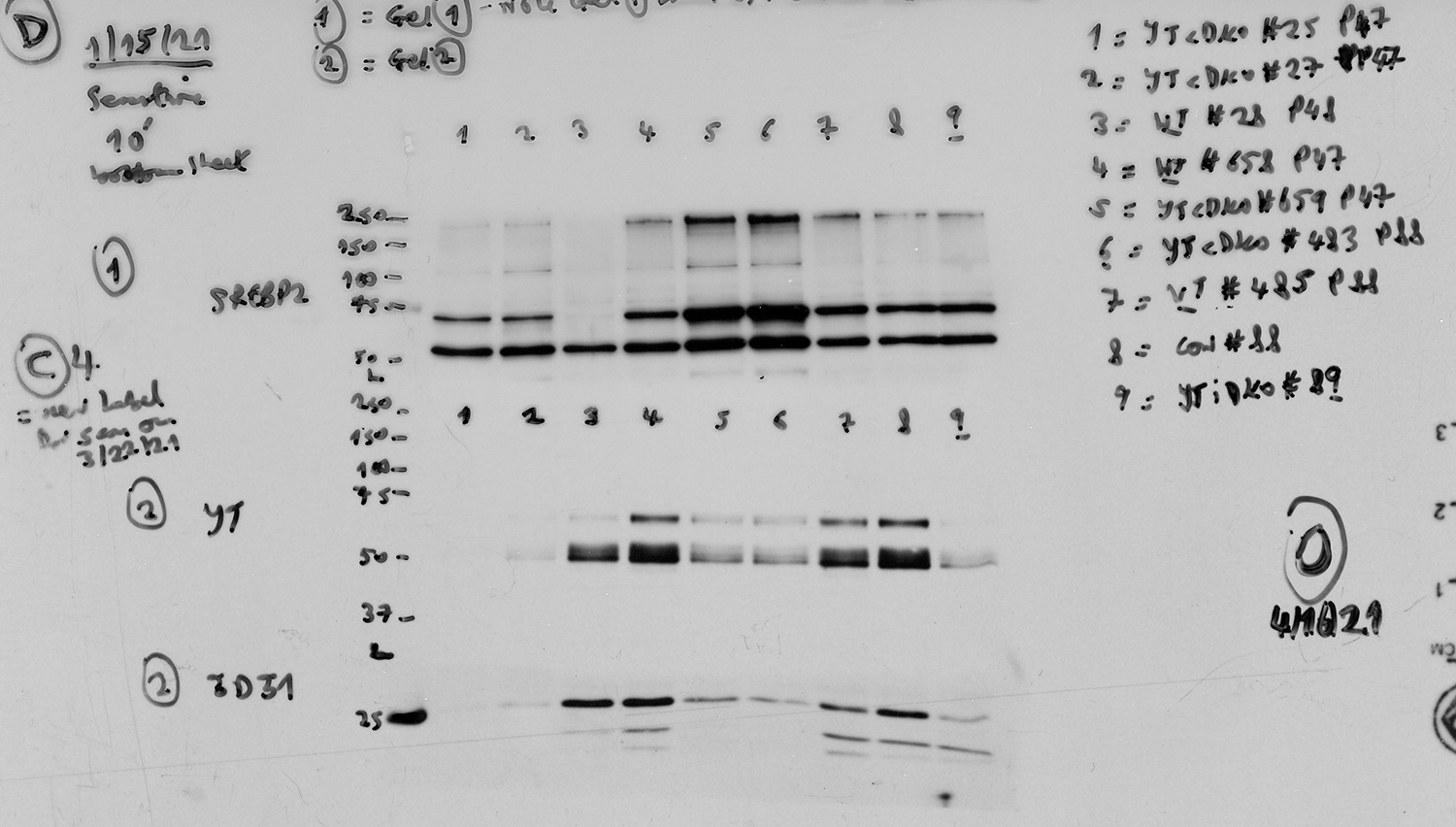

Supplement: Figure 5—source data 2. [file elife-87394-fig5-data2.zip › Fig 5 souce data 2/Fig 5B P60 blots and prism files/YT cDKO Yap and Taz/uncropped 1.tif]

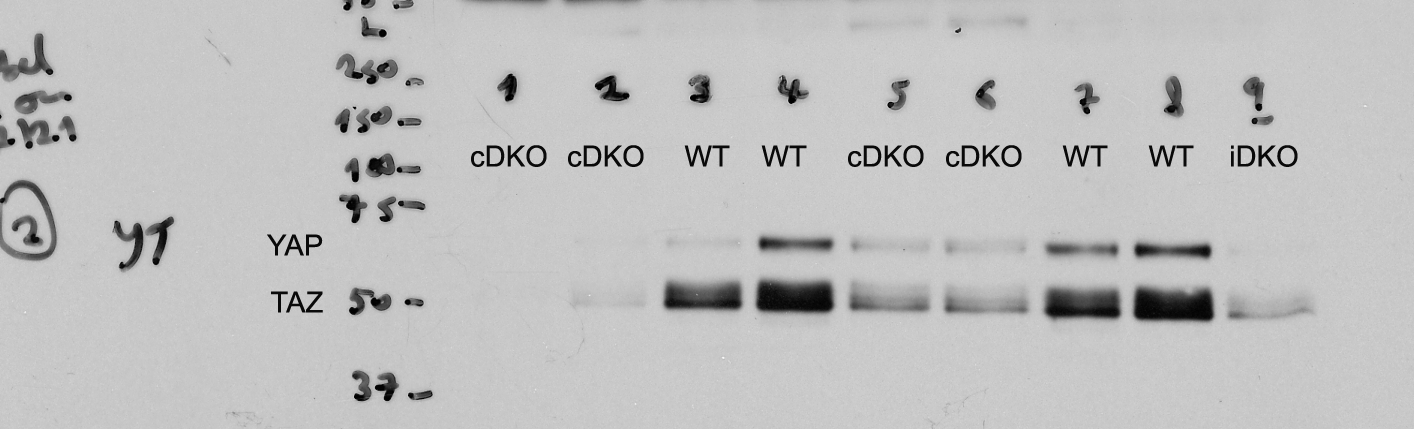

Supplement: Figure 5—source data 2. [file elife-87394-fig5-data2.zip › Fig 5 souce data 2/Fig 5B P60 blots and prism files/YT cDKO Yap and Taz/uncropped 2 labeled.tif]

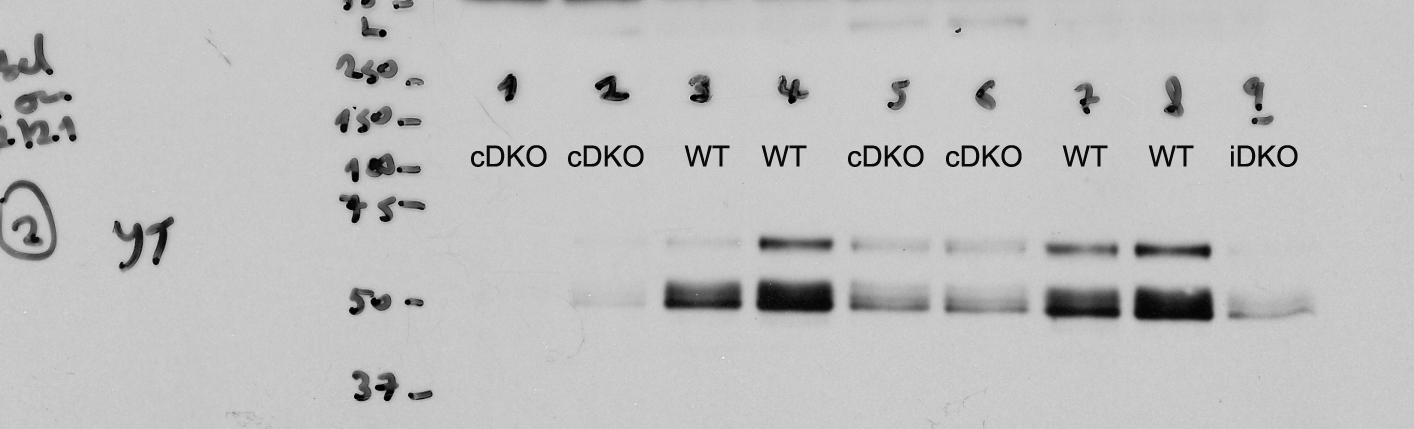

Supplement: Figure 5—source data 2. [file elife-87394-fig5-data2.zip › Fig 5 souce data 2/Fig 5B P60 blots and prism files/YT cDKO Yap and Taz/uncropped 2.tif]

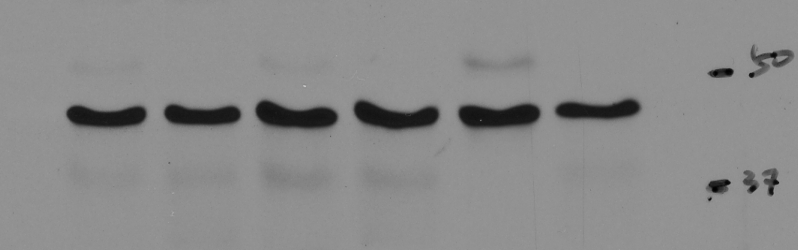

Supplement: Figure 6—source data 2. [file elife-87394-fig6-data2.zip › Fig 6 source data 2/Fig 6-S1 A blots/beta actin/uncropped beta actin.tif]

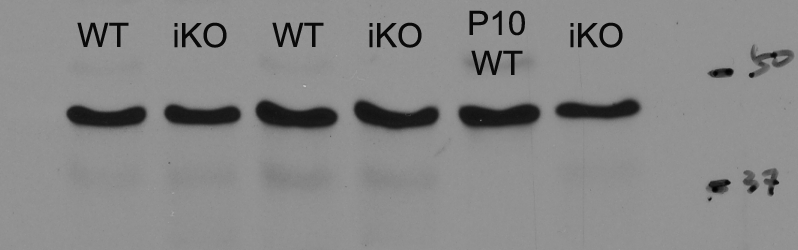

Supplement: Figure 6—source data 2. [file elife-87394-fig6-data2.zip › Fig 6 source data 2/Fig 6-S1 A blots/beta actin/uncropped labeled beta actin.tif]

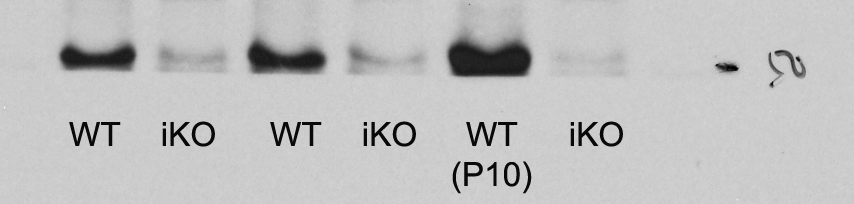

Supplement: Figure 6—source data 2. [file elife-87394-fig6-data2.zip › Fig 6 source data 2/Fig 6-S1 A blots/TEAD1/uncropped labeled TEAD1.tif]

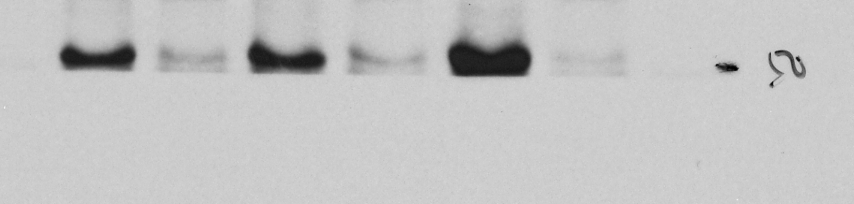

Supplement: Figure 6—source data 2. [file elife-87394-fig6-data2.zip › Fig 6 source data 2/Fig 6-S1 A blots/TEAD1/uncropped TEAD1.tif]

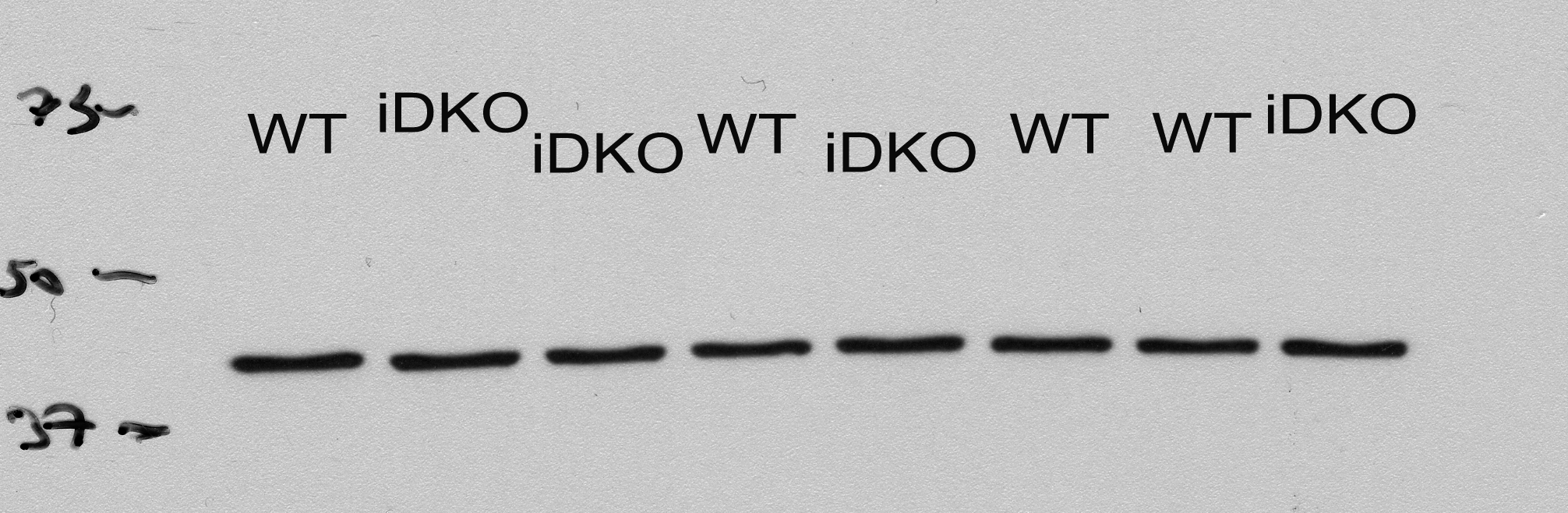

Supplement: Figure 7—source data 1. [file elife-87394-fig7-data1.zip › Fig 7 source data 1/Fig 7D blots and prism files/Yt iDKO actin for HMGCR/uncropped labeled.tif]

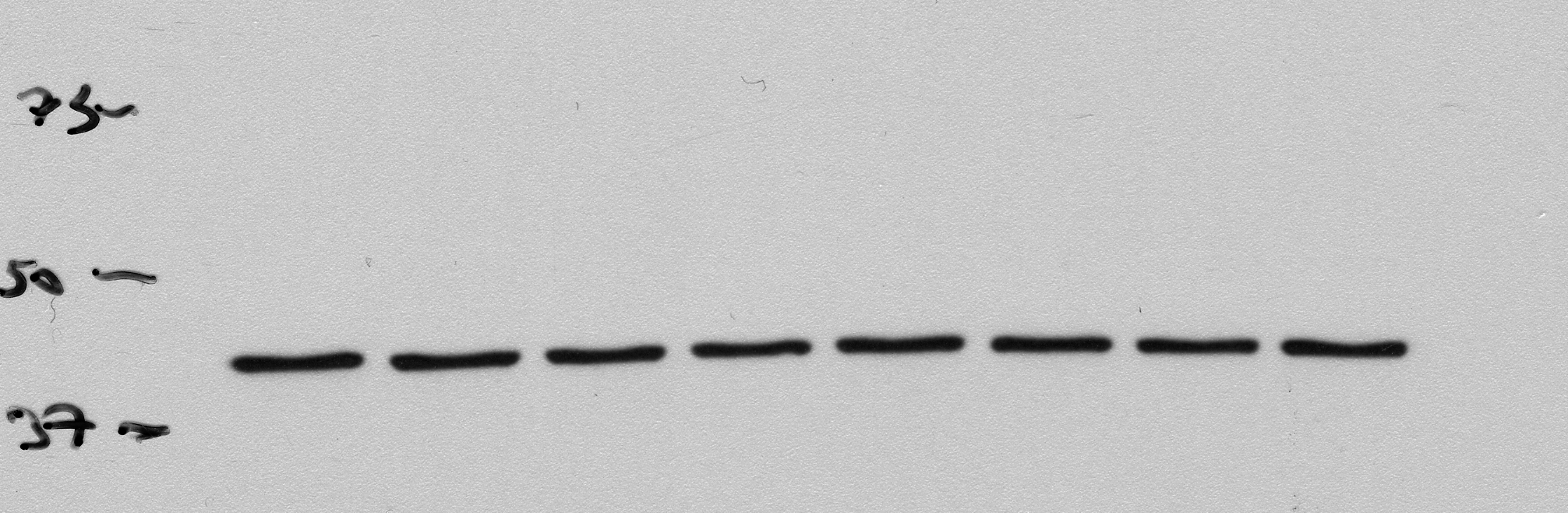

Supplement: Figure 7—source data 1. [file elife-87394-fig7-data1.zip › Fig 7 source data 1/Fig 7D blots and prism files/Yt iDKO actin for HMGCR/uncropped.tif]

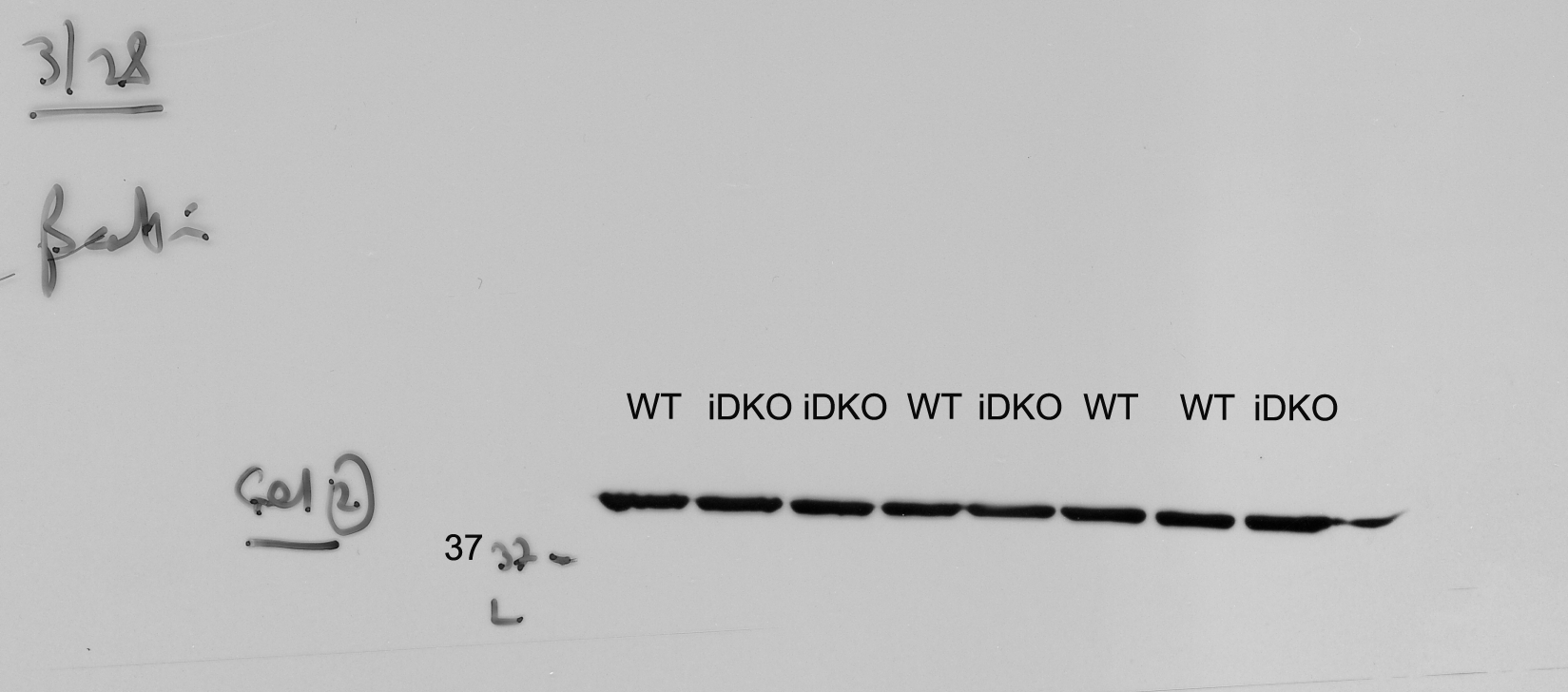

Supplement: Figure 7—source data 1. [file elife-87394-fig7-data1.zip › Fig 7 source data 1/Fig 7D blots and prism files/YT iDKO actin for SCD1/uncropped labeled.tif]

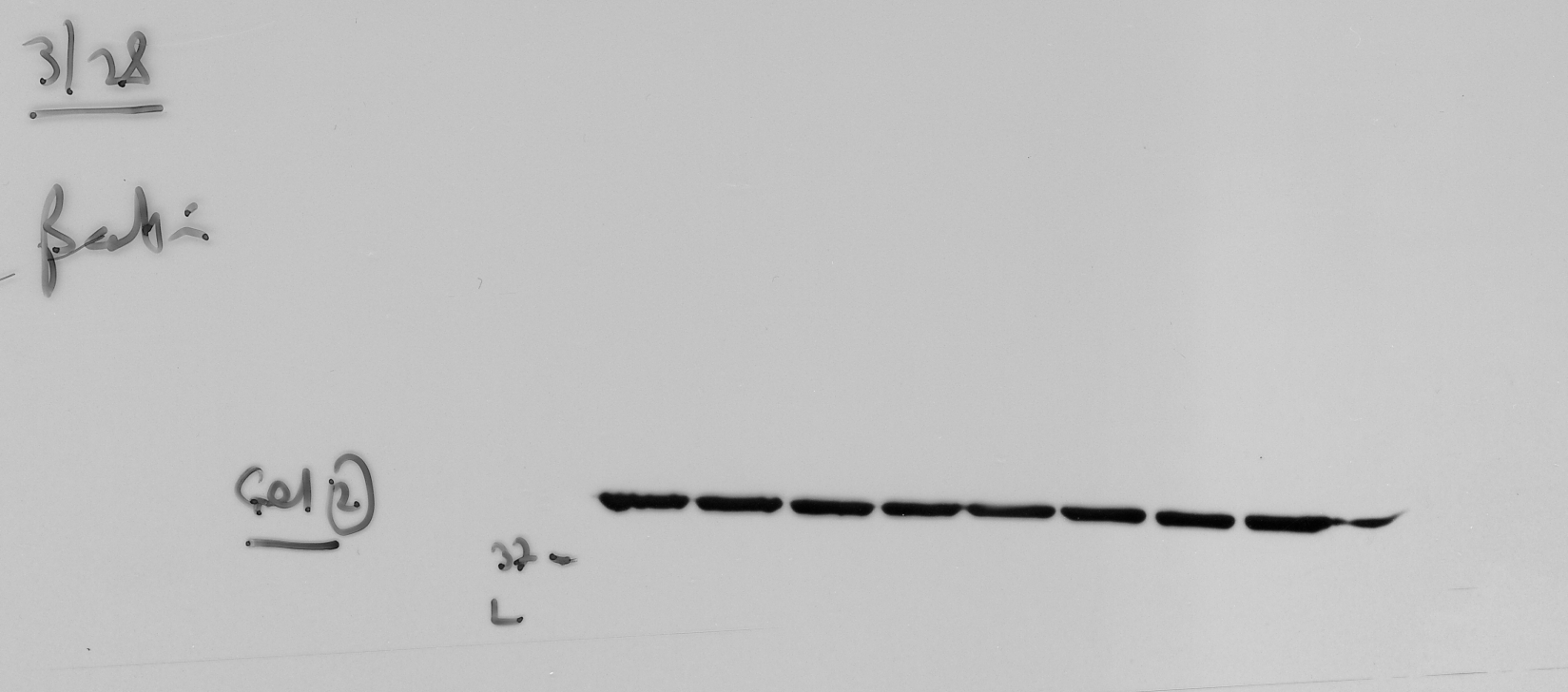

Supplement: Figure 7—source data 1. [file elife-87394-fig7-data1.zip › Fig 7 source data 1/Fig 7D blots and prism files/YT iDKO actin for SCD1/uncropped.tif]

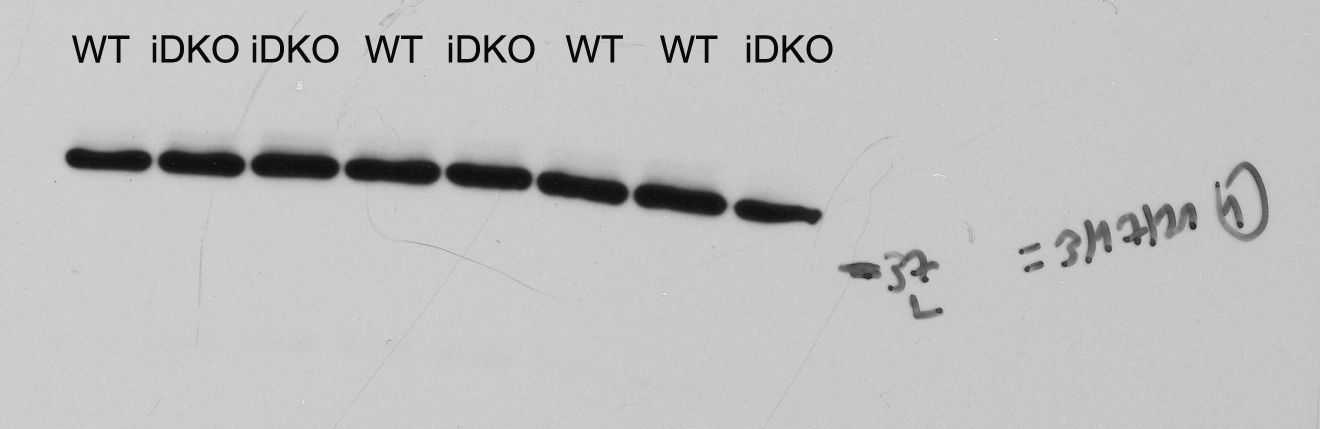

Supplement: Figure 7—source data 1. [file elife-87394-fig7-data1.zip › Fig 7 source data 1/Fig 7D blots and prism files/YT iDKO actin for SREBP1/uncropped labeled.tif]

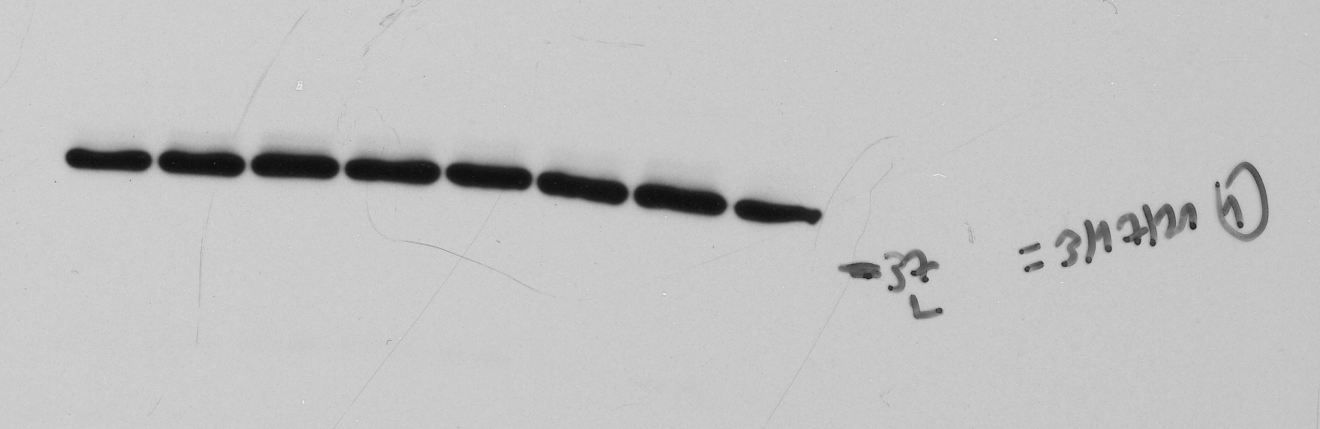

Supplement: Figure 7—source data 1. [file elife-87394-fig7-data1.zip › Fig 7 source data 1/Fig 7D blots and prism files/YT iDKO actin for SREBP1/uncropped.tif]

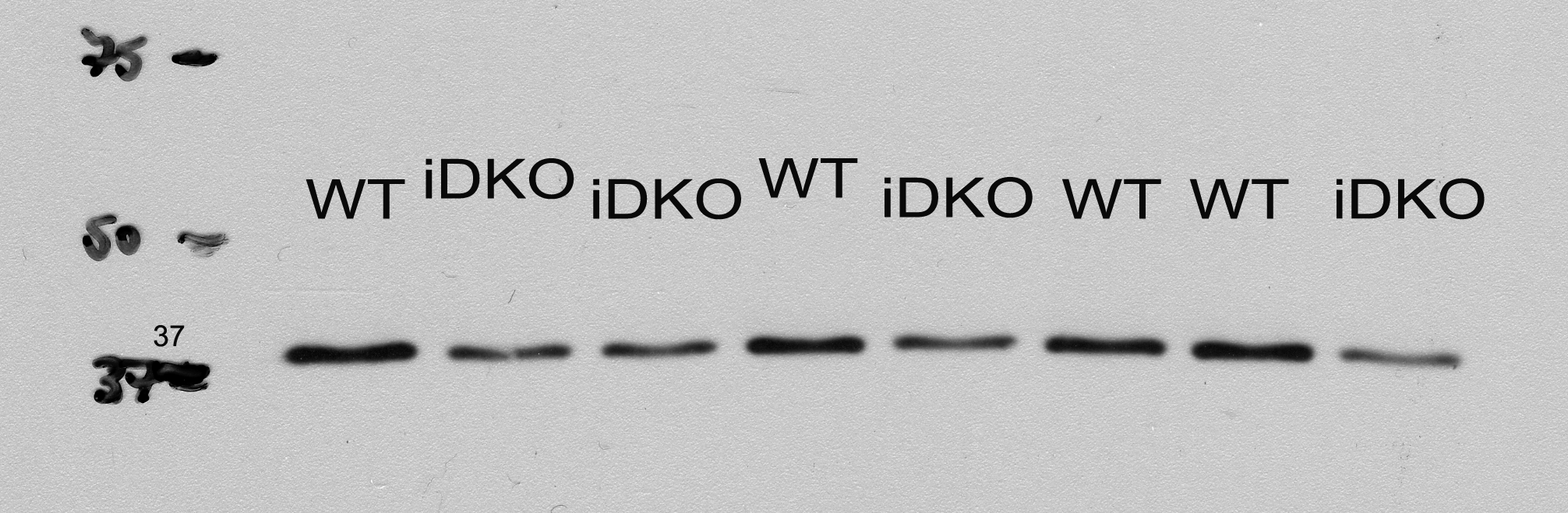

Supplement: Figure 7—source data 1. [file elife-87394-fig7-data1.zip › Fig 7 source data 1/Fig 7D blots and prism files/YT iDKO FDPS/uncropped labeled.tif]

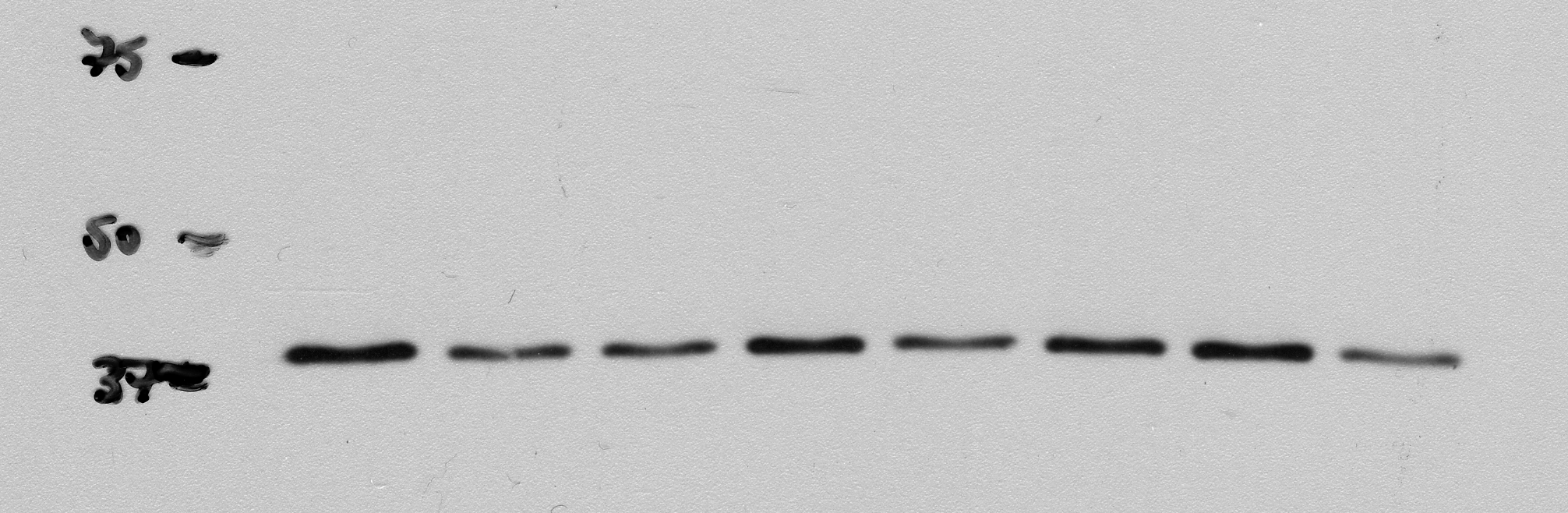

Supplement: Figure 7—source data 1. [file elife-87394-fig7-data1.zip › Fig 7 source data 1/Fig 7D blots and prism files/YT iDKO FDPS/uncropped.tif]

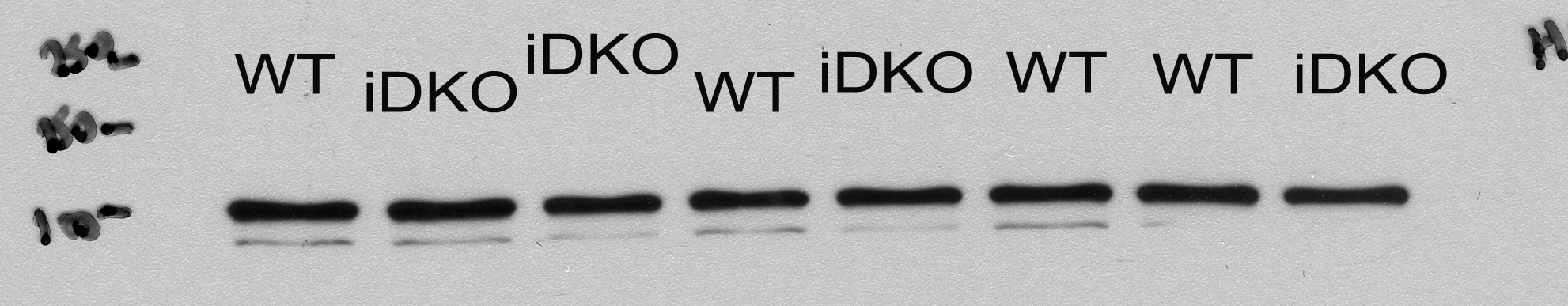

Supplement: Figure 7—source data 1. [file elife-87394-fig7-data1.zip › Fig 7 source data 1/Fig 7D blots and prism files/YT iDKO HMGCR/uncropped labeled.tif]

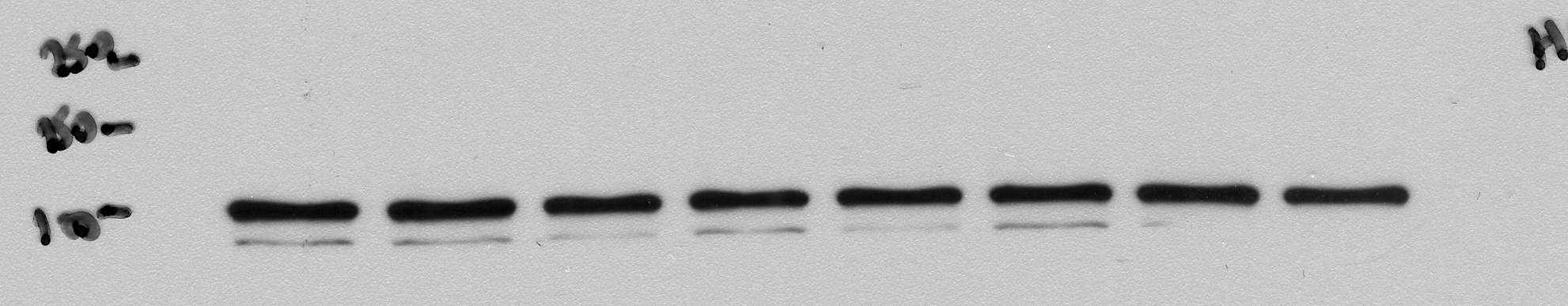

Supplement: Figure 7—source data 1. [file elife-87394-fig7-data1.zip › Fig 7 source data 1/Fig 7D blots and prism files/YT iDKO HMGCR/uncropped.tif]

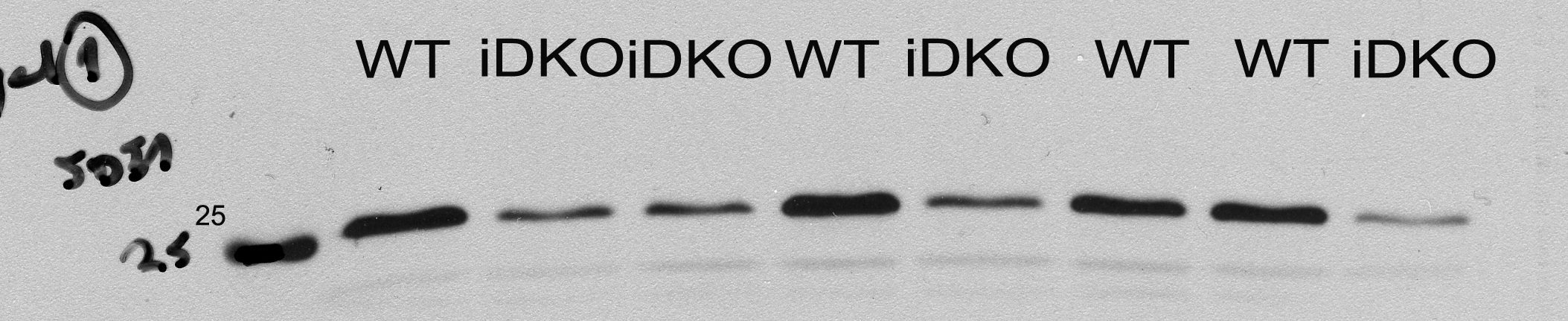

Supplement: Figure 7—source data 1. [file elife-87394-fig7-data1.zip › Fig 7 source data 1/Fig 7D blots and prism files/YT iDKO IDI1/uncropped labeled.tif]

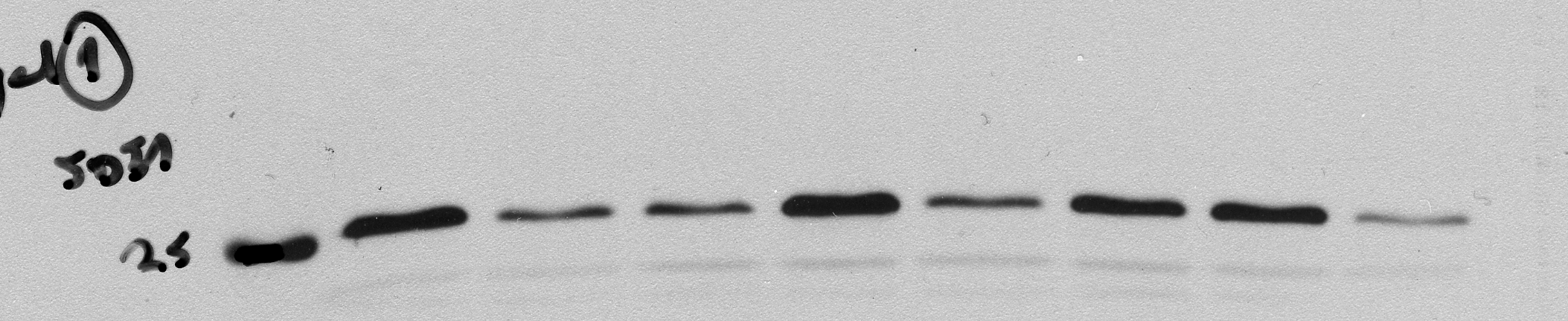

Supplement: Figure 7—source data 1. [file elife-87394-fig7-data1.zip › Fig 7 source data 1/Fig 7D blots and prism files/YT iDKO IDI1/uncropped.tif]

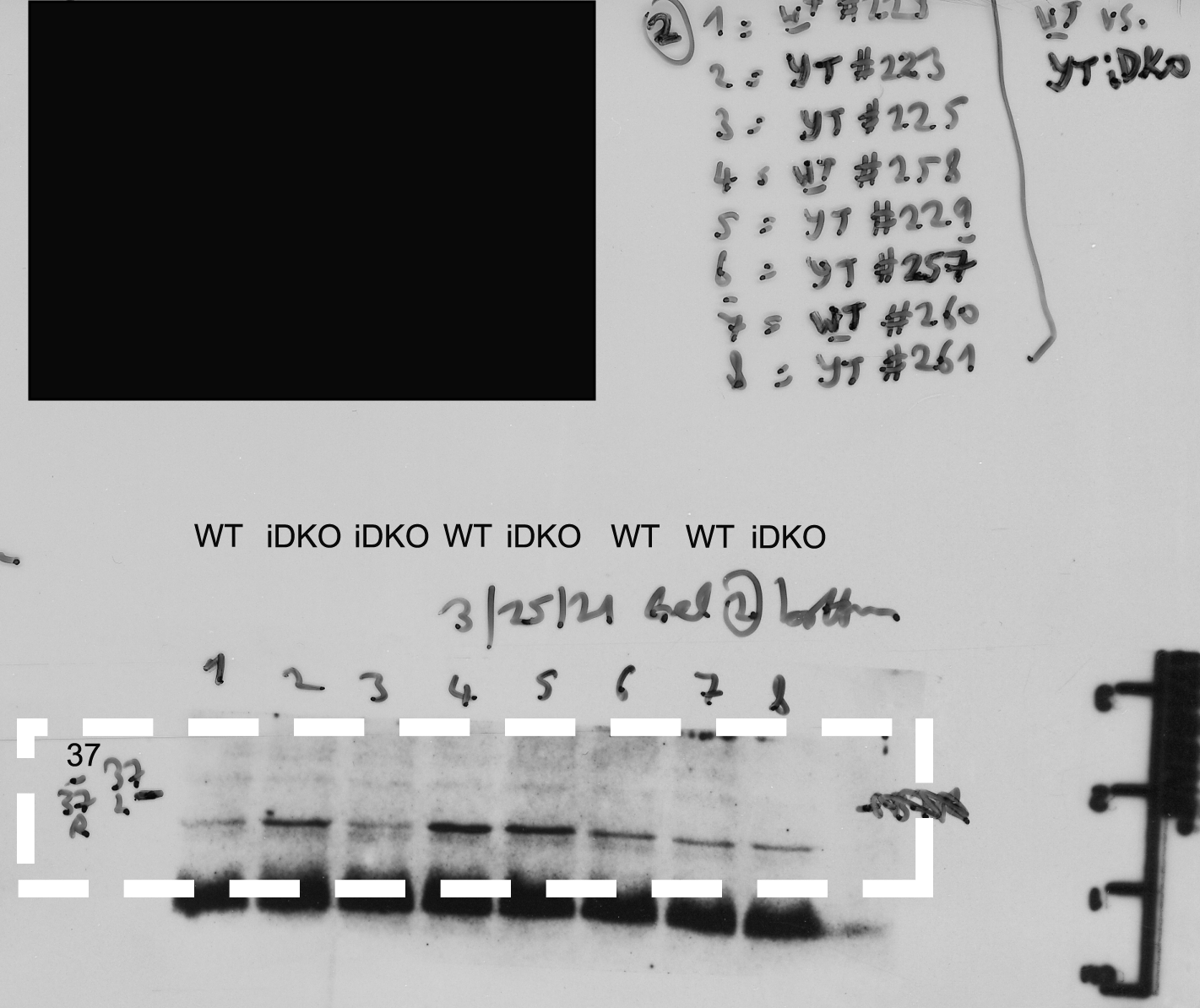

Supplement: Figure 7—source data 1. [file elife-87394-fig7-data1.zip › Fig 7 source data 1/Fig 7D blots and prism files/YT iDKO SCD1/uncropped labeled.tif]

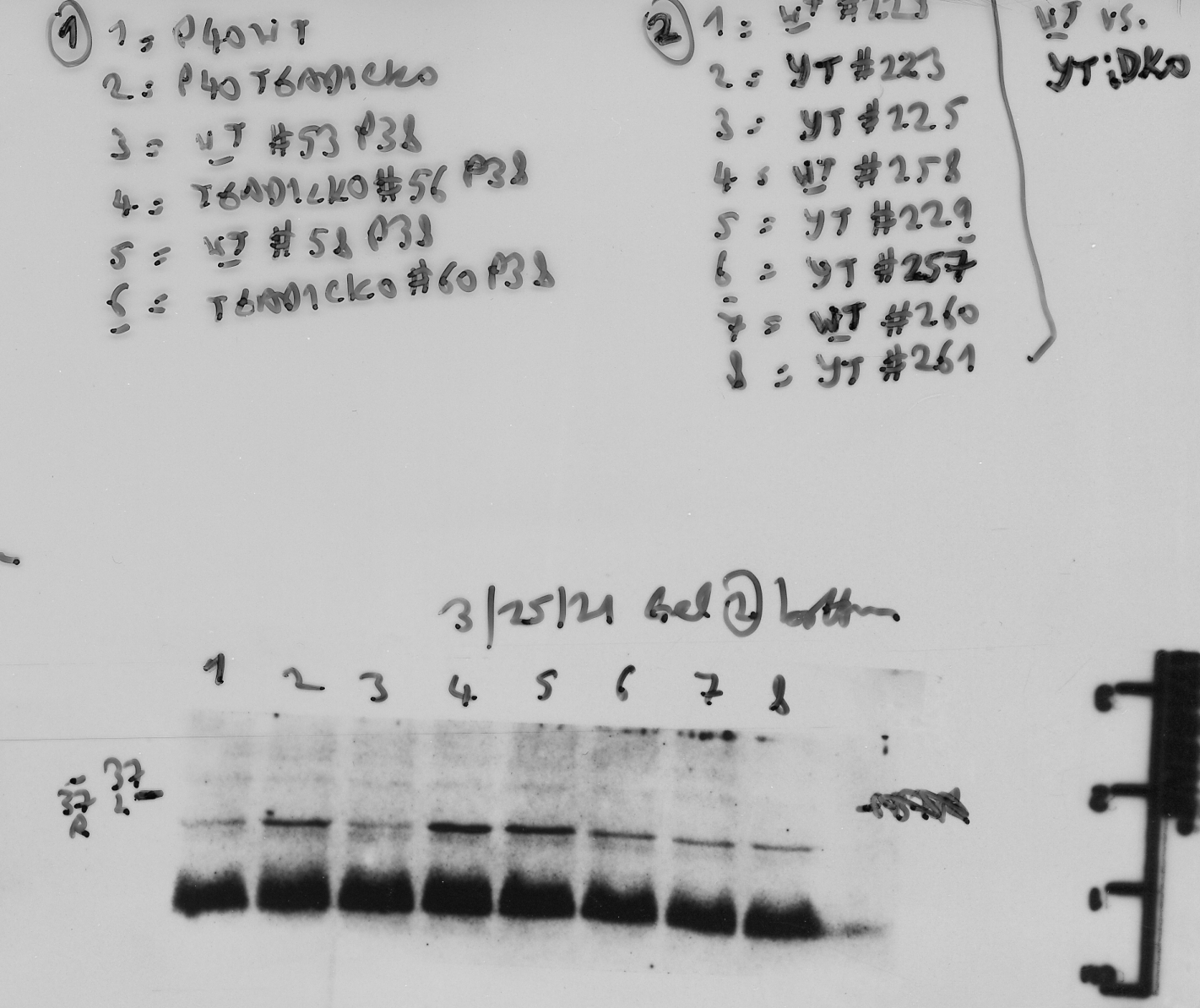

Supplement: Figure 7—source data 1. [file elife-87394-fig7-data1.zip › Fig 7 source data 1/Fig 7D blots and prism files/YT iDKO SCD1/uncropped.tif]

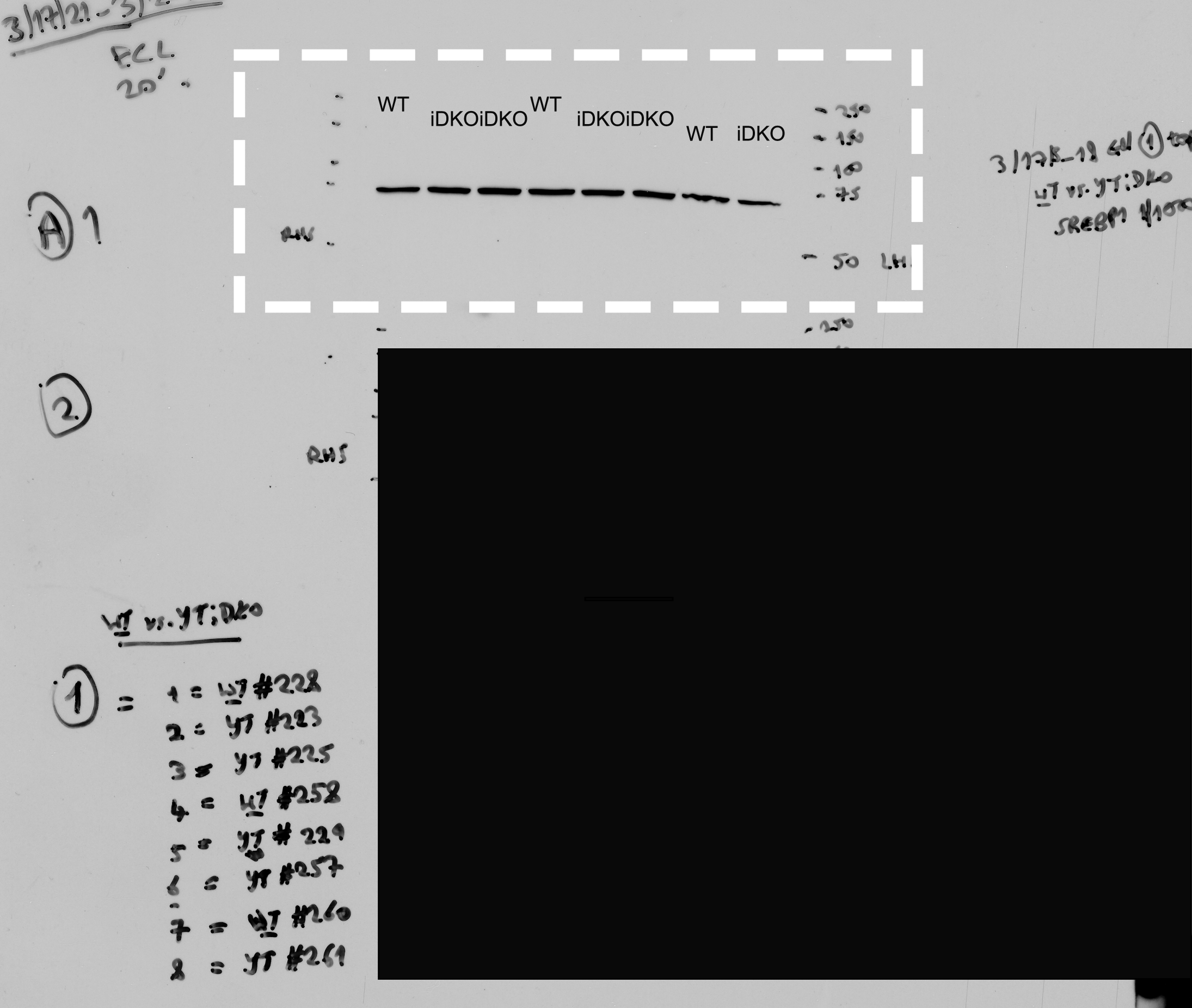

Supplement: Figure 7—source data 1. [file elife-87394-fig7-data1.zip › Fig 7 source data 1/Fig 7D blots and prism files/YT iDKO SREBP1/uncropped labeled.tif]

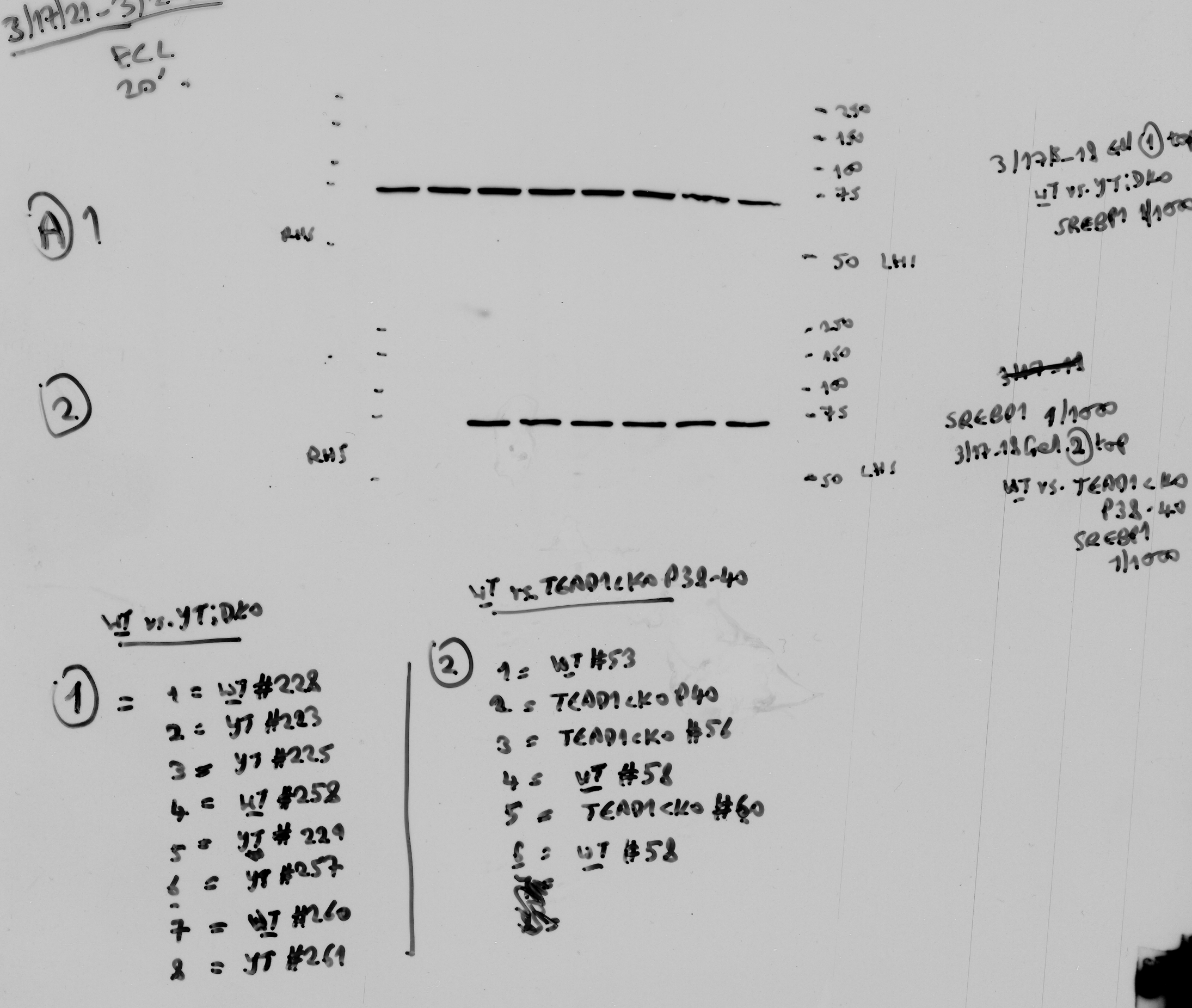

Supplement: Figure 7—source data 1. [file elife-87394-fig7-data1.zip › Fig 7 source data 1/Fig 7D blots and prism files/YT iDKO SREBP1/uncropped.tif]

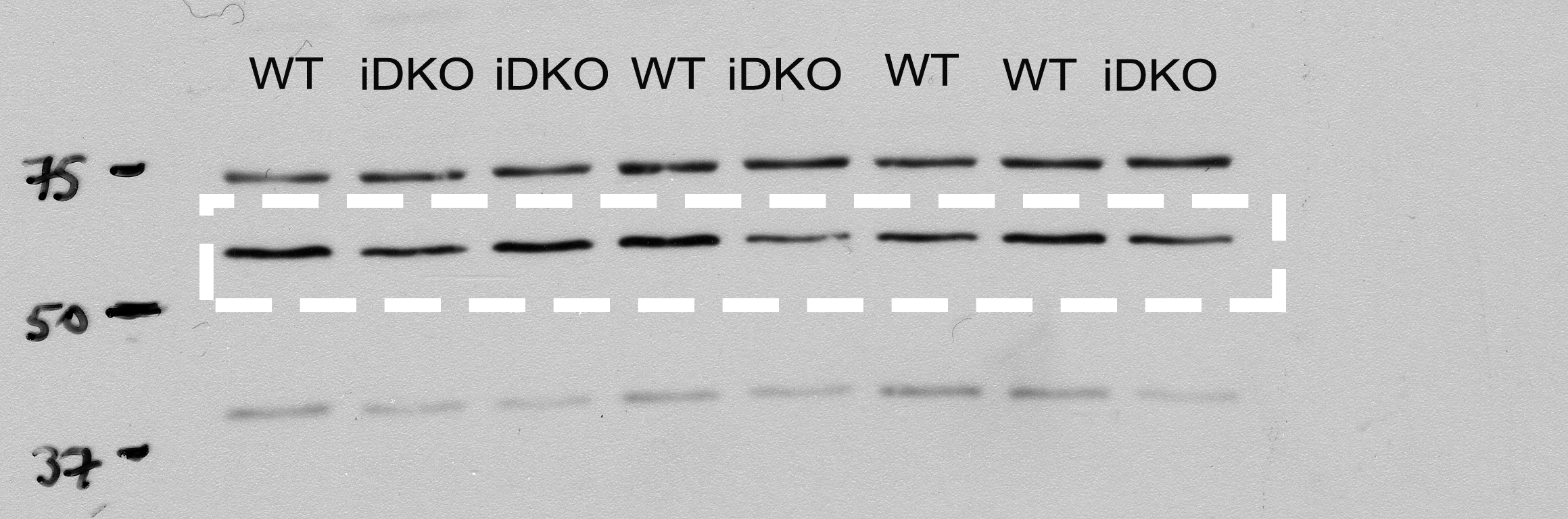

Supplement: Figure 7—source data 1. [file elife-87394-fig7-data1.zip › Fig 7 source data 1/Fig 7D blots and prism files/YT iDKO SREBP2/uncropped labeled.tif]

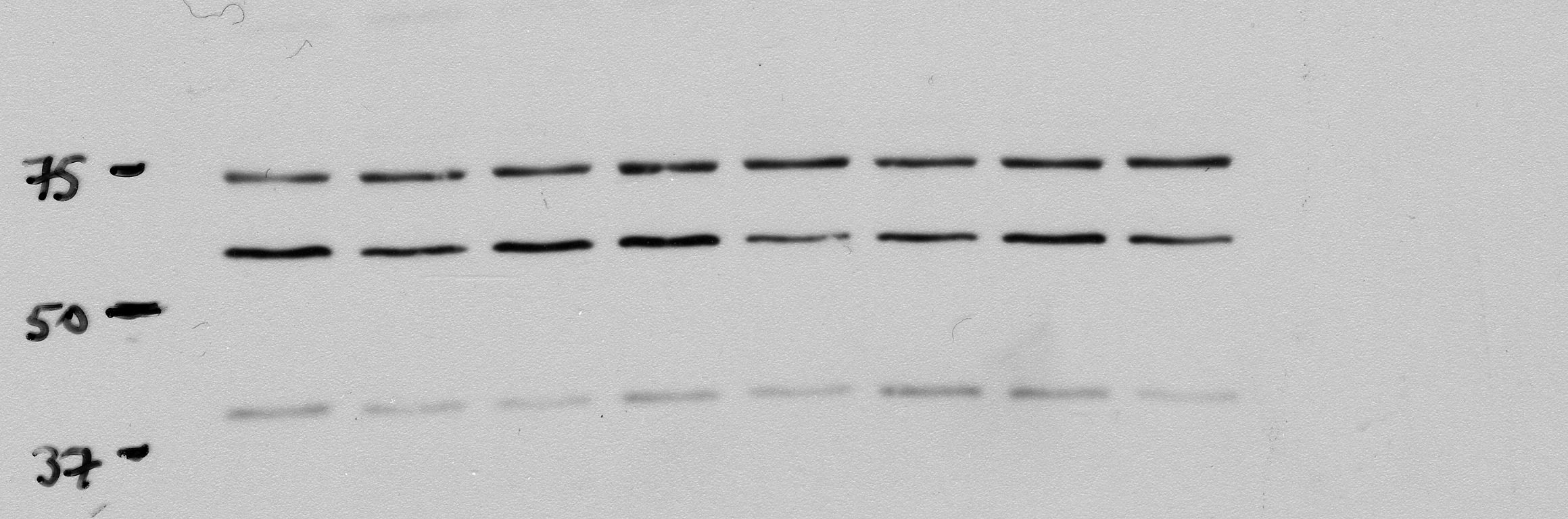

Supplement: Figure 7—source data 1. [file elife-87394-fig7-data1.zip › Fig 7 source data 1/Fig 7D blots and prism files/YT iDKO SREBP2/uncropped.tif]

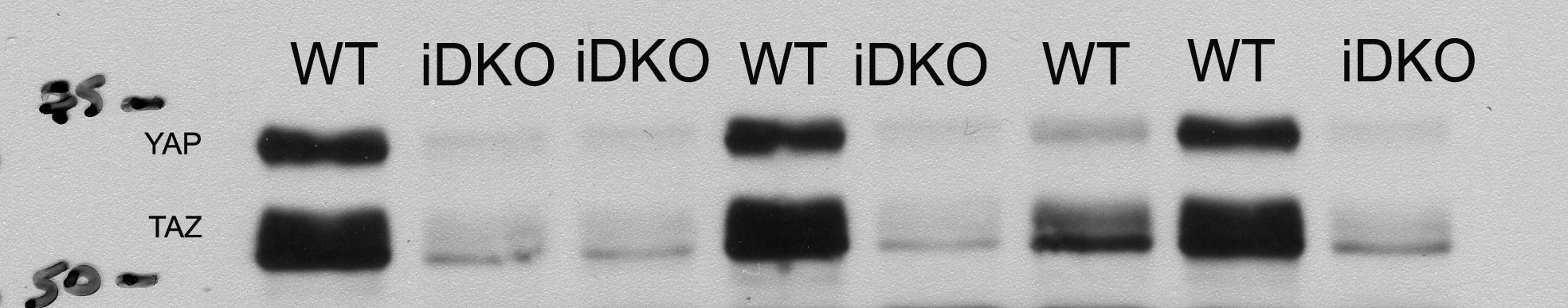

Supplement: Figure 7—source data 1. [file elife-87394-fig7-data1.zip › Fig 7 source data 1/Fig 7D blots and prism files/YT iDKO Yap Taz/uncropped 1 labeled.tif]

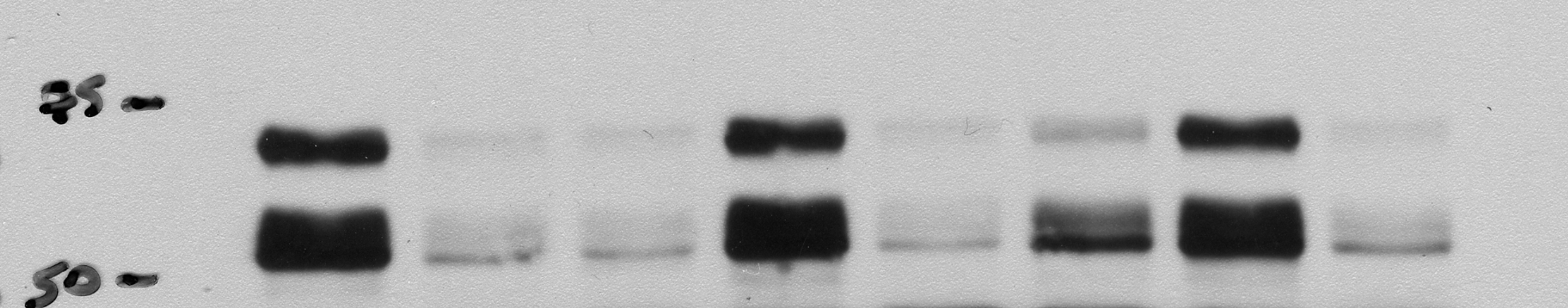

Supplement: Figure 7—source data 1. [file elife-87394-fig7-data1.zip › Fig 7 source data 1/Fig 7D blots and prism files/YT iDKO Yap Taz/uncropped 1.tif]

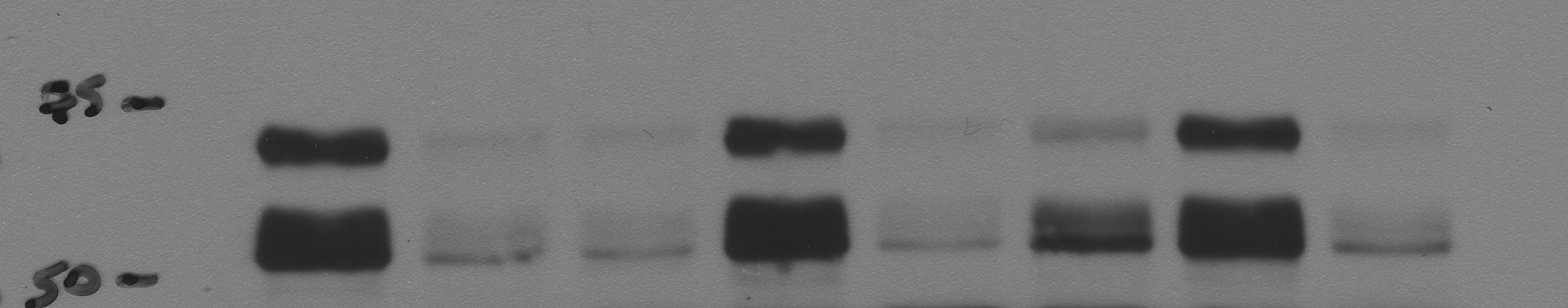

Supplement: Figure 7—source data 1. [file elife-87394-fig7-data1.zip › Fig 7 source data 1/Fig 7D blots and prism files/YT iDKO Yap Taz/uncropped 2.tif]

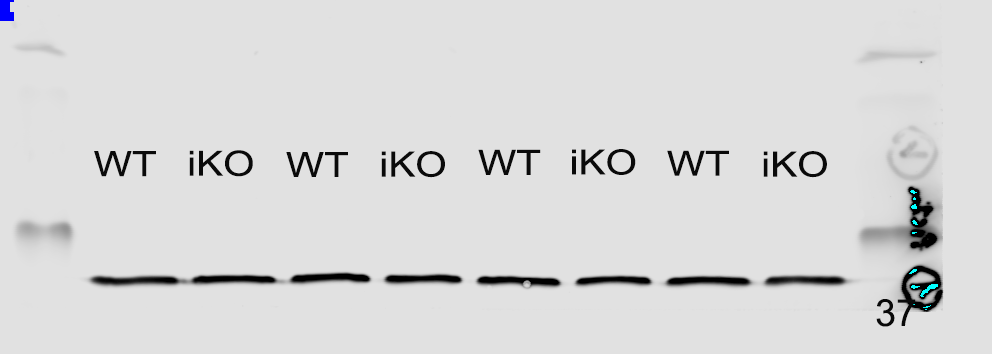

Supplement: Figure 7—source data 1. [file elife-87394-fig7-data1.zip › Fig 7 source data 1/Fig 7E blots and prism files/Tead1 iKO actin for HMGCR/uncropped labeled.tif]

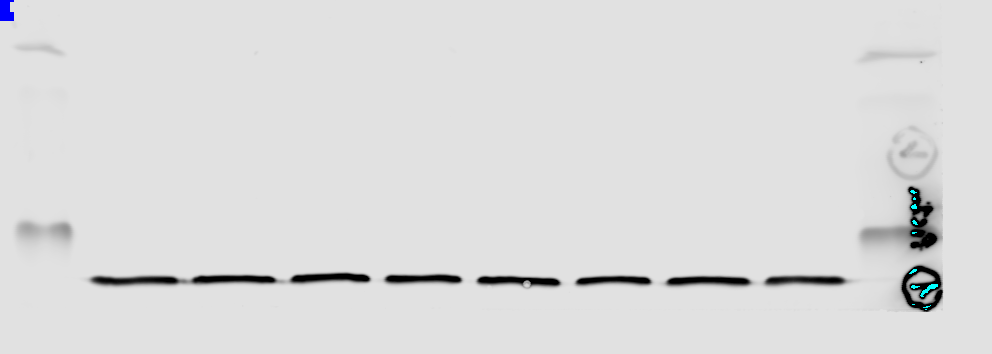

Supplement: Figure 7—source data 1. [file elife-87394-fig7-data1.zip › Fig 7 source data 1/Fig 7E blots and prism files/Tead1 iKO actin for HMGCR/uncropped.tif]

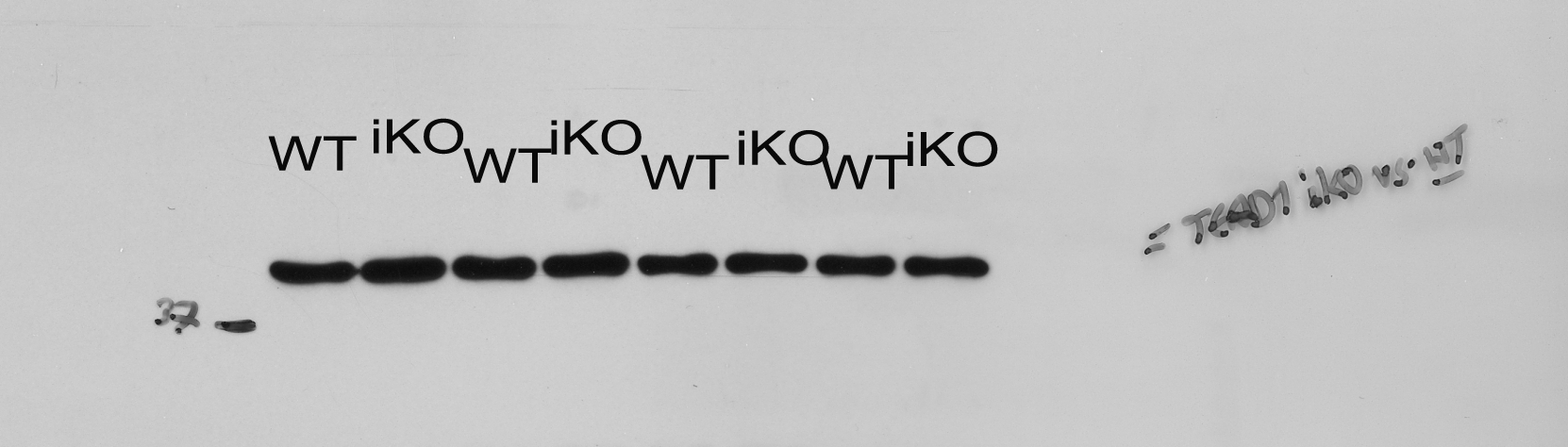

Supplement: Figure 7—source data 1. [file elife-87394-fig7-data1.zip › Fig 7 source data 1/Fig 7E blots and prism files/Tead1 iKO actin for SREBP1/uncropped labeled.tif]

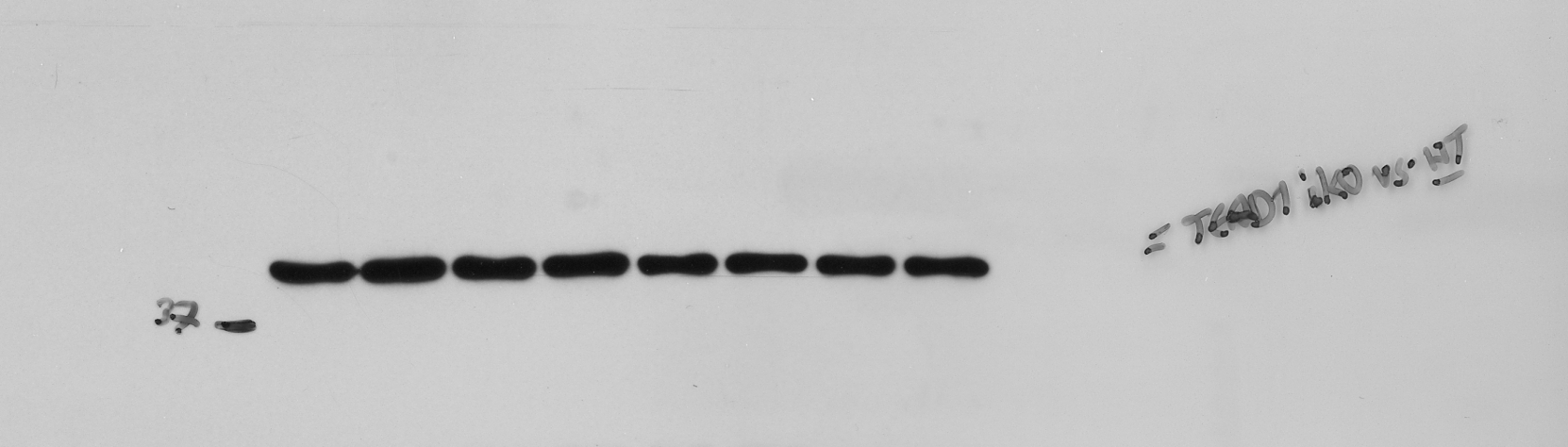

Supplement: Figure 7—source data 1. [file elife-87394-fig7-data1.zip › Fig 7 source data 1/Fig 7E blots and prism files/Tead1 iKO actin for SREBP1/uncropped.tif]

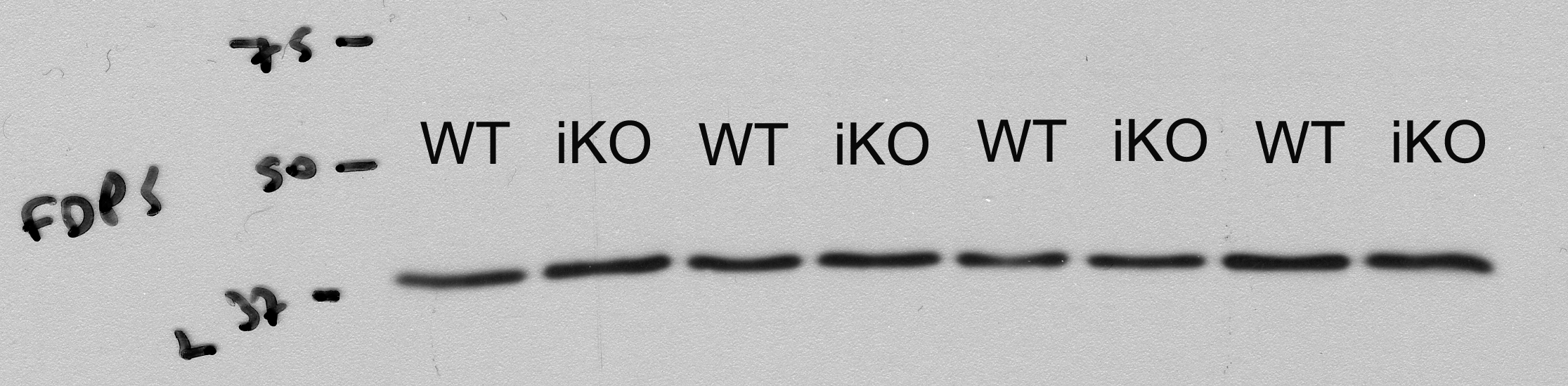

Supplement: Figure 7—source data 1. [file elife-87394-fig7-data1.zip › Fig 7 source data 1/Fig 7E blots and prism files/Tead1 iKO FDPS/uncropped labeled.tif]

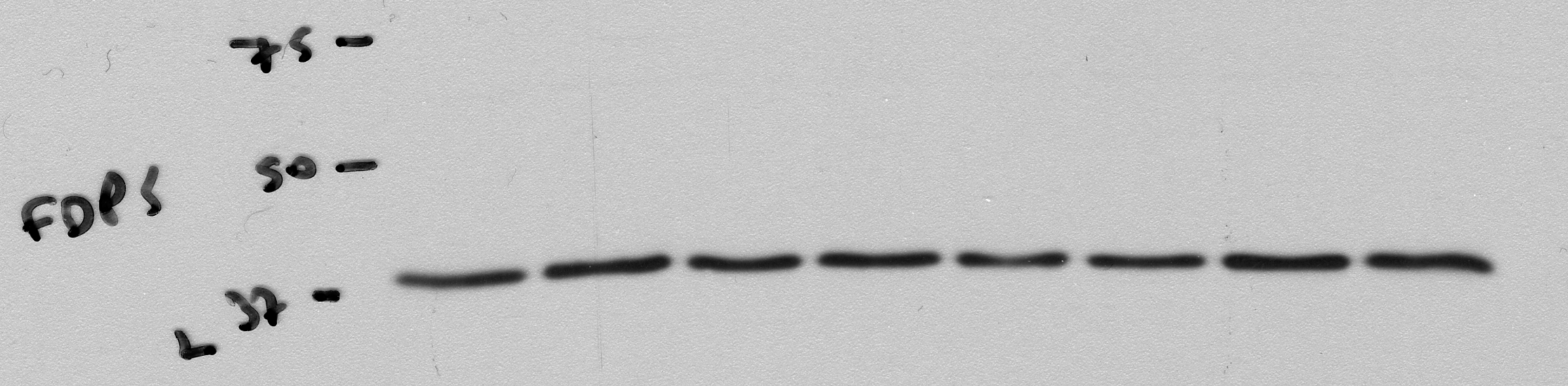

Supplement: Figure 7—source data 1. [file elife-87394-fig7-data1.zip › Fig 7 source data 1/Fig 7E blots and prism files/Tead1 iKO FDPS/uncropped.tif]

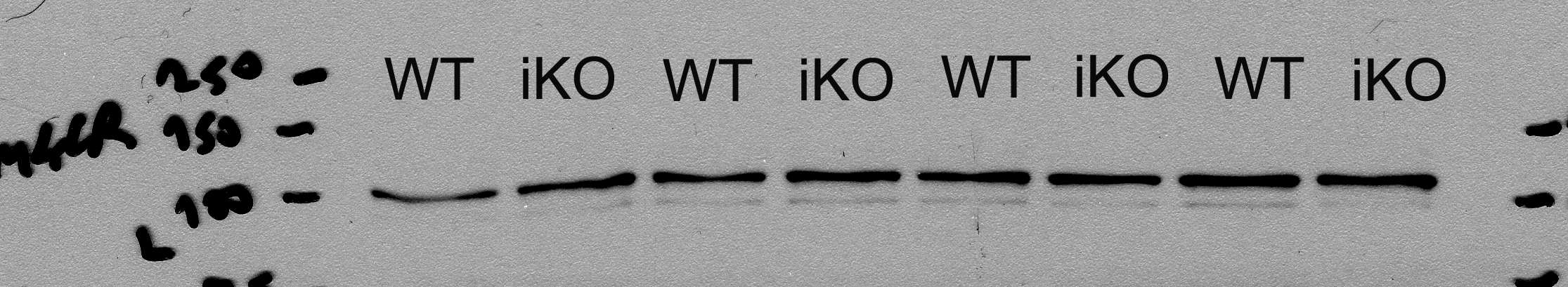

Supplement: Figure 7—source data 1. [file elife-87394-fig7-data1.zip › Fig 7 source data 1/Fig 7E blots and prism files/Tead1 iKO HMGCR/uncropped labeled.tif]

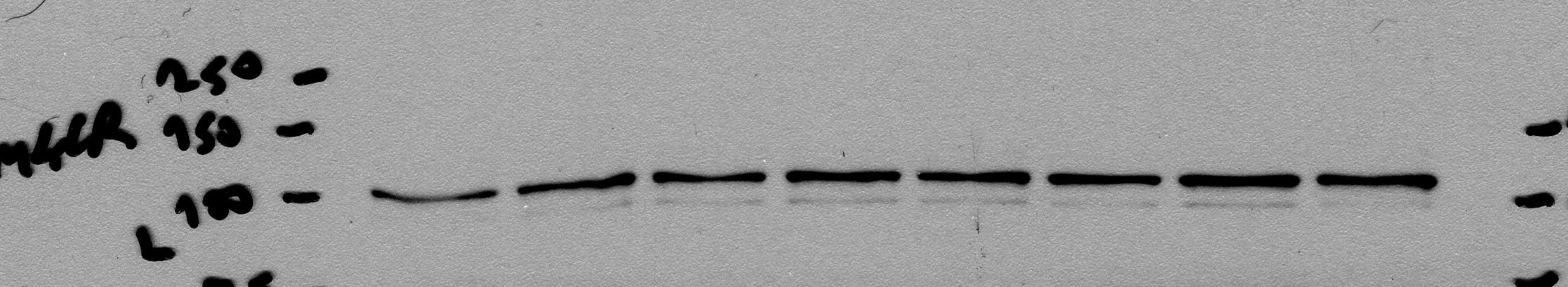

Supplement: Figure 7—source data 1. [file elife-87394-fig7-data1.zip › Fig 7 source data 1/Fig 7E blots and prism files/Tead1 iKO HMGCR/uncropped.tif]

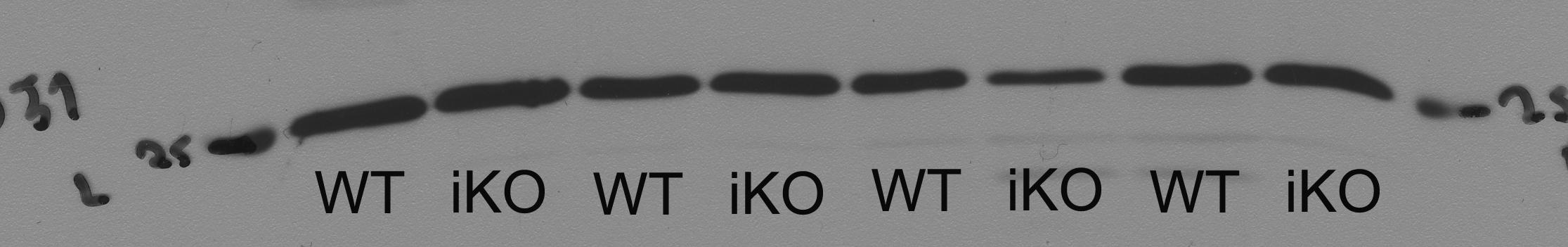

Supplement: Figure 7—source data 1. [file elife-87394-fig7-data1.zip › Fig 7 source data 1/Fig 7E blots and prism files/Tead1 iKO IDI1/uncropped labeled.tif]

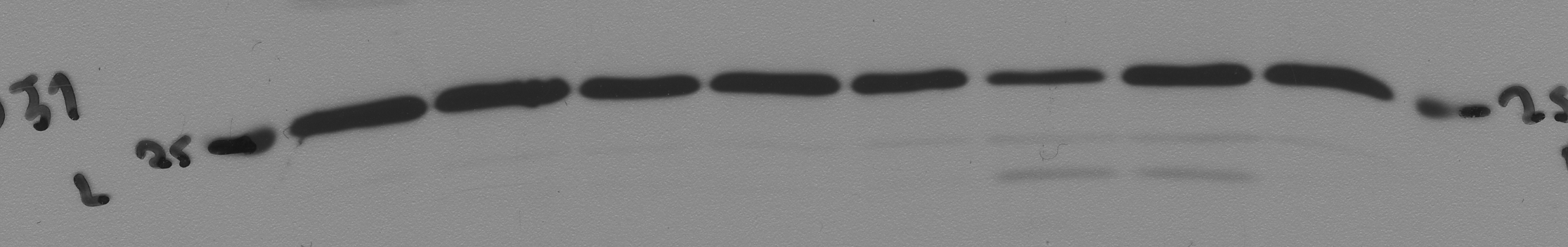

Supplement: Figure 7—source data 1. [file elife-87394-fig7-data1.zip › Fig 7 source data 1/Fig 7E blots and prism files/Tead1 iKO IDI1/uncropped.tif]

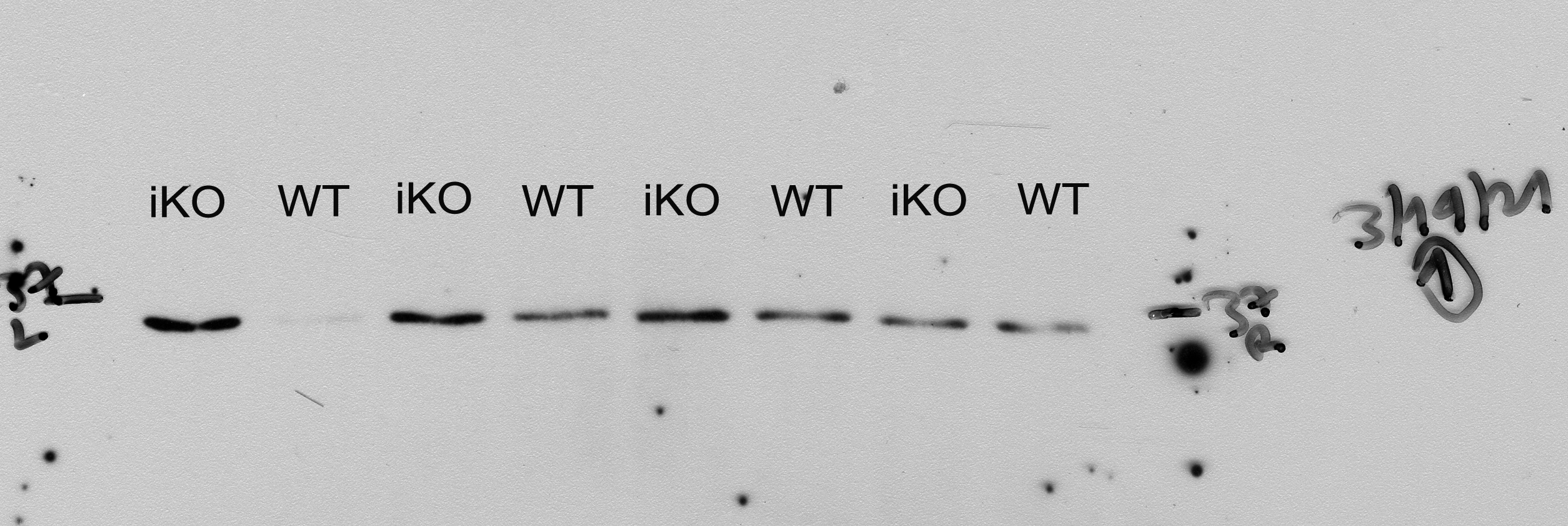

Supplement: Figure 7—source data 1. [file elife-87394-fig7-data1.zip › Fig 7 source data 1/Fig 7E blots and prism files/Tead1 iKO SCD1/uncropped labeled.tif]

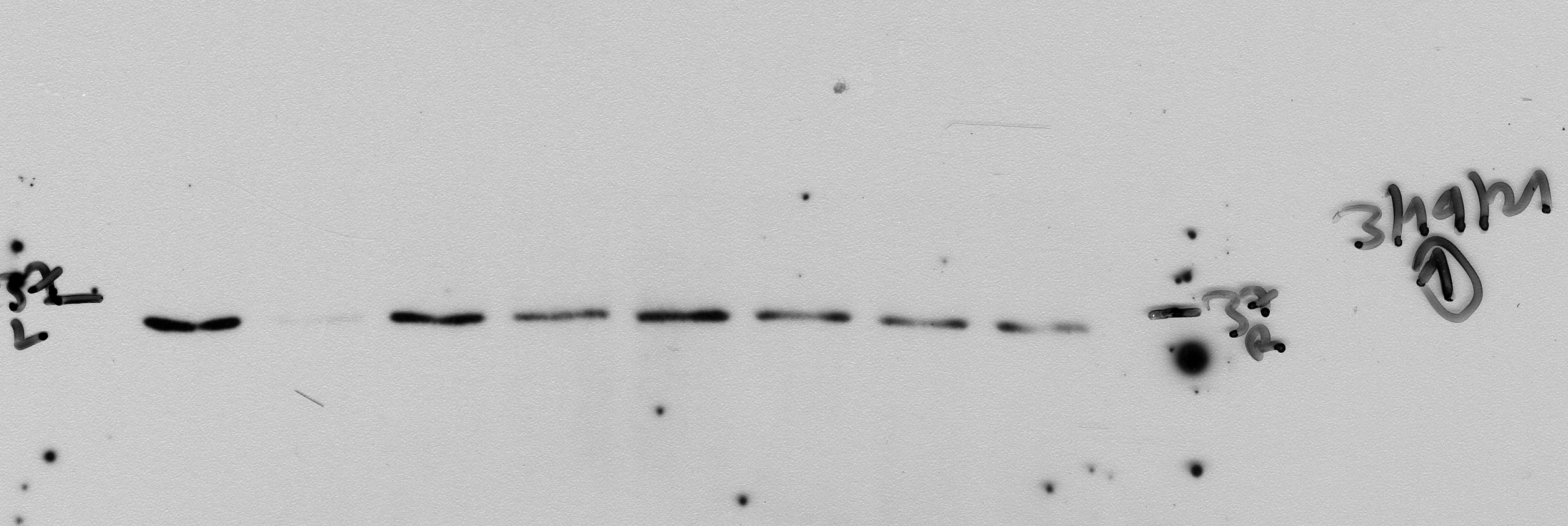

Supplement: Figure 7—source data 1. [file elife-87394-fig7-data1.zip › Fig 7 source data 1/Fig 7E blots and prism files/Tead1 iKO SCD1/uncropped.tif]

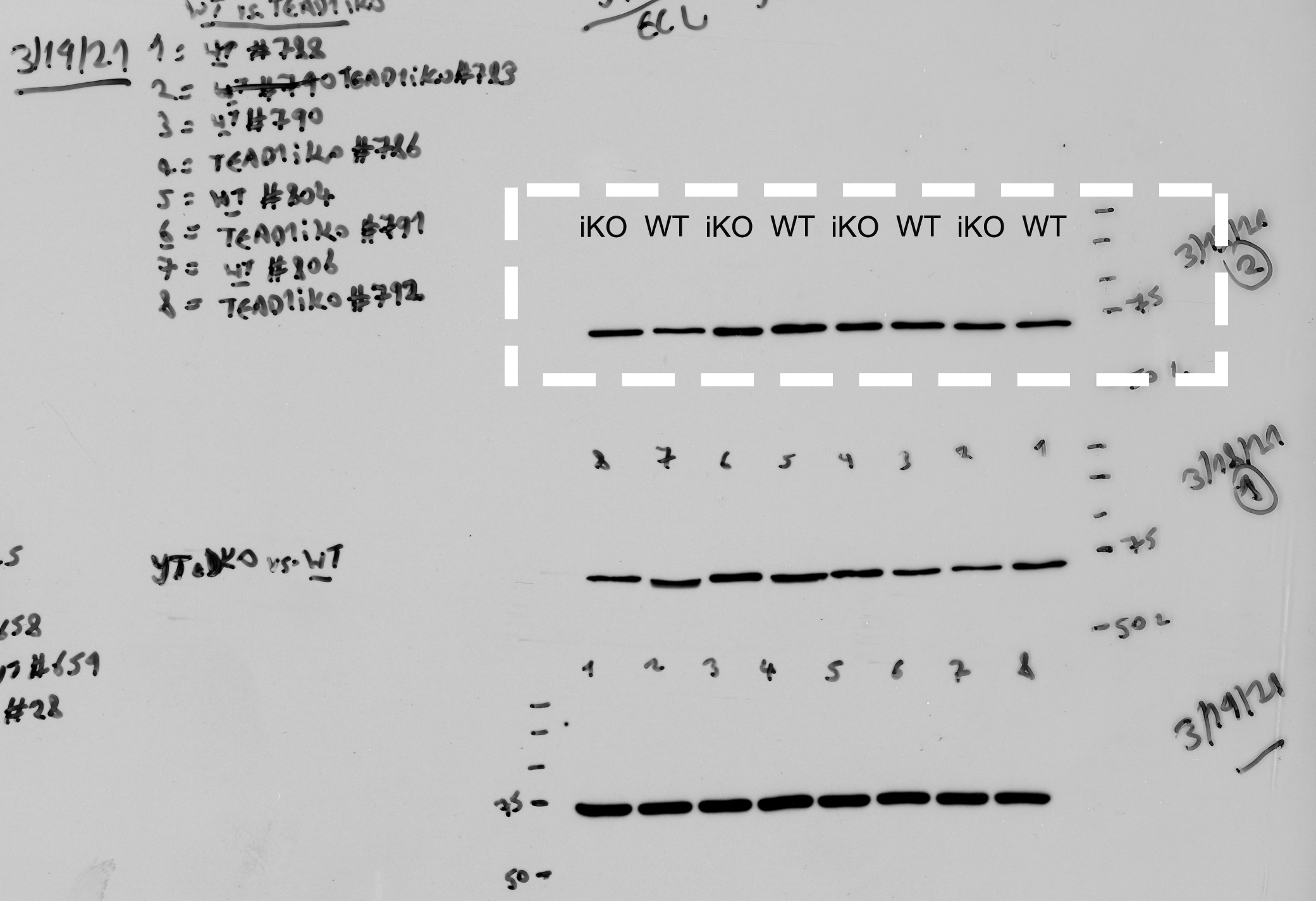

Supplement: Figure 7—source data 1. [file elife-87394-fig7-data1.zip › Fig 7 source data 1/Fig 7E blots and prism files/Tead1 iKO SREBP1/uncropped labeled.tif]

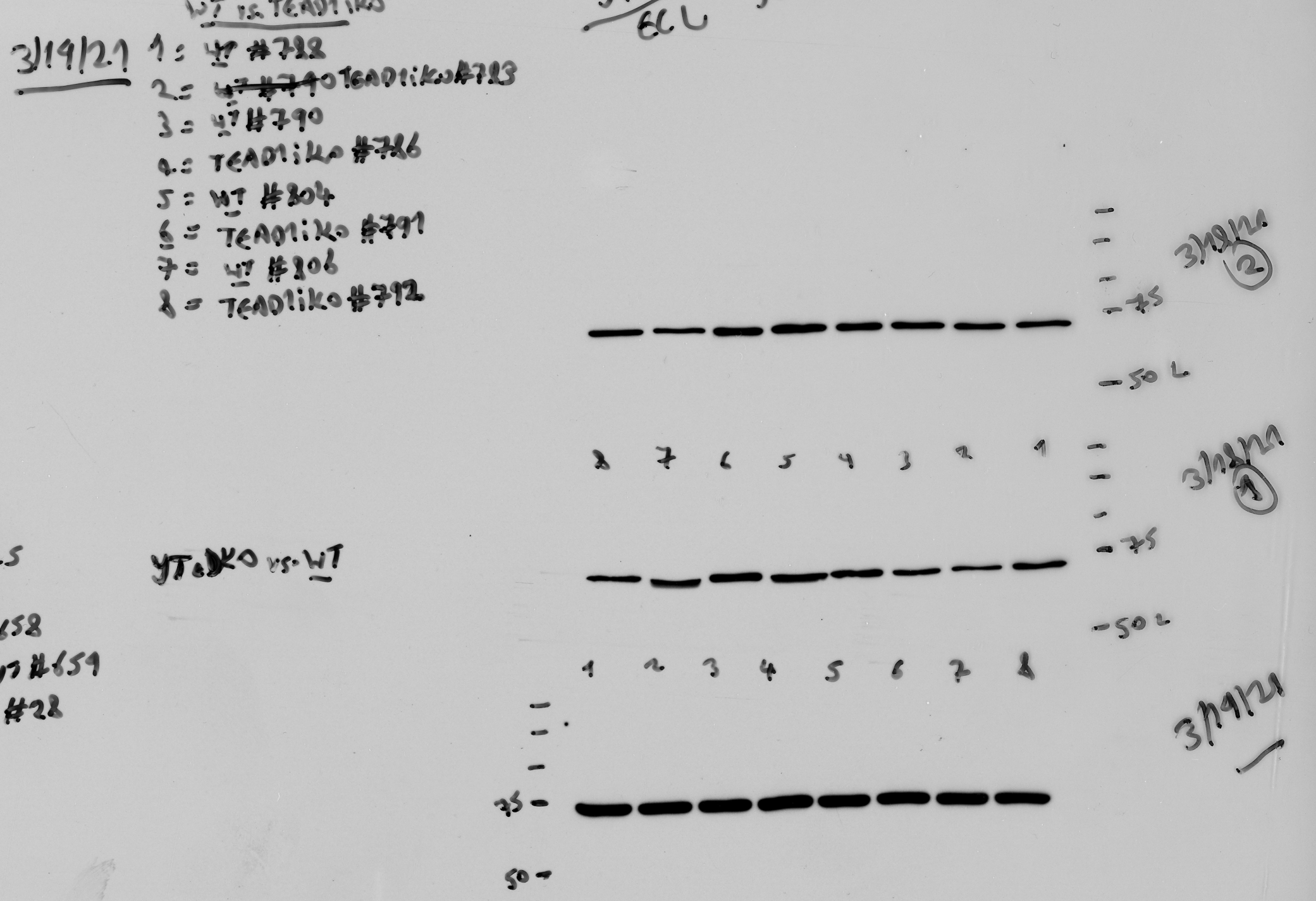

Supplement: Figure 7—source data 1. [file elife-87394-fig7-data1.zip › Fig 7 source data 1/Fig 7E blots and prism files/Tead1 iKO SREBP1/uncropped.tif]

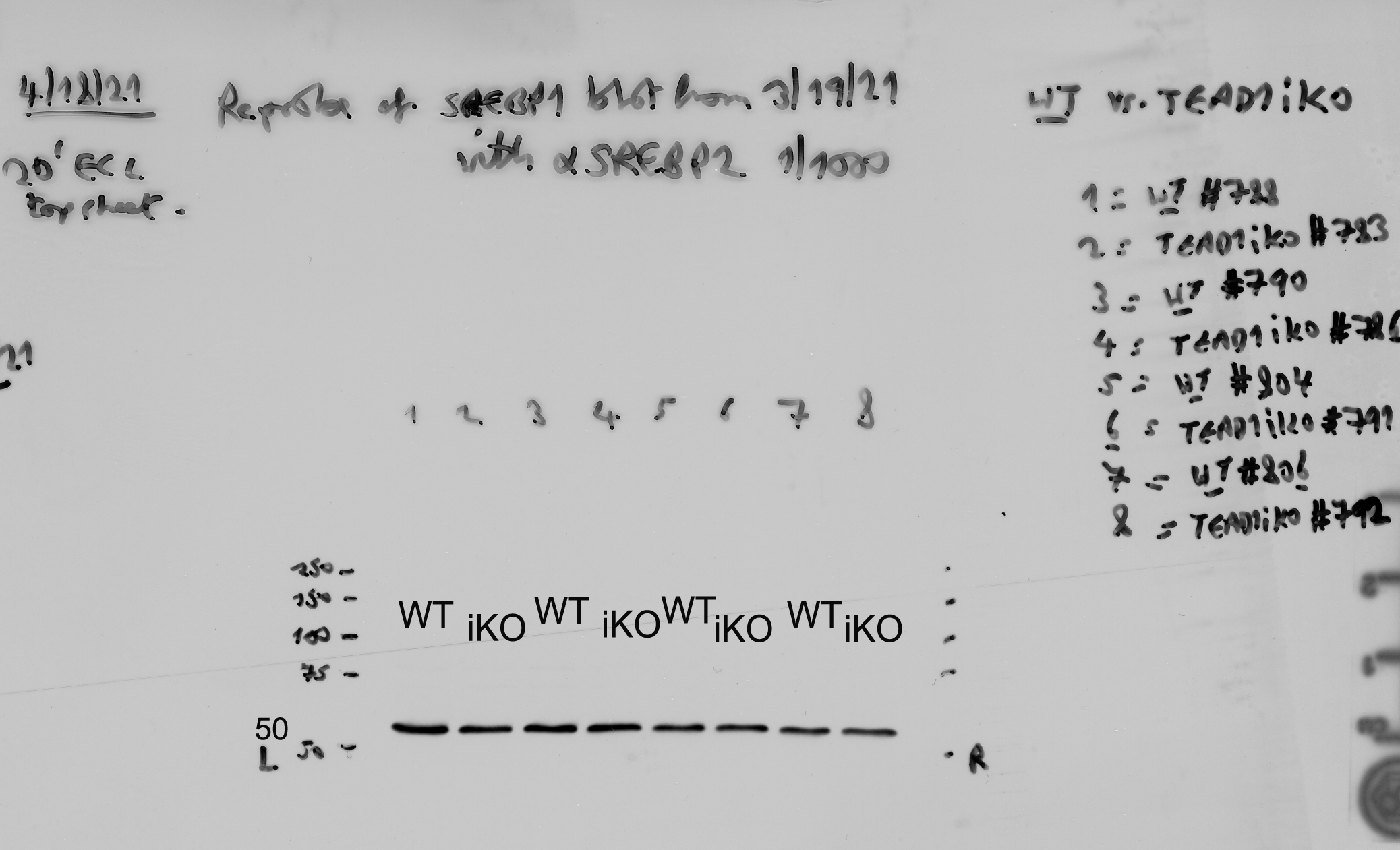

Supplement: Figure 7—source data 1. [file elife-87394-fig7-data1.zip › Fig 7 source data 1/Fig 7E blots and prism files/Tead1 iKO SREBP2/uncropped labeled.tif]

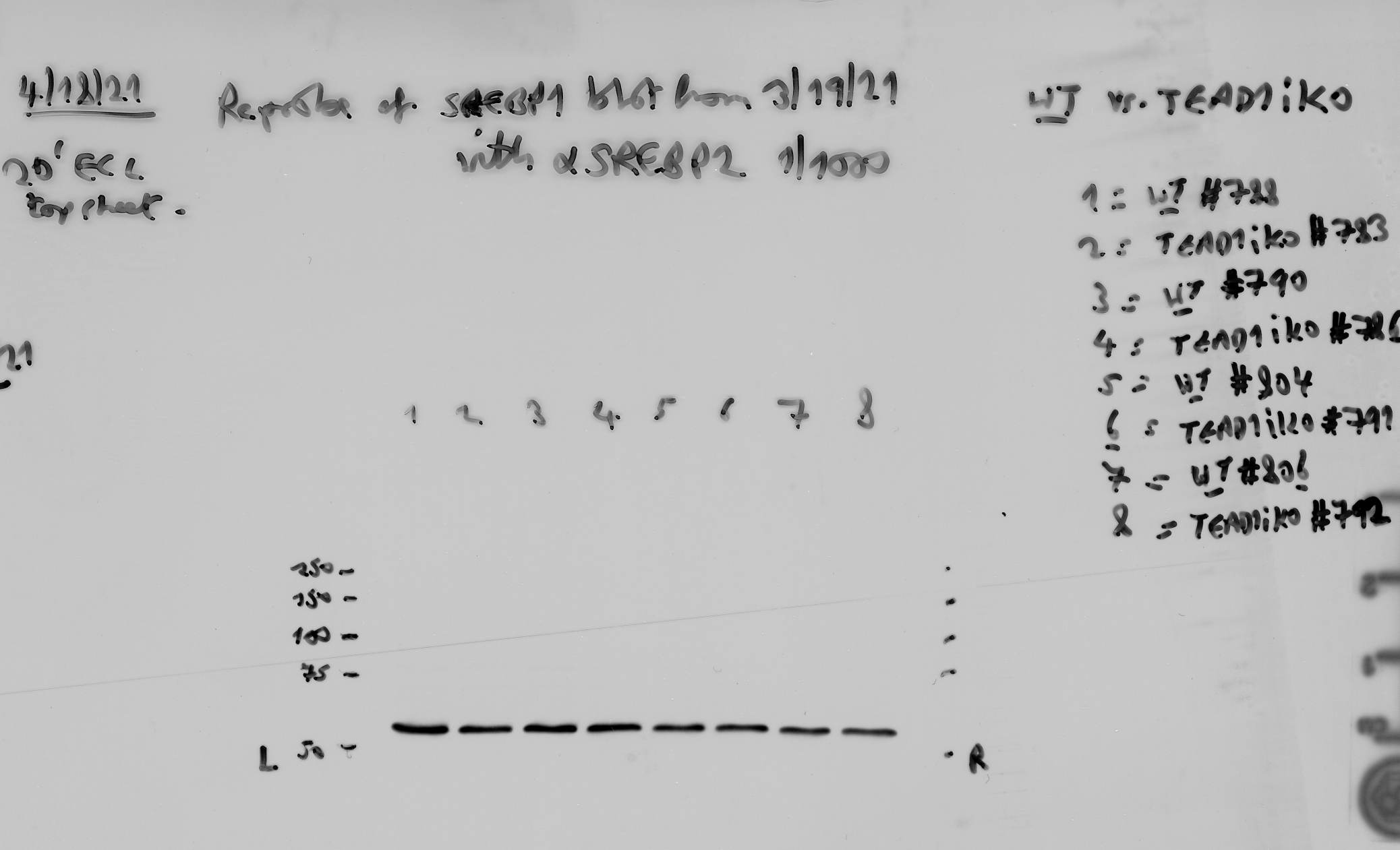

Supplement: Figure 7—source data 1. [file elife-87394-fig7-data1.zip › Fig 7 source data 1/Fig 7E blots and prism files/Tead1 iKO SREBP2/uncropped.tif]
